# Diversity at single nucleotide to pangenome scales among sulfur cycling bacteria in salt marshes

**DOI:** 10.1128/aem.00988-23

**Published:** 2023-10-26

**Authors:** Sherlynette Pérez Castro, Elena L. Peredo, Olivia U. Mason, Joseph Vineis, Jennifer L. Bowen, Behzad Mortazavi, Anakha Ganesh, S. Emil Ruff, Blair G. Paul, Anne E. Giblin, Zoe G. Cardon

**Affiliations:** 1 The Ecosystems Center, Marine Biological Laboratory, Woods Hole, Massachusetts, USA; 2 Crop and Soil Sciences, University of Georgia, Athens, USA; 3 Thomas H. Gosnell School of Life Sciences, Rochester Institute of Technology, Rochester, New York, USA; 4 Department of Earth, Ocean and Atmospheric Science, Florida State University, Tallahassee, Florida, USA; 5 Department of Marine and Environmental Sciences, Marine Science Center, Northeastern University, Nahant, Massachusetts, USA; 6 Department of Biological Sciences, University of Alabama, Tuscaloosa, Alabama, USA; 7 Bay Paul Center, Marine Biological Laboratory, Woods Hole, Massachusetts, USA; Georgia Institute of Technology, Atlanta, Georgia, USA

**Keywords:** sulfur-oxidizing bacteria, sulfate-reducing bacteria, site-specific genetic diversity, diversity-generating retroelement, single-nucleotide polymorphism, pangenomics

## Abstract

Sulfur-cycling microbial communities in salt marsh rhizosphere sediments mediate a recycling and detoxification system central to plant productivity. Despite the importance of sulfur-cycling microbes, their biogeographic, phylogenetic, and functional diversity remain poorly understood. Here, we use metagenomic data sets from Massachusetts (MA) and Alabama (AL) salt marshes to examine the distribution and genomic diversity of sulfur-cycling plant-associated microbes. Samples were collected from sediments under *Sporobolus alterniflorus* and *Sporobolus pumilus* in separate MA vegetation zones, and under *S. alterniflorus* and *Juncus roemerianus* co-occuring in AL. We grouped metagenomic data by plant species and site and identified 38 MAGs that included pathways for sulfate reduction or sulfur oxidation. Phylogenetic analyses indicated that 29 of the 38 were affiliated with uncultivated lineages. We showed differentiation in the distribution of MAGs between AL and MA, between *S. alterniflorus* and *S. pumilus* vegetation zones in MA, but no differentiation between *S. alterniflorus* and *J. roemerianus* in AL. Pangenomic analyses of eight ubiquitous MAGs also detected site- and vegetation-specific genomic features, including varied sulfur-cycling operons, carbon fixation pathways, fixed single-nucleotide variants, and active diversity-generating retroelements. This genetic diversity, detected at multiple scales, suggests evolutionary relationships affected by distance and local environment, and demonstrates differential microbial capacities for sulfur and carbon cycling in salt marsh sediments.

**IMPORTANCE:**

Salt marshes are known for their significant carbon storage capacity, and sulfur cycling is closely linked with the ecosystem-scale carbon cycling in these ecosystems. Sulfate reducers are key for the decomposition of organic matter, and sulfur oxidizers remove toxic sulfide, supporting the productivity of marsh plants. To date, the complexity of coastal environments, heterogeneity of the rhizosphere, high microbial diversity, and uncultured majority hindered our understanding of the genomic diversity of sulfur-cycling microbes in salt marshes. Here, we use comparative genomics to overcome these challenges and provide an in-depth characterization of sulfur-cycling microbial diversity in salt marshes. We characterize communities across distinct sites and plant species and uncover extensive genomic diversity at the taxon level and specific genomic features present in MAGs affiliated with uncultivated sulfur-cycling lineages. Our work provides insights into the partnerships in salt marshes and a roadmap for multiscale analyses of diversity in complex biological systems.

## INTRODUCTION

Sulfur-cycling bacteria are central to the function of salt marsh ecosystems worldwide (1). Organic carbon-rich sediments and sulfate-rich tidal waters favor sulfate-reducing microbial communities, playing a key role in the organic carbon flux (2). These communities produce sulfide as a byproduct, which can be toxic to plant roots (3 – 5). Microorganisms that oxidize sulfide (6) can detoxify rhizosphere sediments for plants. Thus, the metabolism of S-cycling microbes is a major contributor to high rates of marsh plant productivity (7 – 10). The tremendous diversity of sulfur-cycling bacteria has made cross-site comparisons of their broad genomic content difficult (11). Studying the genomic diversity of S-cycling microbes requires deeply sequenced metagenomic data to reveal gene function and phylogenetic relationships (12).

Salt marshes distributed along the eastern coast of the United States are characterized by dominant vegetation regulated by climatic, tidal, and edaphic factors. Salt marsh plants are usually distributed along an elevation gradient depending on their adaptability and tolerance to reduced sediments and saline conditions. Typical species located from low to high marsh include *Sporobolus alterniflorus* (formerly *Spartina alterniflora*), *Sporobolus pumilus* (formerly *Spartina patens*), and *Juncus roemerianus. S. alterniflorus* is most tolerant of the frequent flooding and high salinity characterizing the intertidal zone and low elevations (13, 14). In New England, *S. pumilus* is dominant in the less frequently flooded high marsh zone (15). In Alabama, *J. roemerianus*, though often in high marsh, can mix with *S. alterniflorus* (16).

Over the past several decades, examination of microbial communities by sequencing 16S rRNA genes has identified a wide range of sulfur-cycling bacterial lineages within marsh sediments. Sulfate reducers (SRBs) belong to bacterial phyla such as *Desulfobacterota* (formerly *Deltaproteobacteria*), *Acidobacteriota*, and *Bacteroidota* (17, 18), while sulfur oxidizers (SOXs) are often found in the phyla *Pseudomonadota* (formerly *Proteobacteria*), *Campylobacterota* (formerly *Epsilonproteobacteria*), and *Bacteroidota* encompassing orders *Chlorobiales*, *Chromatiales*, *Rhizobiales*, and *Rhodobacterales* (6, 19, 20). In a biogeographic survey of SRB in multiple East Coast salt marsh sediments, Angermeyer et al. (21) found that the distribution of 16S rRNA genes varied with the environment and geographic distance, but the dissimilatory sulfite reductase gene (*dsrA*), a marker for sulfate reducers, did not. Several studies have also reported the distribution of SOX as a function of temperature, salinity, oxygen, and salt marsh plant root microenvironments (22 – 24). New metagenomic approaches are now emerging as a powerful tool to detect genetic and genomic heterogeneity allowing comparisons, even in highly diverse microbial populations (25, 26).

**Fig 1:**
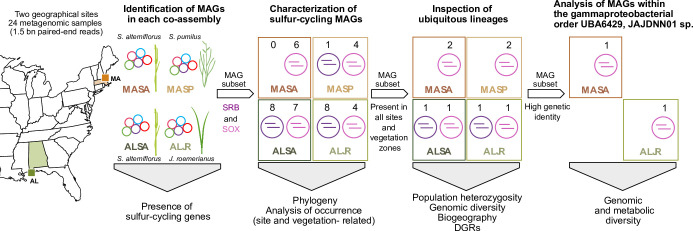
Overview of study sites and metagenomic workflow. Rhizosphere sediment samples were collected in Alabama under *J. roemerianus* (ALJR) and *S. alterniflorus* (ALSA) and in Massachusetts under *S. alterniflorus* (MASA) and *S. pumilus* (MASP). Twenty-four metagenomes yielded 38 MAGs for S-cycling bacteria (17 SRB and 21 SOX). Ubiquitous lineages were used to investigate genomic diversity.

Here, we focus on the sulfur-cycling microbial communities inhabiting two contrasting salt marshes differing in type of sediment (rich in organic matter/fine), latitude (North/South), and vegetation (*S. alterniflorus* and *S. pumilus*/*S. alterniflorus* and *J. roemerianus*). We explored the occurrence of diverse metagenome-assembled genomes (MAGs) encoding genes for enzymes catalyzing dissimilatory sulfate reduction or sulfur oxidation (1) across sites and under different plant taxa. We then characterized their genomic diversity from single nucleotide to pangenome scales (Fig. 1). We investigated shared features and site- and vegetation-specific genetic diversity, providing insights into biogeographic patterns and functional diversity.

## RESULTS

### Data set comparison and identification of MAGs

**Fig 2:**
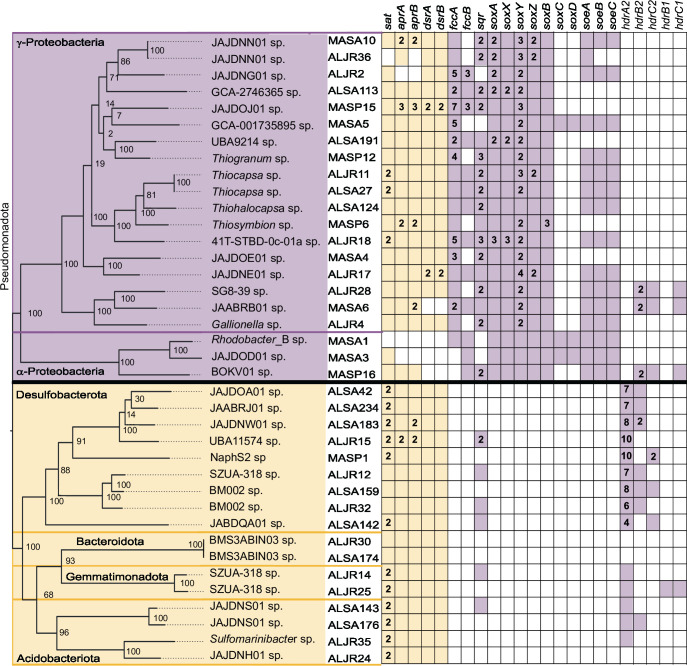
Core sulfur-cycling genes detected in MAGs from Alabama and Massachusetts salt marsh samples. Key genes encoded dissimilatory sulfate reduction (yellow) and sulfur oxidation (purple). MAGs are organized by their GTDB-tk taxonomy, and named by site (AL or MA), vegetation, and bin identification number. Multiple copy genes are given as numbers.*aprAB*, adenylylsulfate reductase, subunit A/B; *dsrAB*, dissimilatory sulfite reductase alpha/beta subunit; *fccA*, cytochrome subunit of sulfide dehydrogenase; *fccB*, sulfide dehydrogenase [flavocytochrome c] flavoprotein chain; *sat*, sulfate adenylyltransferase; *sqr*, sulfide:quinone oxidoreductase; *soxAX*, L-cysteine S-thiosulfotransferase; *soxY*, sulfur-oxidizing protein; *soxB*, S-sulfosulfanyl-L-cysteine sulfohydrolase; *soxC*, sulfane dehydrogenase subunit; *soxD*, S-disulfanyl-L-cysteine oxidoreductase; *soeABC*, sulfite dehydrogenase (quinone) subunit; *hdrA2B2C2B1C1*, heterodisulfide reductase subunit A2/B2/C2/B1/C1.

**Fig 3:**
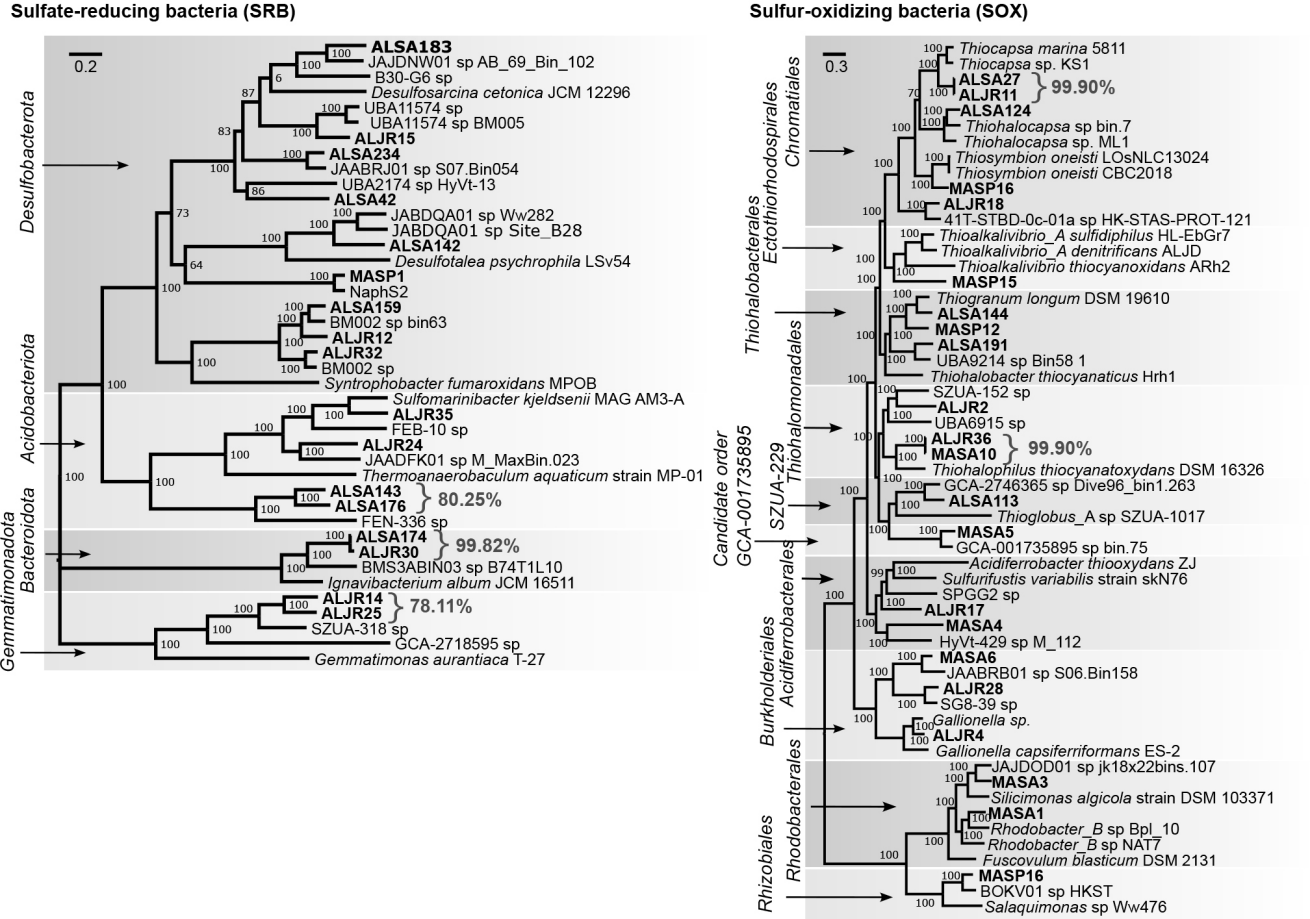
Maximum likelihood phylogenetic trees of sulfate-reducing (left) and sulfur-oxidizing (right) MAGs from Alabama and Massachusetts salt marshes, and their closest relatives, based on analysis of single-copy genes. Phyla are shown on the left of each tree.

In our study, we characterized the distribution and diversity of sulfur-cycling microbial communities across 24 samples collected from sediments under *S. alterniflorus* (SA) and *S. pumilus* (SP) in separate Massachusetts (MA) marsh zones, and under *S. alterniflorus* (SA) and *J. roemerianus* (JR) co-occurring in Alabama (AL) (Table S1). Metagenomic samples were co-assembled according to geographical site and dominant vegetation on the collection site. The four co-assemblies yielded 251,034 (ALJR), 249,343 (ALSA), 70,380 (MASA), and 100,526 (MASP) contigs, with similar N50 values (~7 kb) (Table S2). Binning of the co-assemblies resulted in 118 MAGs [>90% completeness, <5% contamination, quality score (defined as completeness minus five times its contamination) >65], with 38 identified by their gene content as sulfur cyclers (Fig. 2; Supplemental Data 1 and 2) and classified within the bacterial phyla *Acidobacteriota*, *Bacteroidota*, *Desulfobacterota*, *Gemmatimonadota*, and *Pseudomonadota* (Fig. 3; Table S3). Seventeen of the 38 S-cycling MAGs were sulfate reducers (each one including genes *sat*, *apr*AB, and *dsr*AB) belonging to uncultured lineages (Fig. 3; Tables S3 and S4). All but one of these SRB MAGs were assembled from AL samples. The sulfide:quinone oxidoreductase gene (*sqr*) was found in 6 of the 17 SRB MAGs (Fig. 2). Twenty-one of the 38 MAGs were sulfur oxidizers with genes encoding the truncated thiosulfate oxidation system (Fig. 2). Approximately equal numbers of MAGs were assembled from AL and MA samples. All were in the phylum *Pseudomonadota* (Table S3). Two MAGs (ALJR36 and MASA10) were affiliated with the uncultivated lineage UBA6429 (JAJDNN01 sp.), which was the only lineage assembled in both MA and AL samples. All but six SOX (three gamma- and all three alpha-proteobacterial MAGs) harbored *sat*, *apr*AB, and *dsr*AB (Fig. 2). The majority of MAGs encoded *sqr*, but three lacked *sqr* and encoded *soxCD* genes (Fig. 2). Genes for sulfite (*soeABC*) and sulfide (*fccAB*) dehydrogenase were variably represented (Fig. 2). Genes for the heterodisulfide reductase (*hdr*) subunits B2, C1, and C2 were found in three SOX MAGs (SG8-39 and *Rhizobiaceae* families).

Two SOX MAGs (ALJR17 and MASP15) contained multiple copies of *dsr*AB genes. Visual inspection of the contigs encoding the genes confirmed that in all cases, the copies of *dsrA* were located near copies of *dsrB*. Phylogenetic analyses placed both *dsrAB* copies in ALJR17 clustering together (Fig. S1), consistent with a gene duplication (nucleotide identity 83.9% and 85.3%). Its placement within *Gammaproteobacteria* was consistent with the phylogeny presented in Fig. 2. MASP15 gene copies were highly divergent (nucleotide identity 56.5% and 62.3%) with one copy of *dsrAB* placed outside of the *Gammaproteobacteria* branch (Fig. S1), suggesting a lateral gene transfer (LGT) event.

### Distribution of sequences from the 38 MAGs across samples

**Fig 4:**
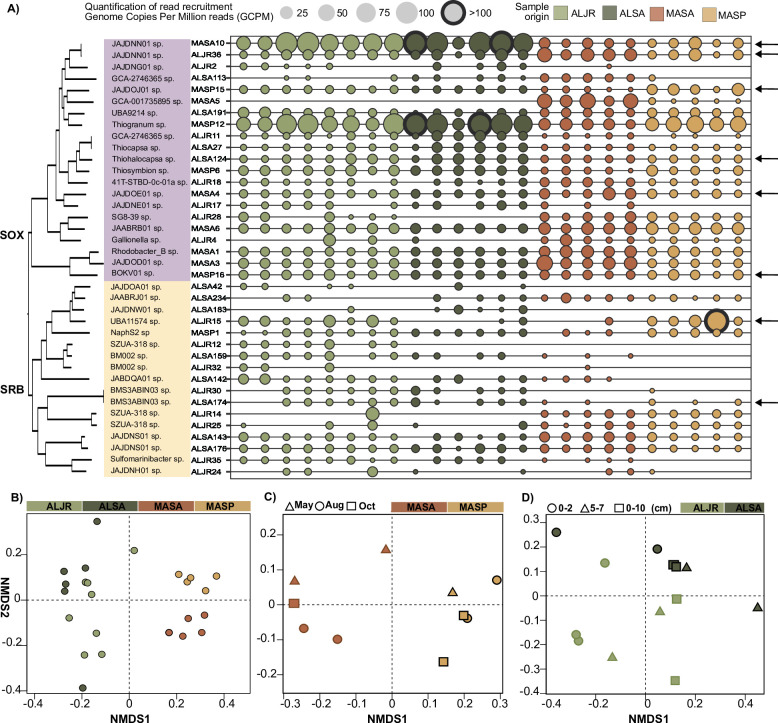
Distribution of sulfur-cycling primary MAG sequences across sites and vegetation zones. (**A**) MAG abundance estimates across AL and MA samples, with coverage values standardized by library size and contig length and expressed as genome copies per million reads (GCPM). Arrows indicate the MAGs used for pangenomic analyses. (**B–D**) Non-metric multidimensional scaling (NMDS) analyses comparing MAGs distribution as expressed as GCPM in: (**B**) Alabama and Massachusetts salt marshes, (**C**) Massachusetts salt marshes by month, and (**D**) Alabama salt marshes by depth.

MAG abundance estimates (see Materials and Methods section) revealed a higher abundance of SOX MAGs than SRB MAGs across all samples [average 11 genome copies per million (GCPM) for SOX, vs average 3 GCPM for SRB]. Various site- and vegetation-specific patterns emerged in MAG abundance estimates. The presence of MASA5 (GCA-1735895 sp.) was only detected in MA samples and was enriched in sediments inhabited by *S. alterniflorus* (Fig. 4A; Fig. S2). Among sulfate reducers, ALSA176 (*Acidobacteriota* Fen-336 family, JAJDNS01 sp.) was the only MAG that recruited reads in all samples (average six GCPM, Fig. 4A), and closely related ALSA143 (also in the Fen-336 family, ANI 80.25%) recruited reads from all but one sample (Fig. 4A). Sequences from *Syntrophobacteria* class BM002 MAGs (ALJR32 and ALSA159) were poorly represented in MA samples vegetated with *S. pumilus* (Fig. 4A).

Site- and vegetation-specific patterns were also revealed in the NMDS analysis. We observed a possible separation by site (MA or AL; Fig. 4B) and by vegetation within site (Fig. 4B), by the month that the sample was collected (MA only, Fig. 4C) and by the depth of sediment (AL-only, Fig. 4D). Permutational multivariate analysis of variance (PerMANOVA) post hoc comparisons detected significant differences in between MASP and MASA samples when compared to each other and to ALJR (*P* = 0.01) and ALSA (*P* = 0.01, Fig. 4B), but no significant differences were found between ALJR and ALSA samples (*P* = 0.15, Fig. 4B). No significant patterns were detected by sampling month in MA (Fig. 4C). In AL (Fig. 4D), 0–2 cm depth samples were significantly different from 5 to 7 cm and 0–10 cm samples (*P* = 0.045). MAG abundance estimates in metagenomic data from unvegetated creek bed sediment samples also revealed more SOX than SRB MAGs across all samples (Fig. S3, Supplemental Data 4). In several instances, MAG distribution showed variation as a function of salinity. For example, the GCPM values of *Burkholderiales* MAGs (ALJR4, ALJR28, and MASA6) were observed to decrease with increasing salinity. *Thiogranum sp*. (MASP12) GCPM values increased with increasing salinity (Fig. S3).

We also observed a spatial pattern in the percentage of reads recruited by the MAGs contigs (Fig. S4 to S6). When aligning AL metagenomic reads to AL MAGs, 99% of the contigs were mapped. When aligning MA *S. alterniflorus* reads, this was reduced to 92% and further reduced to ~75% when aligning MA *S. patens* reads. We did not observe such a pronounced pattern with MA MAGs as percentages ranged from 95% to 100% when aligning MA reads and 92% to 95% when aligning AL reads (Supplemental Data 3).

### Contig-based analysis of genomic variability

**Fig 5:**
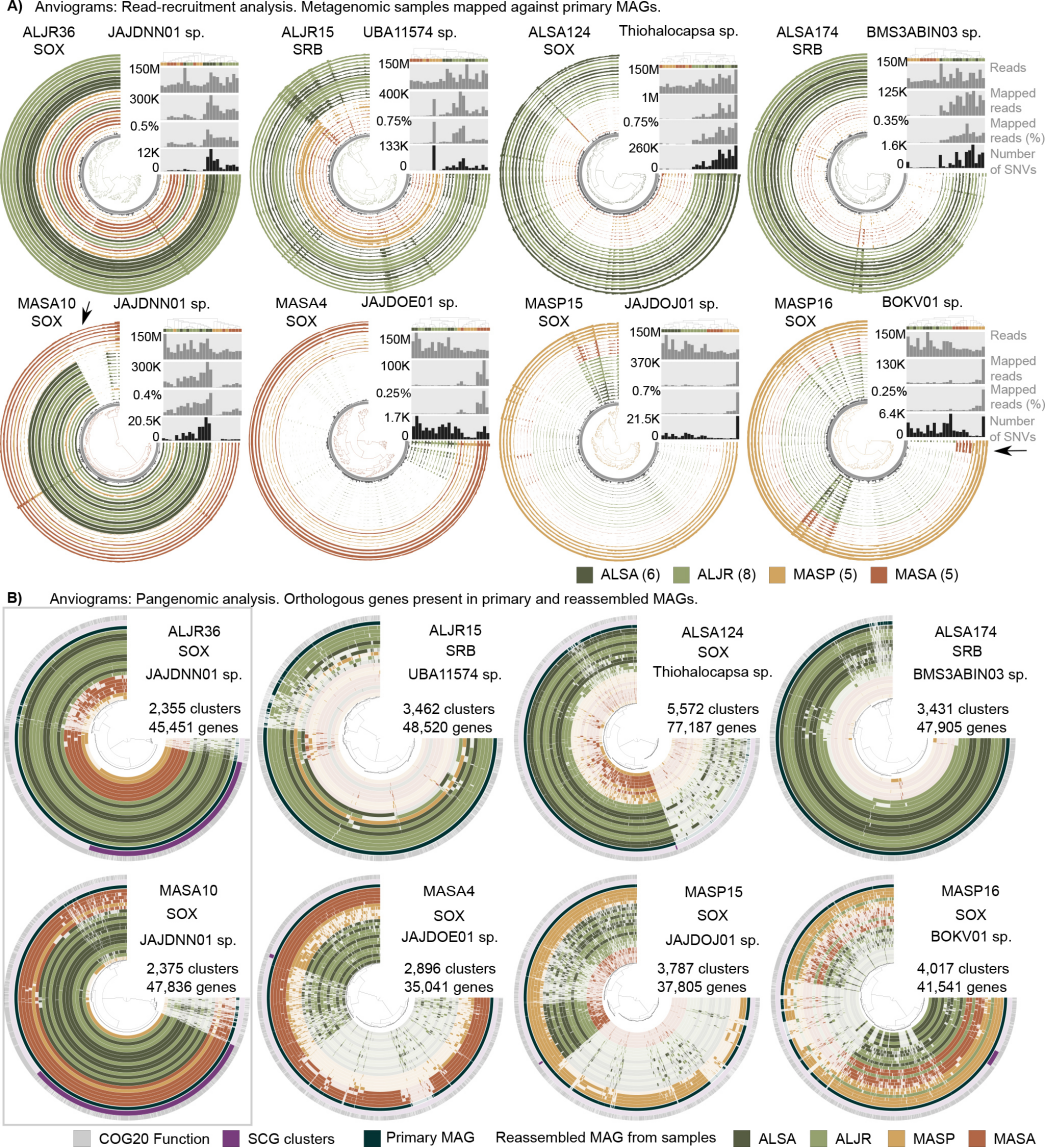
(**A**) Anvi’o coverage profiles of eight selected sulfur-cycling bacteria MAGs. Outer circles represent the contig coverage (as mean coverage) of each of the 24 salt-marsh metagenomic samples included in this analysis. Inner rings represent GC content (dark gray) and contig length (gray). Contigs are clustered (inner tree) based on the sequence composition and differential coverage using Euclidean distance and Ward hierarchical clustering method. Sample order (rings) was determined using a clustering method based on the mean coverage and each ring is color-coded according to site and vegetation type. Bar plots represent (top to bottom) the total number of reads in each library, the total number of reads mapped to each respective MAG, the percentage of mapped reads, and the total number of single-nucleotide variants (SNVs). (**B**) Anvi’o pangenomic analysis of eight sulfur-cycling bacteria MAGs. In each ring, each radial line represents a gene. Gene order was determined using Euclidean distance and Ward clustering method based on presence/absence of a gene cluster across MAGs. Annotation based on COG 2020 is indicated in gray and single-copy core genes in purple. Primary MAG is shown in dark teal blue. Each of the remaining circles represents a reference-guided reassembled MAG from each of the salt-mash metagenomic samples included in this analysis. Samples are color-coded according to geographical site and vegetation.

We selected eight MAGs that were found across all samples (identified by arrows, Fig. 4A) for genomic variability analyses. We observed a relative uniformity in GC content (inner ring) and mean read coverage (colored rings) across the contigs forming each bin (Fig. 5A). Overall, read recruitment to contigs was influenced, but not solely governed by sample sequencing depth (see gray histograms, Fig. 5A, Fig. S7 to S14). While sequence quality, contig length, assembly method, genetic diversity, and experimental design can influence read recruitment; here, we observe an effect on the geographical origin of the sample. Each MAG was better represented in the pool of samples originally used for its assembly and was similarly abundant in the samples of a given site (Fig. 5A). Except in the case of the sediments inhabited by *S. pumilus,* here a MAG could be highly abundant just in one sample. This seems to suggest that in this area microorganisms might have a greater spatial heterogeneity than those areas inhabited by *S. alterniflorus* and *J. roemerianus* (Table S5).

**Fig 7:**
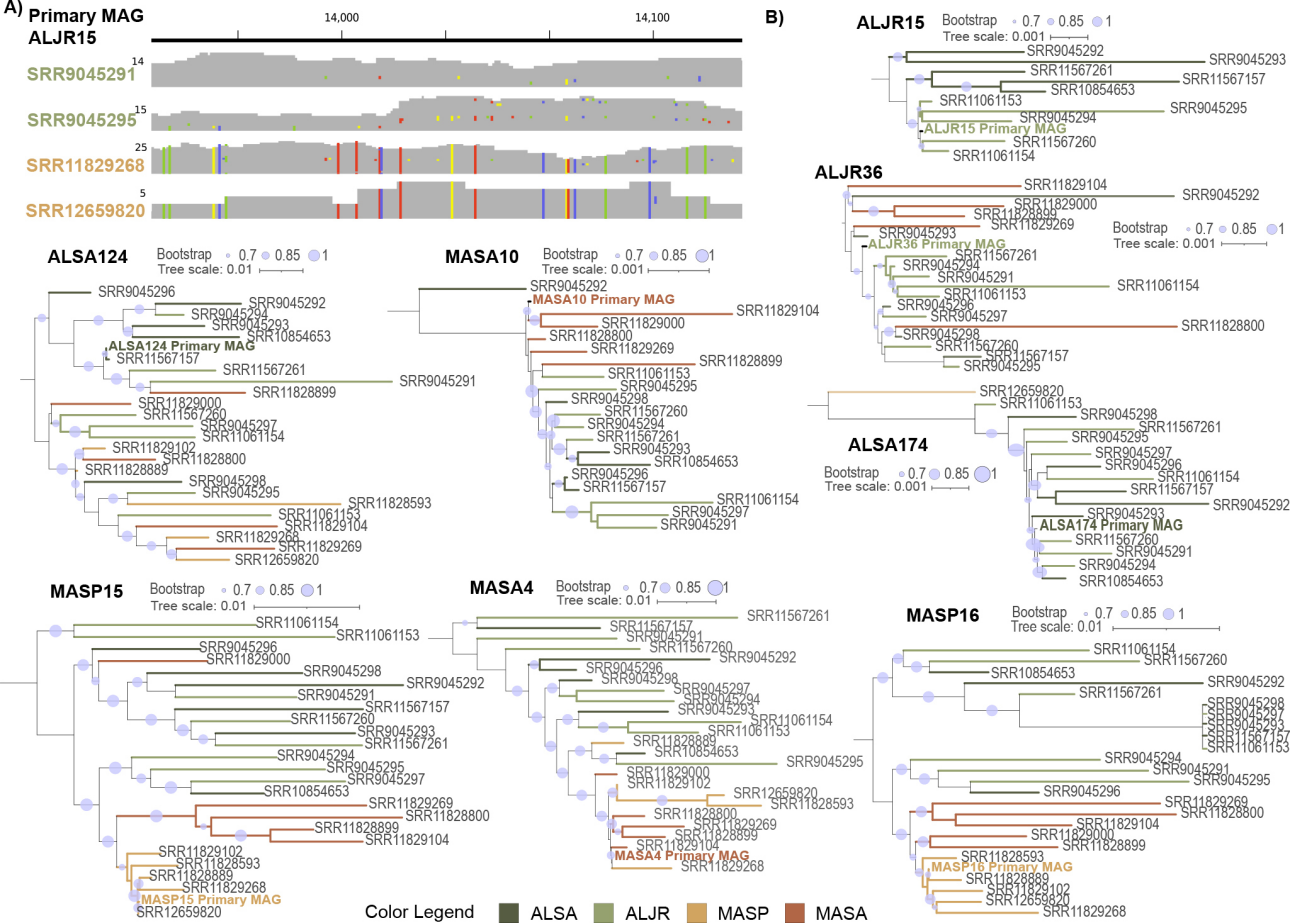
Biogeographic and vegetation-specific distributions of genomic variability. (**A**) Example aggregated view of metagenomic reads from different samples mapped to the MAG ALJR15. Samples collected in Alabama matched the reference while those collected in Massachusetts in sediments dominated by *S. pumilus* displayed numerous fixed mismatches. Mismatches are indicated with colors: green, T; yellow, G; blue, C; red, A. (**B**) Trees depicting phylogenetic relationships among reassembled sample-specific metagenomes. Trees were built using single-copy genes, housekeeping genes, and sulfur-cycling genes. Tree branches are color-coded according to site and vegetation.

Patterns of recruitment varied across MAGs. For example, UBA6429 MASA10 contained an 80.4 kbp region, encoding 86 genes, that was only detected in samples collected in Massachusetts under *S. alterniflorus* (Fig. 5A, arrow). This MASA-exclusive region encodes duplicated copies of genes involved in the biosynthesis of molybdenum cofactor (Moco), and it was rich in transport genes (Supplemental Data 6). Moco is essential for the activity of molybdoenzymes involved in the metabolism of sulfur compounds such as the conversion of sulfite to sulfate. *Rhizobiaceae* MASP16 included an 18.5 kb region that was only present in the samples collected in MA, in either *S. alterniflorus* or *S. pumilus* vegetation zones (Fig. 5A, arrow). This region encoded 22 genes, including a second set of genes of the phospholipid transporter system MlaFEDB and the genes *arcC* and *arcA* involved in arginine biosynthesis (Supplemental Data 6).

We investigated single-nucleotide variants (SNVs) by quantifying the divergence of recruited reads from the MAGs (Fig. 5A, Supplemental Data 5). For each of the four AL-derived MAGs, and for UBA6429 MASA10, the number of SNVs (black histograms) was correlated with the number of mapped reads (gray histograms) across all 24 samples (Fig. 5A; Fig. S15C, and Supplemental Data 5). These results revealed a relationship between the sequencing depth (as reads mapped to the reference) and our ability to detect SNVs. For MAG MASA4, MASP15, and MASP16, the AL samples had far more SNVs per mapped read than the MA samples did (Fig. S15). This indicates that at equal sequencing effort and when mapping to a primary MAG originally assembled from MA, the amount of SNV in AL samples exceeded the variability present among MA samples. This means that for each 100,000 mapped reads to MASA4, we detected 646 SNVs for MASA, 4,053 for MASP, while for ALSA and ALJR the number of SNVs were two orders of magnitude higher (>20,000). In case of ALSA124, the number of SNV per 100,000 mapped reads ranged from 30,000 to 50,000 independently of site and vegetation (Fig. S15C, Supplemental Data 5). In the case of ALSA124, we observe similar levels of divergence to a reference MAG among all sites, while in MASA4, we observe an increase in divergence that is not explained by sequencing depth alone.

### Gene-based analysis of sample-specific reassembled genomes

To capture the genomic diversity represented in the different metagenomic samples, sample-specific MAGs were reassembled using a guided approach in which primary MAGs acted as reference. Completeness of these sample-specific MAGs varied widely and was highest for samples that originally generated the primary MAG (Supplemental Data 7). In ALJR15 (*Desulfobacterota*), ALSA124 (*Gammaproteobacteria*), and ALSA174 (*Ignavibacteria*), we observed a high degree of completeness among AL-reassembled MAGs (55–92%), while we observed limited reassembly success among the MA samples. An extreme case was ALSA174 where MA-derived MAGs completeness was calculated at 1–3%. For all the MA MAGs and a single AL (ALJR36), we successfully reassembled sample-specific MAGs from different geographical sites, whether derived from samples collected in MA (55–65%) or AL (75%) (Supplemental Data 7, Table S6). For MASA10 and ALJR36 (*Gammaproteobacteria* UBA6429, JAJDNN01 sp.), sample-specific reassemblies from all sites shared a very large proportion of the gene clusters found in the original primary MAG. Single-copy core genes were readily identified as 42% and 33%, respectively, of the total number of genes (Fig. 5B; Fig. S7 and S12). For the other six MAGs, SCGs were sparse or absent (Fig. 5B; Fig. S8 through S14).

### Hot spots of variability in sample-specific reassembled MAGs

**Fig 6:**
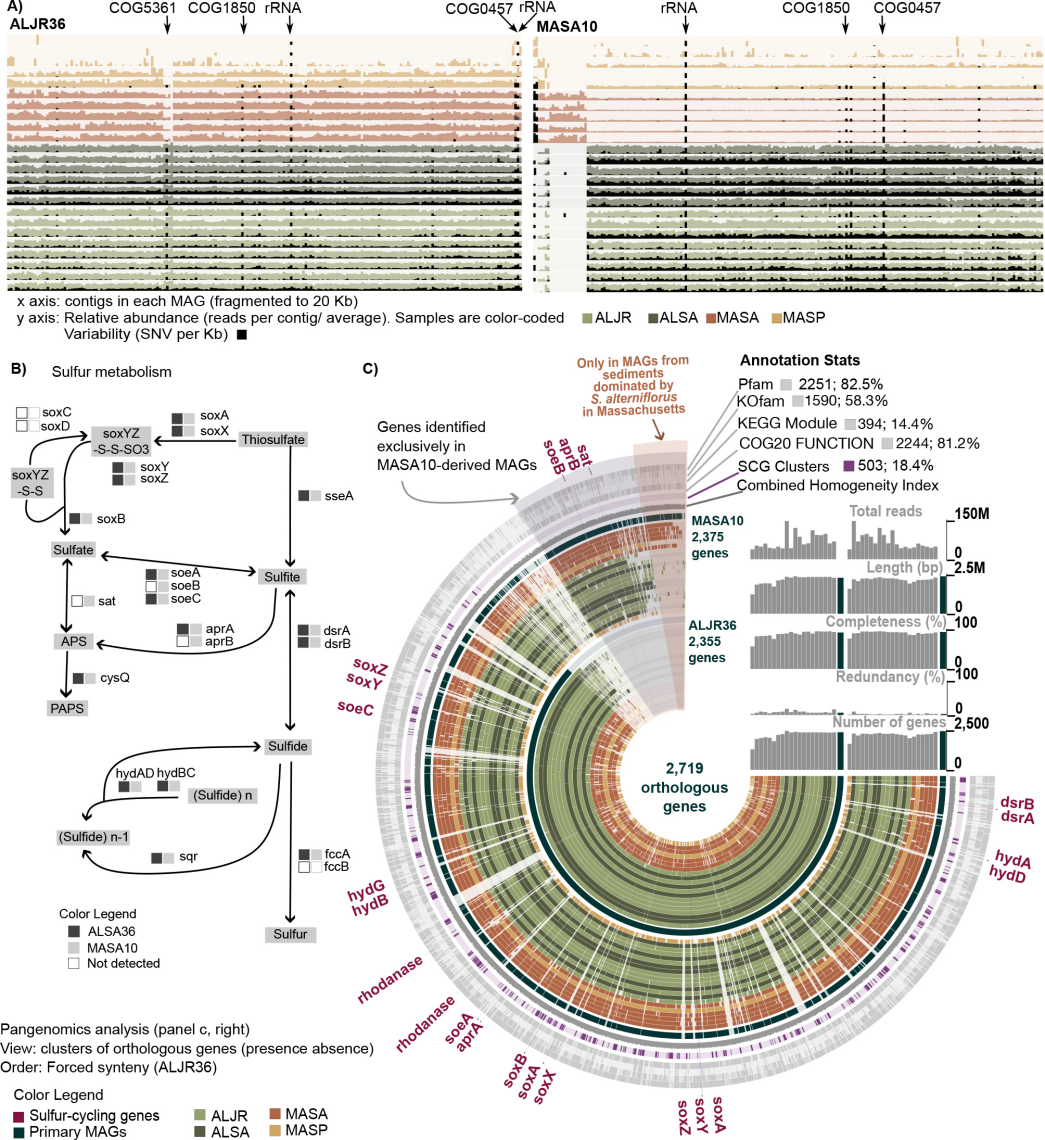
Comparative analysis of genomic and metabolic diversity of two closely related sulfur-oxidizing MAGs (*Thiohalomonadales*) (**A**) Distribution of genetic variability represented as SNV per kilobase (black bars) and calculated for each metagenomic sample. Hotspots of variability include COG5361 (Mobilome), COG1850 (Rubisco-like protein), COG0457 [Tetratricopeptide (TPR) repeat], and rRNA. (**B**) Diagram of sulfur metabolism pathways highlighting the differences in gene content between sample-specific MAGs from the ALJ36 and MASA10 primary MAG groups (adapted from Kegg map00920). (**C**) Pangenomic analysis of metagenomes from group ALJR36 and MASA10. Primary MAGs are indicated by the dark ring and sample-specific reassembled MAGs are color-coded by site and vegetation. Genes clusters are ordered with forced synteny to ALJR36 to highlight the genes exclusively found in the MASA10 group. Annotations, single-copy core genes, and the combined homogeneity index are shown in outer rings.

The close phylogenetic relationship of UBA6429 ALJR36 and MASA10 (Fig. 3, ANI: 99.90%) and coexistence across sites (Fig. 4 and 5B) provided a unique opportunity for the study of genetic diversity and microevolution that might be linked to metabolic capabilities. We show that SNVs remained stable across the MAGs, with the exception of a handful of open reading frames (ORFs) with outlier values (Fig. 6A, arrows). Notably, high variability was observed within prophages and transposons (COG5361), tetratricopeptide repeats responsible for protein-protein interactions or assembly of multiprotein complexes (COG0457), the large subunit of a Rubisco-like protein (COG1850) and genes encoding rRNAs. It is possible that a percentage of reads recruited in this analysis correspond to closely related taxa; however, it is also possible that the high SNV values in multicopy genes such as *rrn* operons reflect the intragenomic diversity in addition to that at the population level. For example, in *rrn*, SNVs accumulate in the hypervariable regions such as V1 and V6 of the 16S gene, with estimated intragenomic divergence ranging from 0.5% to 10% (27). While determination of the number of *rrn* operons was not possible for MASA10 or ALJR36, based on their phylogenetic placement it is expected that these gammaproteobacterial genomes will encode as average 7 ± 2.1 copies of the 16S rRNA gene [rrnDB (28)]. As observed in the previous SNV analysis (Fig. 5A; Supplemental Data 5), SNVs were more abundant across the full genomes of AL-specific MAGs that had more mapped reads (Fig. 6A; dense black bars across full genomes).

### Pangenomic analysis

For further pangenomic analysis, we used reassembled sample-specific MAGs derived from the closely-related MASA10 and ALJR36 primary MAGs (*Gammaproteobacteria* UBA6429, ANI: 99.90%). For each primary MAG, the derived reassembled MAGs were grouped by site (AL or MA) and vegetation type during the comparative analysis. Three MA *S. pumilus* samples were excluded because the completeness of the reassembled genomes was <70% (Supplemental Data 7). All samples were compared in terms of presence/absence or orthologous genes, revealing a shared subset of the genomes (Fig. 6C). Of the 2,719 genes identified in the pangenomic analysis, 2,375 were present in the MASA10 group and 2,355 in the ALJR36 group (Fig. 6C). Functional characterization was relatively complete with 81.2% of the genes identified using COG20, and 82.5% with Pfam. Moreover, 58.3% of the genes were assigned KEGG numbers (gray outer rings, Fig. 6C). A repertoire of 503 SCG was identified across the two groups of metagenomes (purple ring, Fig. 6C). The order of ORFs around the rings in Fig. 6C (which is not related to the order in genomes) visually emphasizes genes found only in reassembled MAGs from MA samples taken under *S. alterniflorus* (labeled MASA), and genes found only in the family of sample-specific MAGs reassembled with primary MAG MASA10 as a guide (labeled MASA10-specific genes).

Despite the large identity among both groups of genomes, 284 genes were present only in ALJR36 reassembled metagenomes and 292 genes in the MASA10 group. Within the MASA10 group, 80 genes were exclusively identified in samples collected in MA in intertidal sediments inhabited by *S. alterniflorus* (Fig. 6C, sector at top of angiogram). Functional enrichment analysis indicated that 11 COG categories were statistically enriched in MAGs from the group ALJR36 and 8 in MAGs from MASA10 (Table S7; Supplemental Data 8). These COG categories include inorganic ion, carbohydrate, and amino acid transport. KEGG enrichment analysis indicated different metabolic capabilities between the MASA10 and ALJR36 groups of metagenomes. Notably, phosphate acetyltransferase-acetate kinase and the reductive citrate cycle (Arnon-Buchanan cycle) pathways were enriched in the ALJR36 group (Table S8; Fig. S16). This was caused by the presence of the genes encoding the enzymes acetate kinase (*AckA*) and aconitate hydratase (*AcnB*) in the ALJR36 group, and the lack of the same genes in the MASA10 group. The MASA10 group was significantly enriched for sulfur-related KEGG modules. Particularly distinguishing are that the ALJR36 group MAGs are missing complete genes encoding subunit B of the sulfohydrogenase *soe*ABC complex, and several key genes (*sat, aprB*) in sulfur-cycling pathways (Fig. 2; Fig. 6C). Although DRAM did not find *soeC* in ALJR36 (Fig. 2), we were able to identify an incomplete copy using Anvi’o (Fig. 6C).

### Phylogenetic analysis indicates structure in genomic variability

Using reassembled MAGs, we conducted phylogenetic analyses of single-copy genes and protein-coding marker genes (Supplemental Data 9), capitalizing on fixed changes at the sequence level (Fig. 5 and 7A) indicative of divergent populations. For the four groups of sample-specific MAGs whose reassembly had been guided by MA primary MAGs (MASA10, MAS15, MASP16, and MASA4), sample-specific MAGs largely fell in well-supported MA (red-brown) and AL (green-olive) clades (Fig. 7B). MAGs from samples from the two MA vegetation zones (*S. alterniflorus* and *S. pumilus*) also tended to fall in separate clades, possibly either because the vegetation was different, the inundation intensity was different, or both. MAGs from AL samples in group ALJR15 clearly segregated by plant type (Fig. 7B), though the plants occurred together under the same inundation regime. Sample-specific MAGs in the other three AL primary MAG groups did not segregate by vegetation or site (Fig. 7B).

### Diversity-generating retroelements are present and active

**Fig 8:**
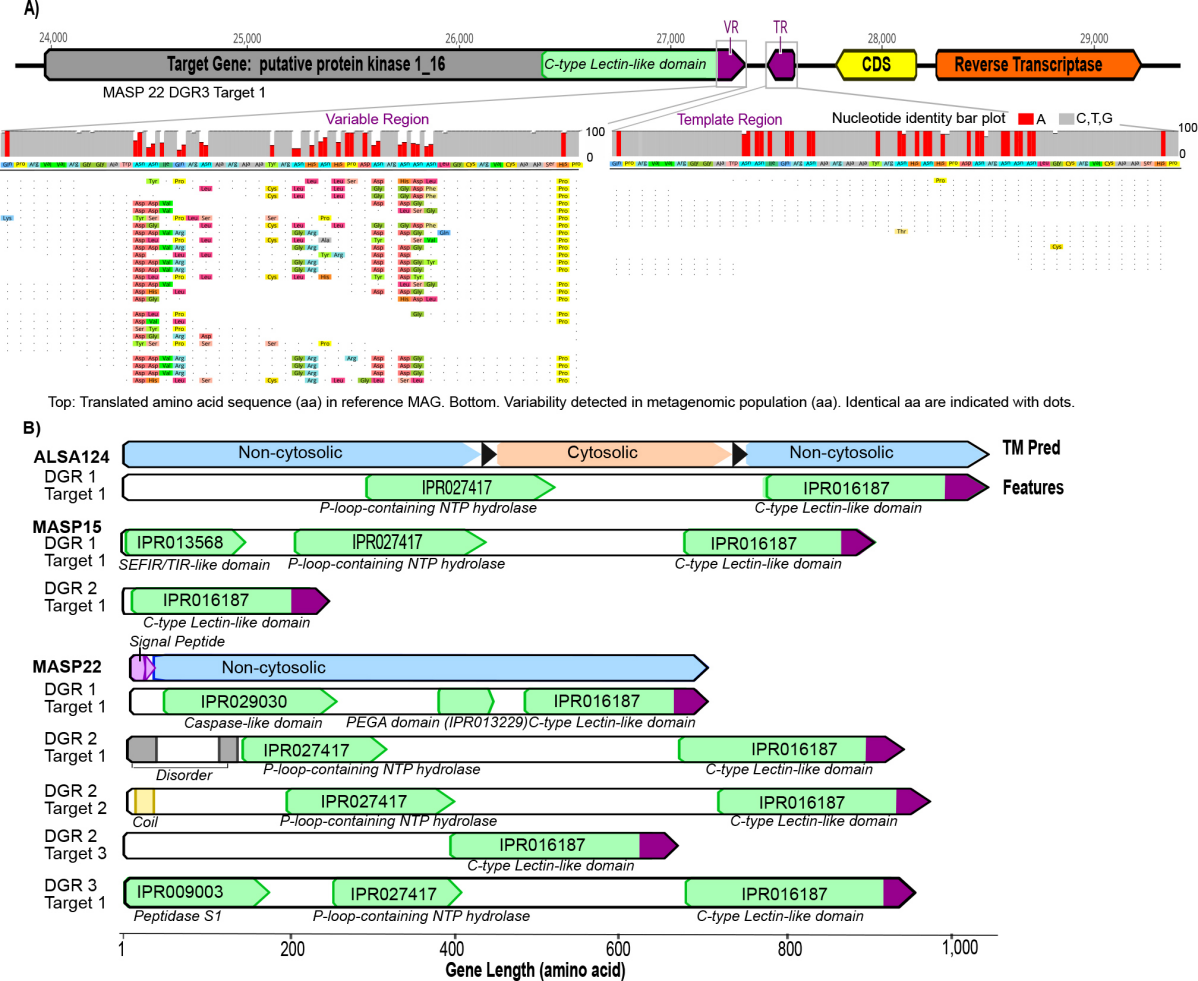
(**A**) Structure of an example DGR identified in MASP22. Diversification of this DGR in this metagenomic sample is indicated by the accumulation of in-frame mutations in the variable region (VR) among the aligned reads. The template region (TR) is conserved both at the nucleotide and amino acid levels. In the bar graphs, the bar size is proportional to the degree of conservation of the nucleotide position. Adenine positions in the VR and TR regions are indicated in red. In the recruited reads, identical residues are indicated with dots, and changes are color coded. (**B**) Structure and domain composition of the target genes containing variable regions (in purple) of the DGRs.

Finally, we investigated the possibility that diversity-generating retroelements (DGRs) contributed to the diversification of the sample-specific MAGs. DGRs use an error-prone reverse transcriptase to generate variability in specific target genes (Fig. 8A). Two sulfur-oxidizing MAGs (ALSA124 and MASP15, Fig. 2), both in the *Gammaproteobacteria*, encoded full DGRs. Beyond the 38 MAGs otherwise targeted in this paper, DGRs were also found in two other *Gammaproteobacteria* in the original 118 MAGs—ALJR7 and MASP22.) ALSA124 and ALJR7 (*Chromatiales*) contain a single DGR and a single target gene. MASP15 and MASP22 (UBA4575) encoded two and three DGRs, respectively, including one (in MASP22) with multiple target genes (Fig. 8B). These new DGR-RTs were compared with previously identified DGRs (29, 30) to determine their phylogenetic placement. The DGR-RTs in MASP and ALSA genomes form a monophyletic clade and are distantly related to DGRs identified from members of *Chlorobi*, *Saprospirales*, and *Cyanobacteria* (Fig. S17).

In all target genes, the variable region of the DGR was encoded in a C-type lectin-like domain. This domain is found in a diverse group of proteins, some of which are involved in protein-protein, protein-lipid, or protein-nucleic acid interactions (31). Notably, two of the DGR targets have predicted extracellular regions, which may function in cell-cell or signal interaction, whereas the remaining six DGR targets are predicted to be localized in the cytosol (Fig. 8B). Energy-related domains such as NTP hydrolases were found in five of the eight target genes. The most obvious instance of DGR activity was detected in MASP22. Adenines in the template region were highly conserved (Fig. 8B). In the DGR target gene, 15 variable codons correspond to template (TR) adenines, which have the potential for DGR-directed non-synonymous substitutions, while not directly recoding to a stop codon (Fig. 8A). These correspond to nine asparagine (AAY) residues, one isoleucine (ATC), one glutamine (CAA), one tyrosine (TAT), two histidines (CAY), and one aspartic acid (GAT) encoded in TR. Taken together, the DGR target region can accommodate up to ~1.97 × 10^14^ amino acid variants. In the mapped reads to this MAG, we observed recoding between 2 and 8 amino acids at each DGR-variable position. This observation suggests that a diversity of ~378 million and ~2 × 10^14^ variants could be randomly rewritten through adenine mutagenesis of this single gene.

## DISCUSSION

Sulfur-cycling organisms are taxonomically diverse and abundant in salt marsh sediments. Comparative metagenomic analyses, from sediments under *S. alterniflorus* and *S. pumilus* in separate MA zones, and under *S. alterniflorus* and *J. roemerianus* co-occuring in AL, enabled the characterization of biogeographic patterns from single nucleotide to pangenome scales.

### Genomic patterns reveal the potential contribution of taxa across environments

We found SRB MAGs containing genes for dissimilatory sulfate reduction (*sat*, *aprAB*, and *dsrAB*) within multiple phyla, with *Desulfobacterota* being dominant (53% of MAGs), as previously observed, in Massachusetts (17), Mississippi (20), and Georgia (32) salt marshes. Other SRB MAGs belonged to *Acidobacteriota*, *Bacteroidota*, and *Gemmatimonadota*, lineages well recognized to contain sulfate reducers (11, 20, 33 – 36). All but one SRB primary MAG were co-assembled from AL samples, perhaps because SRBs are so diverse and the sequencing depth at AL was double that of MA. All sulfate-reducing MAGs belonged to genera without cultivated representatives, underlining the capacity of metagenomic analyses to further expand the recognized diversity and genomic potential of sulfate reducers (2, 18).

The taxonomic distribution of SOX MAGs containing the truncated thiosulfate oxidation system (*soxAXYZB*) was simpler than SRB, falling only within the phylum *Pseudomonadota*, with *Gammaproteobacteria* representing 86% of the genomes. In contrast to the SRBs, 33% of the identified SOX belonged to genera with cultivated representatives. Consistent with previous work (6, 37), *Chromatiales* were well represented overall, and *Thiocapsa* sp. was found overrepresented under *S. alterniflorus* in AL compared to any other sample set, including AL *J. roemerianus* (37). Though all the drivers of these particular differences are not yet known, the associations between marsh plants and bacterial lineages have been suggested to result from differential plant characteristics including root structure and function and their influence on soil properties (24). SOX MAGs showed variations in gene content associated with distinct metabolic steps (1, 38). For example, 67% of SOX MAGs with the truncated *sox* system (lacking *soxCD*) had *soeABC*. This sulfite oxidation gene complex has been reported particularly in organisms lacking *soxCD* that are unable to completely oxidize thiosulfate in the periplasm (39). Genes for dissimilatory sulfite reductase alpha and beta subunits (*dsrAB*) were found in 81% of SOX, lacking particularly in alphaproteobacterial MAGs. Genes encoding reverse *dsrAB*, have been found present in SOX that produce sulfur globules during the oxidation of sulfide and thiosulfate, and lacking in SOX with reduced ability to grow on elemental sulfur (40, 41). We identified two MAGs with multiple copies of the *dsrAB* genes. The sequence similarity and phylogenetic placement of the copies were consistent with a gene duplication in ALJR17 and LGT for MASP15. Recent and ancestral lateral acquisition of *dsr* and *apr* genes has been widely reported in the literature (42 – 44).

Clear patterns emerged in read recruitment from the 24 samples to primary SOX and SRB MAGs. These patterns indicate far more sparse mapping to SRB MAGs no matter the group from which they were co-assembled, possibly reflecting, again, the greater diversity of SRBs in marsh sediment. NMDS analysis of the read-mapping metric GCPM shows strong overall separation between MA and AL samples, no effect of sampling month (variable in MA only), and a distinctive surface (0–2 cm) community in AL. Using phospholipid fatty acid analysis (45), we similarly found that salt marsh microbial communities sampled at the Wadden Sea were influenced by sediment depth but not the months (April, July, and October) of sampling. In a 16S rRNA-based comparison of sediment microbial communities from MA to South Carolina, Angermeyer et al. (46) also detected strong North-South differentiation.

NMDS analysis also clearly shows separation within MA of samples taken under *S. pumilus* and *S. alterniflorus*, which may reflect the influence of the plants themselves, the tidal regime (inundation frequency and depth being low for *S. pumilus* and high for *S. alterniflorus*), or both. Data from Hanley et al. (47) show that in the greenhouse, rhizosphere microbial communities vary even with different MA genotypes of *S. alterniflorus*, but once transplanted back into the marsh, plant-specific differences disappeared, and microbial communities correlated instead with local sediment properties. Rinke et al. (45) also demonstrated that salt marsh zones defined by flooding regimes had distinct microbial communities. The potential for strong influence of the local sediment tidal regime is further suggested by the lack of difference between AL samples taken under *S. alterniflorus* and *J. roemerianus*, which were both rooted in the same area with the same tidal regime.

### Biogeography and potential microdiversification

Local variants were detected among sample-specific MAGs reassembled with guidance from the primary MAGs. Already we had seen that reads mapping to some groups of contigs appeared only in samples from particular locations (e.g., MA samples in the MASP16 group) or even only in samples under one vegetation/tidal regime in MA (e.g., MASA10). Not only did AL and MA S-cycling MAGs separate cleanly on NMDS plots, but also the phylogenetic trees built from sample-specific MAGs showed consistent separation of MA and AL samples except when orthologous genes in MA sample-specific MAGs were rare, as in ALJR15, ALSA124, and ALSA 174. These geographic patterns are consistent with the possibility of distance and climate differences working as a geographical barrier and contributing to allopatric differentiation of microbial communities and/or the emergence of subspecies/strains (48, 49). Drift, dispersal, mutation, and selection are other factors that may explain the decoupled biogeographic patterns (21).

UBA6429 MAGs, ALJR36, and MASA10 had high identity (ANI: 99.90%), high read recruitment across all samples, strong representation of SCG clusters, and yet notable differences in the presence/absence of orthologous genes. The contrasts in genomic content in these two sets of MAGs could point to metabolic differentiation, or even hint at adaptation to different environments or niches. Functional analysis identified that the ALJR36 group was enriched in key enzymes of the reductive citrate cycle (Arnon-Buchanan cycle) (50). This CO_2_ fixation cycle is found in anaerobic and microaerobic autotrophic bacteria (51) and anoxygenic phototrophs (52). Particularly conducive conditions for the reductive citrate cycle’s operation would be provided by the high clay content and low permeability of the sediment in the AL site when compared to the higher permeability organic sediment in MA (53). In contrast, functional analysis identified that the MASA10 group was enriched in a diverse repertoire of genes involved in assimilatory and dissimilatory sulfur cycling including the full set of *soe*ABC, *sat*, and *apr*AB genes. ALJR36 MAGs lack *aprB*, *soeB*, and *sat* genes, potentially significantly altering transformations among sulfate, sulfite, and APS. But, if these MAGs do accurately reflect the genetic repertoire of microbes abundant enough to have supported MAG assembly, such differences in basic gene content could reflect genomic microdiversification contributing to the microbes’ coexistence despite their sharing large portions of their genomes (43, 44). Berben et al. (54) also reported variation in the presence of *soe*ABC and *apr*AB in closely related, cultivated *Thioalkalovibrio* strains. In the case of ALJR36 and MASA10, because these are not isolated organisms, it is not possible to test unique physiologies; there are no cultivated members within the UBA6429 family of sulfur oxidizers. But, we can speculate that differences in gene content could reflect genomic microdiversification contributing to the microbes’ coexistence despite their sharing large portions of their genomes (55, 56). Gene expression and genetic manipulation studies are needed to explore the role and importance of *soe*ABC and *apr*AB genes (54).

Finally, we found DGRs encoded in the MAGs and evidence of their dynamism in the form of extraordinarily elevated non-synonymous substitutions, corresponding to adenine-specific variation of VR encoded in the target genes. Though a specific function of the target genes remains unknown in these MAGs, in all cases, a C-type lectin-like (CLec) domain was identified, which is common in other DGR targets that have a recognizable fold. This suggests that accelerated protein evolution is a common trait for the modular, ligand-binding CLec domain in bacteria (31).

Here, recognizing that MAG construction is limited by genome completeness and possible contamination, we have implemented a conservative approach using multiple methods to explore the functional capacity encoded in genomes of sulfur-cycling bacteria in salt marsh microbial communities. Our results, from single nucleotide to pangenome scales, demonstrate that a tremendous range of genetic information available in metagenomic data sets, even those from microbially diverse systems such as salt marsh sediments, can be harnessed for analysis of biogeographic and biotic patterns in targeted taxa.

## MATERIALS AND METHODS

### Data set collection

Metagenomic data sets were retrieved from the Genomes Online Database (https://gold.jgi.doe.gov/) and were sequenced by the Joint Genome Institute using Illumina NovaSeq. The 24 metagenomes included samples taken from rooted sediment under common dominant plant species in their native range, and all of them have a sequencing depth of >30 million reads per sample (Table S1). All samples consisted of 150 nt paired-end reads that were non-overlapping. The AL data set (Gs0135940 P.I. Olivia Mason), included rhizosphere sediment samples collected under *J. roemerianus* (*n* = 8, total reads ~578 M, abbreviated as ALJR) and *S. alterniflorus* (*n* = 6, total reads ~514 M, abbreviated as ALSA) on Dauphin Island (15). Two to three samples were taken at various depths (all within 0–10 cm) in May during 2015, 2016, and 2017. The elevation of *J. roemerianus* and *S. alterniflorus* patches do not differ and the marsh is flooded on every high tide. The MA data set (Gs0142363, P.I. Jennifer Bowen) included samples taken during May, August, and October from low marsh sediments (0–5 cm depth) under *S. alterniflorus* (*n* = 5, total reads ~231 M, abbreviated as MASA) and from high marsh sediments under *S. pumilus* (*n* = 5, total reads ~208 M, abbreviated as MASP) in the Plum Island Long Term Ecological Research (PIE-LTER) site. The sites differed in the type of sediment with MA being carbon-rich (9) and AL formed by sandy mud (~20% clay) (53).

### Metagenomic assembly, binning, and analyses

Scripts for the bioinformatic pipeline used in this study are available at https://github.com/elperedo/SaltMarshMBL. Samples were grouped by site and vegetation for increased sequencing depth during the initial reconstruction of “primary MAGs.” Reads were assembled using MEGAHIT v.1.2.9 (57) and the quality of the four resultant assemblies was evaluated using MetaQUAST v.5.0.2 (58) (Table S2). Contigs longer than 3,000 bp were binned using MetaWRAP v.1.3.2 binning module (59) with MaxBin2 (60), metaBAT2 (61), and CONCOCT (62). MAGs from each co-assembly were dereplicated using the MetaWRAP v.1.3.2 refinement module (59). Completeness and contamination of each MAG were evaluated with CheckM v.1.0.12 (63). MAGs with >90% genome completeness and <5% contamination were retained for further analyses (64) with an overall quality ≥65 (defined as completeness − 5  ×  contamination) (65). MAGs were annotated using Distilled and Refined Annotation of Metabolism (DRAM) v.beta.2 (66). Sulfur-cycling pathways were curated by searching key sulfur genes in the raw (Supplemental Data 1) and metabolism (Supplemental Data 2) output files provided by DRAM. MAGs corresponding to SRB were identified by the presence of dissimilatory reduction of sulfate to sulfite pathway (*sat*, *aprAB*, and *dsrAB*), and SOX by the truncated thiosulfate oxidation system (*soxAX*, *soxYZ*, and *soxB*) (54). The oxidative versus reductive metabolism of *dsrAB* was distinguished using DiSCO v.1.0.0 (Table S4) (67). The presence of flavocytochrome c complex (*fccAB*), sulfide:quinone oxidoreductase (*sqr*), the sulfite:quinone oxidoreductase complex (*soeABC*), and heterodisulfide reductase (*hdrABC*) was also investigated. Taxonomy and closest phylogenetic neighbors were assigned using GTDB-Tk v.2.3.0 (68). Average nucleotide identity (ANI) was computed by EZBioCloud OrthoANIu Calculator (69). Maximum likelihood phylogenetic trees were generated using the Bacterial and Viral Bioinformatic Resource Center (BV-BRC), Genome Tree Service (70, 71). BV-BRC selects and aligns amino acid and nucleotide sequences from PGFams using MUSCLE (72) and BioPython, respectively. Then, phylogenetic analysis of the concatenated alignments is calculated by RaxML v.8.2.11 (73). Additionally, phylogenetic analyses were performed on alignments of the nucleotide sequence of the *dsrAB and aprAB* genes. Sequences were aligned using MUSCLE v3.8.425 (71) and the tree was built using RAxML v8.2.12 (73).

### Distribution of 38 primary MAGs’ sequences across samples

MAGs abundance across samples was estimated using the metaWRAP v.1.3.2 Quant bin module (59). Quant bin uses Salmon v.1.9.0 (74) to align reads from each sample to the MAGs contigs producing normalized coverage values for each contig. Then, Quant bins estimate the abundance of MAGs in each sample by computing a length-weighted average of the MAG’s contigs coverage values expressed as genome copies per million reads (GCPM). GCPM values are standardized by library size (for every 1,000,000 metagenomic reads) and by contig length. Results below two GCPM were filtered out. Additionally, MAG distribution was estimated in unvegetated salt marsh sediments (Table S9) to compare the site- and vegetation-specific patterns found in this study (see Supplemental Information). We used unvegetated salt marsh sediment samples from the origin of the MA samples that are associated with a salinity gradient (75).

Finally, we quantitatively compared the distribution of sulfur-cycling primary MAG sequences across sites and vegetation (in AL and MA), sample depths (AL only), and sampling month (MA only) using PerMANOVA (76) and post hoc comparisons (77), based on the GCPM values calculated for each MAG. Pairwise statistical analysis of metagenomic profiles was also used to identify primary MAGs for overrepresented in particular sites and/or vegetation zones (78). Non-metric multidimensional scaling analyses were generated using the function metaMDS (76).

### Analysis of genomic diversity

We selected eight primary MAGs that were found consistently across all samples (by abundance estimates) and that varied notably in GCPM values across samples for further analyses. Six were sulfur-oxidizers (ALJR36, ALSA124, MASA10, MASA4, MASP15, and MASP16) and two were sulfate-reducers (ALJR15 and ALSA174). Genomic diversity was analyzed using Anvi’o v.7.1 “hope” following the workflow outlined in reference (79). ORFs for each MAG were identified with Prodigal v.2.6.3 (80) and HMMER v.3.2.1 (81) and annotated with COG20 (82) and GhostKoala (83). Anvi-init-bam was used to sort bam files generated with Bowtie2 v.2.4.2 (84) and calculate coverage and genetic variability metrics. Site-specific variability was analyzed in a subset of representative genes (Supplemental Data 7) including ribosomal proteins, DNA-directed RNA polymerase subunits, translation initiation factor, representative genes of the broad metabolism (85), and sulfur-cycling genes. For each gene, we calculated the average gene entropy using custom R scripts (Supplemental Material).

### Pangenomic analysis of reference-guided, sample-specific, assembled MAGs

A reference-guided reassembly strategy allowed us to reconstruct sample-specific MAGs and capture the genomic variability of a given taxon across all the study sites. We generated new, sample-specific MAGs guided by the selected eight primary MAGs using BWA v.0.7.15 (86) and SPAdes v.3.14.1 (87) using the reassemble_bins module from metaWRAP v.1.3.2 (59). Primary MAG and the derived reassembled genomes were analyzed with the Anvi'o pangenomics pipeline (https://merenlab.org/2016/11/08/pangenomics-v2/). Gene functions of the derived MAGs were assigned using NCBI COG20 (81)⁠, HMM hits from Kofam (88), and EBI’s Pfam database (89). The predicted amino acid sequence for a set of core genes was extracted with anvi-get-sequences-for-gene-clusters (Supplemental Data 9) and used to build maximum-likelihood phylogenetic trees with FastTree v.2.1.3 (90). The module anvi-compute-functional-enrichment was used to identify functions and pathways differentially represented in pan-groups consisting of site-specific MAGs at the various sites and vegetation zones.

### Identification of diversity-generating retroelements in sulfur-cycling bacteria

To investigate possible sources of genetic dynamism, we explored the distribution and activity of DGRs (29). DGRs were identified in binned assemblies with the python package DGRpy (https://pypi.org/project/DGR-package/), which annotates the essential DGR features (retrotranscriptase RT, variable region VR, and template region TR) within 20 kbp windows (29, 30). Putative DGR hypermutation target genes were identified by manual curation of the contigs. The dynamism of the DGRs was detected by visually inspecting the variability of recruited reads against the VR.

## Data Availability

All sequence data are available at NCBI under BioProject IDs PRJNA757271 (this study), PRJNA620418, PRJNA620419, PRJNA620422, PRJNA620423, PRJNA620428, PRJNA620432, PRJNA620433, PRJNA620437, PRJNA620476 , and PRJNA654834 (Massachusetts samples), PRJNA539072 - PRJNA539077, PRJNA570127, PRJNA570128, PRJNA621974, and PRJNA621975 (Alabama samples), and PRJNA814317 (unvegetated creek bed sediment samples). Metagenomic samples are available at the Sequence Read Archive (SRA) and accession numbers can be found in Table S1 (Massachusetts and Alabama samples) and Table S9 (unvegetated creek bed sediment samples). Primary MAGs are available at the Genome Sequence Archive (GSA) and accession numbers can be found in Table S3 (MAGs). Scripts for the bioinformatic pipeline used in this study are available at https://github.com/elperedo/SaltMarshMBL.

## SUPPLEMENTAL MATERIAL

Supplemental Data 1 aem.00988-23-s0001.xlsx DRAM raw annotations.

Supplemental Data 2 aem.00988-23-s0002.xlsx DRAM metabolism summary.

Supplemental Data 3 aem.00988-23-s0003.xlsx Contig-specific coverage values of the Alabama and Massachusetts salt marsh samples.

Supplemental Data 4 aem.00988-23-s0004.xlsx Contig-specific coverage values of the unvegetated salt marsh samples.

Supplemental Data 5 aem.00988-23-s0005.xlsx Anvio profiling.

Supplemental Data 6 aem.00988-23-s0006.xlsx Genes in MASA10 and MASP16 MAGs identified as geographically restricted.

Supplemental Data 7 aem.00988-23-s0007.xlsx Reference-guided metagenome reconstruction.

Supplemental Data 8 aem.00988-23-s0008.xlsx Enrichment analyses (MASA10, ALJR36).

Supplemental Data 9 aem.00988-23-s0009.xlsx Genes selected for phylogenetic tree reconstruction and average gene entropy analysis.

Supplemental Material aem.00988-23-s0010.pdf Supplemental methods and results, Tables S1 to S9, supplemental data file descriptions, and Fig. S1 to S18.

ASM does not own the copyrights to Supplemental Material that may be linked to, or accessed through, an article. The authors have granted ASM a non-exclusive, world-wide license to publish the Supplemental Material files. Please contact the corresponding author directly for reuse.
