# Supplementary material for: Diversity at single nucleotide to pangenome scales among sulfur cycling bacteria in salt marshes
Source: Appl Environ Microbiol. 2023 Oct 26;89(11):e00988-23. doi: 10.1128/aem.00988-23 (PMC10686091; doi:10.1128/aem.00988-23)

Supplemental Material for

Diversity at single nucleotide to pangenome scales among sulfur cycling bacteria in salt marshes

Sherlynette Pérez Castro, Elena L. Peredo, Olivia Mason, Joseph Vineis, Jennifer Bowen, Behzad Mortazavi, Anakha Ganesh, S. Emil Ruff, Blair Paul, Anne E. Giblin, Zoe G. Cardon

This pdf includes:

1. Supplemental Methods
2. Additional Results
3. References
4. Table S1-S9
5. Supplemental Data files description S1-S9
6. Figures S1-S18

1. Supplemental Methods

Note. All scripts used for analysis can be found at https://github.com/elperedo/SaltMarshMBL

Data set collection. All sequence data were downloaded from the National Center for Biotechnology Information (NCBI) Sequence Read Archive (SRA) using the SRA toolkit v.2.10 https://trace.ncbi.nlm.nih.gov/Traces/sra/sra.cgi. Metagenome samples were downloaded as .sra files using prefetch, checked with vdb-validate, and extracted to a readable format (.fastq.gz) using fastq-dump. Initial evaluation of read quality (raw reads) was performed using fastQC v0.11.4 (1). Low-quality reads, unpaired reads, and sequencing adapters were removed using Trimmomatic v0.36 (2). Duplicated, low-quality reads (< 20 average PHRED score and/or Ns), and short reads (<60 bp) were removed using Preprocessing and Information of SEQuence data (PRINSEQ v.0.20.4) (3).

Distribution of primary MAGs genomic content across samples. For each MAG, we also analyzed the distribution of the mapped reads across the different contigs composing each MAG by plotting the read recruitment results, expressed as GCPM (Fig. S4-S6). The individual contig coverage was obtained using a custom-made script to extract the contig-specific coverage values calculated by Salmon v.1.9.0 (4) metaWRAP v.1.3.2 (5) Quant bin module for each sample and MAG (Supplemental Data 3-4).

Analysis of genomic diversity. We used Anvi'o v.7.1 "hope" (available from https://github.com/merenlab/anvio/releases/v7) to profile mapping results, finalize genomic bins, annotate, and visualize results following the workflow outlined in (6). In brief, we used anvi-gen-contigs-database to identify open reading frames (ORFs) with Prodigal v.2.6.3 (7)and HMMER (8) to identify single-single copy core genes of bacterial and archaeal origin. We used anvi-run-ncbi-cogs to annotate the identified ORFs using the NCBI Clusters of Orthologous Genes (COG) database (COG20 release) (9) and KEGG Orthology using GhostKoala (10–12). Gene taxonomy was determined using the top hits against Centrifuge v.1.0.4 (13) and from the Kegg Taxonomy results produced by GhostKoala. The MAG taxonomical identity was resolved using anvi-run-scg-taxonomy. Anvi-init-bam was used to incorporate into Anvi'o the mapping results generated with Bowtie2 v.2.4.2 (14) using sensitive presets (-D 15 -R 2 -N 0 -L 22 -i S,1,1.15) and end-to-end alignment to minimize spurious read recruitment. Anvi-profile with the flag –profile-SCVs was used to process each BAM file and to calculate coverage and genetic variability metrics. Anvi-merge was used to combine profiles from each metagenomic sample and create a merged Anvi'o profile of each MAG in the dataset. Finally, the results of the analysis were visualized with anvi-interactive.

For each profiled primary MAG, we selected a subset of representative genes to analyze site-specific variability using average entropy as a measurement of diversity. The gene subset included the genes encoding the ribosomal proteins, DNA-directed RNA polymerase subunits, and translation initiation factors used by CheckM (15) to calculate metagenome completeness. We also analyzed average entropy for genes representative of the broad metabolism (30), and genes involved in sulfur-cycling processes (see Supplemental Data 9 for the full list). For each of the genes, we identified single codon variants (SCVs) using the program anvi-gen-variability-profile as implemented in Anvi'o (6)run with the flag –kiefl-mode. In all cases, we computed gene-specific coverage statistics. We used custom-made R scripts to filter, summarize and visualize the data generated by anvi-gen-variability-profile. To summarize the variability level of each gene, we created the function average gene entropy. In it, and for each

gene, we calculated the gene average entropy normalized to the gene length. [(entropy.sum/entropy.n)/gene_length]

Reconstruction of reference-guided, sample-specific, assembled MAGs. We generated sample-specific reassembled MAGs using the reassemble_bins module implemented in metaWRAP v.1.3.2 (5). The MAGs selected for further analysis were the same as those previously selected for Anvi'o profiling. In this portion of the analysis, the MAGs generated through the coassembly strategy were used as a general reference to guide sample-specific assemblies using each of the 24 metagenomic samples listed in Table S1. In brief, the reassemble_bins module metaWRAP uses BWA v.0.7.15 (16) to align the reads in a metagenomic sample to a reference MAG followed by a genome reconstruction using SPAdes v.3.14.1 (17). This pipeline uses a parallel approach to reassembly derived from permissive and strict mapping conditions in the BWA step (2 and 5 mismatches respectively). Here, we only considered the results of the reassembly under strict conditions. The number of reads mapped per metagenomic sample to each MAG is presented in Table S5. Initial completeness of the reference-guided, sample-specific, assembled MAGs was estimated using CheckM v.1.0.12 (15).

Pangenomic analysis of the reference-guided, sample-specific, assembled MAGs

We used the Anvi'o pangenomics pipeline [https://merenlab.org/2016/11/08/pangenomics-v2/] for the analysis of the suite of reference-guide genomes reassembled from each of the eight MAGs selected for further analysis. For each bacterial taxon, we stored the primary MAG generated through a coassembly (see Assembly and taxonomic assignment of contigs section) and each of the reference-guided reassembled metagenome in an Anvi'o database using the command anvi-gen-genomes-storage. We identified single-copy genes and rRNAs using anvil-run-hmms. Gene functions were assigned using NCBI COG20 (9), HMM hits from Kofam, and EBI's Pfam database (12). Additionally, tRNAs were scanned using anvi-scan-trnas. Anvi-run-scg-taxonomy was used to confirm taxonomy and anvi-estimate-metabolism to estimate metabolic capabilities.

To create the pangenomes from each metagenome, we used the command anvi-pan-genome which used NCBI's BLAST v.2.11.0 (18) to quantify gene similarity within and between genomes in each database and Markov Cluster algorithm (MCL) (19)to cluster groups of similar genes. We set the flag --min-ocurrence to 2 to remove singletons. The resulting pangenomes were visualized using anvi-display-pan. In the case of the closely related MAGs ALJR36 and MASA10, we completed an additional pangenomic analysis comparing the groups of metagenomes reassembled across samples for each of these sulfur-oxidizer bacteria affiliated with the UBA6429 family. We completed a functional enrichment analysis using the module anvi-compute-functional-enrichment to identify those genes, functions, and pathways over-represented in the pan-groups (reference MAG, and site and vegetation).

Analysis of the phylogenetic relationships among the reference-guided reconstructed MAGs.

We used the command anvi-get-sequences-for-gene-clusters to extract, from each metagenome, the predicted amino acid sequence of a subset of core genes (ribosomic proteins, polymerases, and transcription factors), housekeeping genes, and sulfur-cycling genes (Supplemental Data 9). Amino acid gene sequences were concatenated and samples with a high percentage of missing data in the gene alignments (>90% of missing sites) were removed from the analysis. Maximum-likelihood phylogenetic trees were calculated using FastTree v.2.1.3 (20) as implemented in anvi-gen-phylogenomic-tree.

Identification of Diversity-Generating Retroelements in sulfur-cycling bacteria. DGRs were identified with the python package DGRpy (available at GitHub: https://pypi.org/project/DGR-package/), which provides an integrated pipeline to annotate the essential features, RT, VR, and TR within 20kbp windows, as previously described (21, 22). Genomic sequences were used as the input for DGRpy and open reading frame (ORF) predictions determined using Prodigal v.2.6.3 (7) with -p meta and otherwise default parameters. Next, ORFs were analyzed for RT homology via HMMER v.3.2.1 (8) against a custom RT-HMM profile (21). The genomes were processed into local 20 Kbp regions, extracted with +/- 10 Kbp flanking the RT-like sequence. The RT-proximal sequences were next used to generate short sliding windows as 200 nt fragments that overlap by 50 nt. All 200 nt sequences were compared via BLASTn (23) and the resulting XML was parsed to search for VR/TR pairs in alignments having an HSP score < 100 and alignment length ≥ 60 nt. A VR/TR pair was identified in alignments having mismatches that correspond to adenines (or thymidine if on the reverse strand) for one of the two pairwise sequences. Alignments with i) a relative proportion of 80% A-mismatches (or reverse-strand T-mismatches) along one sequence, relative to other mismatches (G, C, or T) and ii) a minimum of five A-/T- specific mismatches, were considered VR/TR pairs. Since the BLAST xml output contains both full and partial TRs/VRs, sequences were clustered at 100% identity using CD-HIT (24)c, to obtain the full-length representatives. Finally, RTs, VRs, and TRs were manually inspected in the original MAGs to identify the putative DGR hypermutation target genes.

2. Additional results

MAGs occurrence across all vegetated samples. We completed an analysis of GCPM values at the individual contig level. The coverage patterns, as relatively constant values across contigs included in a bin, were similar to those expected in samples containing S-cycling bacteria that were highly similar to the MAGs used as references for mapping reads. This was especially apparent with some of the MAGs reconstructed using samples collected in sediments inhabited by *S. alterniflorus* in Massachusetts (see Supplemental Data 4; MASP12, MASA6, MASA5, MASA4, MASA3, MASA1, ALSA234, ALSA191, ALSA113, ALJR36, ALJR32, ALJR30, ALJR18, ALJR15, ALJR4). As was the case on the vegetated sediments, higher read recruitment in sulfur-oxidizing lineages when compared to sulfate-reducers were also observed in these subtidal metagenome samples. In Particular, we detected the presence of sulfur-oxidizing taxa affiliated with *Alphaproteobacteria* and *Burkholderiales* MAGs across all sites and environments (Fig. S6).

Gene heterogeneity as average entropy. We calculated the average entropy for a selected set of genes (listed in Supplemental Data 9) to get a more accurate picture of the distribution of the genetic heterogeneity in the coding regions of the genomes of these bacterial populations. While the SNV density is useful to identify mismatches to a 'reference' sequence, entropy provides a quantification of the disagreement from the consensus, measuring the diversity in a bacterial population by removing the possible effect of the fixed SNV in a given bacterial population. By focusing on the entropy of single codon variants (SCV) we specifically explored the changes affecting the triplets of nucleotides encoding amino acids, independently if they were synonymous changes or not.

Only four MAGs (ALJR36, ALJR15, ALSA124, and MASA10), specifically in the metagenomic samples collected in Alabama, independently of vegetation type, had enough coverage to fulfill the minimal coverage depth set as in the analysis. Average gene entropy values varied among genes annotated for a given MAG. In most cases, average entropy values were higher for the

genes encoding ribosomal proteins than for housekeeping or sulfur-related genes (Fig. S18 and Supplemental Data 7). Entropy values were not dependent on the average gene coverage as indicated for the similar values of average entropy calculated for a given gene in diverse samples (Fig. S18A, heatmap in purple) despite variations in coverage (Fig. S18A heatmap in orange).

The main result we observed is that for a given gene of a given MAG, the entropy values calculated remained relatively constant across different bacterial populations in different samples analyzed here, independently of the year of collection or the plant dominating the sediments. More interestingly, we identified that the average entropy values of a given gene were MAG-specific. To better showcase these results, we compared the average entropy values of housekeeping genes and sulfur-cycling genes in the SOXs ALJR36 and MASA10 (Fig. S18B) and specifically, using samples collected in Alabama as they were consistently abundant and therefore produced more robust results. The average entropy values, and therefore diversity in the bacterial population, calculated e.g. for the gene Succinate dehydrogenase *Sdh*A−1 (COG1053) were consistently higher for ALJR36 than for MASA10. This was also observed for the genes encoding subunits alpha (K17993) and delta (K17994) of the Sulfhydrogenase (*Hyd*) while in MASA10 the entropy values of the two subunits were similar. In ALJR36 the values were not only much higher but the average entropy value for the alpha subunit almost doubled that for the delta subunit. In the case of the dissimilatory sulfite reductase (*dsr*), the average entropy calculated for the genes encoding subunits alpha (K11180) and beta (K11181) were similar for the bacterial populations matching MASA10 (~0.001). Values of *dsrA* in ALJR36 were also in that range while *dsr*B displayed a considerably lower genetic diversity.

References

1. Andrews S. 2010. FastQC A Quality Control tool for High Throughput Sequence Data. https://www.bioinformatics.babraham.ac.uk/projects/fastqc/. Retrieved 20 November 2022.
2. Bolger AM, Lohse M, Usadel B. 2014. Trimmomatic: A flexible trimmer for Illumina sequence data. Bioinformatics 30:2114–2120.
3. Schmieder R, Edwards R. 2011. Quality control and preprocessing of metagenomic datasets. Bioinforma Oxf Engl 27:863–864.
4. Patro R, Duggal G, Love MI, Irizarry RA, Kingsford C. 2017. Salmon provides fast and bias-aware quantification of transcript expression. 4. Nat Methods 14:417–419.
5. Uritskiy GV, DiRuggiero J, Taylor J. 2018. MetaWRAP—a flexible pipeline for genome-resolved metagenomic data analysis. Microbiome 6:158.
6. Eren AM, Kiefl E, Shaiber A, Veseli I, Miller SE, Schechter MS, Fink I, Pan JN, Yousef M, Fogarty EC, Trigodet F, Watson AR, Esen ÖC, Moore RM, Clayssen Q, Lee MD, Kivenson V, Graham ED, Merrill BD, Karkman A, Blankenberg D, Eppley JM, Sjödin A, Scott JJ, Vázquez-Campos X, McKay LJ, McDaniel EA, Stevens SLR, Anderson RE, Fuessel J, Fernandez-Guerra A, Maignien L, Delmont TO, Willis AD. 2021. Community-led, integrated, reproducible multi-omics with anvi'o. Nat Microbiol 6:3–6.
7. Hyatt D, Chen G-L, LoCascio PF, Land ML, Larimer FW, Hauser LJ. 2010. Prodigal: prokaryotic gene recognition and translation initiation site identification. BMC Bioinformatics 11:119.
8. Finn RD, Clements J, Eddy SR. 2011. HMMER web server: Interactive sequence similarity searching. Nucleic Acids Res 39:29–37.
9. Galperin MY, Wolf YI, Makarova KS, Alvarez RV, Landsman D, Koonin EV. 2021. COG database update: Focus on microbial diversity, model organisms, and widespread pathogens. Nucleic Acids Res 49:D274–D281.
10. Kanehisa M, Goto S, Sato Y, Furumichi M, Tanabe M. 2012. KEGG for integration and

interpretation of large-scale molecular data sets. Nucleic Acids Res 40:109–114.
11. Kanehisa M, Sato Y, Kawashima M, Furumichi M, Tanabe M. 2016. KEGG as a reference resource for gene and protein annotation. Nucleic Acids Res 44:D457–D462.
12. Aramaki T, Blanc-Mathieu R, Endo H, Ohkubo K, Kanehisa M, Goto S, Ogata H. 2020. KofamKOALA: KEGG Ortholog assignment based on profile HMM and adaptive score threshold. Bioinformatics 36:2251–2252.
13. Kim D, Song L, Breitwieser FP, Salzberg SL. 2016. Centrifuge: rapid and accurate classification of metagenomic sequences, version 1.0.4_beta. bioRxiv 26:054965.
14. Langmead B, Salzberg SL. 2012. Fast gapped-read alignment with Bowtie 2. Nat Methods 9:357–359.
15. Parks DH, Imelfort M, Skennerton CT, Hugenholtz P, Tyson GW. 2015. CheckM: assessing the quality of microbial genomes recovered from isolates, single cells, and metagenomes. Genome Res 25:1043–1055.
16. Li H, Durbin R. 2009. Fast and accurate short read alignment with Burrows-Wheeler transform. Bioinformatics 25:1754–1760.
17. Bankevich A, Nurk S, Antipov D, Gurevich AA, Dvorkin M, Kulikov AS, Lesin VM, Nikolenko SI, Pham S, Prjibelski AD, Pyshkin AV, Sirotkin AV, Vyahhi N, Tesler G, Alekseyev MA, Pevzner PA. 2012. SPAdes: A new genome assembly algorithm and its applications to single-cell sequencing. J Comput Biol 19:455–477.
18. Camacho C, Coulouris G, Avagyan V, Ma N, Papadopoulos J, Bealer K, Madden TL. 2009. BLAST+: Architecture and applications. BMC Bioinformatics 10:1–9.
19. Enright AJ, Van Dongen S, Ouzounis CA. 2002. An efficient algorithm for large-scale detection of protein families. Nucleic Acids Res 30:1575–1584.
20. Price MN, Dehal PS, Arkin AP. 2010. FastTree 2 - Approximately maximum-likelihood trees for large alignments. PLoS ONE 5:e9490.
21. Roux S, Paul BG, Bagby SC, Nayfach S, Allen MA, Attwood G, Cavicchioli R, Chistoserdova L, Gruninger RJ, Hallam SJ, Hernandez ME, Hess M, Liu W-T, McAllister TA, O'Malley MA, Peng X, Rich VI, Saleska SR, Eloe-Fadrosh EA. 2021. Ecology and molecular targets of hypermutation in the global microbiome. 1. Nat Commun 12:3076.
22. Paul BG, Burstein D, Castelle CJ, Handa S, Arambula D, Czornyj E, Thomas BC, Ghosh P, Miller JF, Banfield JF, Valentine DL. 2017. Retroelement-guided protein diversification abounds in vast lineages of Bacteria and Archaea. 6. Nat Microbiol 2:1–7.
23. Altschul SF, Madden TL, Schäffer AA, Zhang J, Zhang Z, Miller W, Lipman DJ. 1997. Gapped BLAST and PSI-BLAST: A new generation of protein database search programs. Nucleic Acids Res 25:3389–3402.
24. Li W, Godzik A. 2006. Cd-hit: a fast program for clustering and comparing large sets of protein or nucleotide sequences. Bioinformatics 22:1658–1659.

Table S1. Summary of sampling locations, dominant vegetation, depth, dates, and the number of reads of the selected metagenomic datasets. (AL, Alabama; MA, Massachusetts).

Samples (N)	Site	Vegetation type	Collection date	Depth (cm)	JGI Gp	NCBI SRA	Raw Reads	QC Reads
1	AL	*J. roemerianus*	2015-05	5-7	Gp0344160	SRR9045291	91,291,894	83,691,004
2	AL	*J. roemerianus*	2015-05	0-2	Gp0344159	SRR11061153	77,016,234	66,168,546
3	AL	*J. roemerianus*	2015-05	0-2	Gp0344159	SRR11061154	45,424,663	40,593,738
4	AL	*J. roemerianus*	2016-05	5-7	Gp0344164	SRR904529	96,984,667	89,533,791
5	AL	*J. roemerianus*	2016-05	0-2	Gp0344163	SRR904529	117,191,856	107,792,129
6	AL	*J. roemerianus*	2017-05	0-10	Gp0344168	SRR9045295	66,913,351	59,159,686
7	AL	*J. roemerianus*	2017-05	0-10	Gp0344167	SRR11567261	47,964,230	41,031,419
8	AL	*J. roemerianus*	2017-05	0-10	Gp0344167	SRR11567260	104,213,562	90,031,614
**Total**								**578,001,927**
1	AL	*S. alterniflorus*	2015-05	5-7	Gp0344162	SRR9045293	102,973,665	94,441,225
2	AL	*S. alterniflorus*	2015-05	0-2	Gp0344161	SRR9045292	157,379,608	142,491,993
3	AL	*S. alterniflorus*	2016-05	5-7	Gp0344166	SRR1085465	54,423,170	46,496,251
4	AL	*S. alterniflorus*	2016-05	0-2	Gp0344165	SRR9045298	63,219,940	55,527,119
5	AL	*S. alterniflorus*	2017-05	0-10	Gp0344170	SRR9045296	66,689,192	58,817,185
6	AL	*S. alterniflorus*	2017-05	0-10	Gp0344169	SRR1156715	81,630,750	116,508,305
**Total**								**514,282,078**
1	MA	*S. alterniflorus*	2015-05	0-5	Gp0432379	SRR11828899	48,929,864	43,938,191
2	MA	*S. alterniflorus*	2015-05	0-5	Gp0432385	SRR11829000	47,493,408	42,331,083
3	MA	*S. alterniflorus*	2015-08	0-5	Gp0432391	SRR11829104	52,916,253	46,675,383
4	MA	*S. alterniflorus*	2015-08	0-5	Gp0432397	SRR1182926	57,416,483	50,747,510
5	MA	*S. alterniflorus*	2015-10	0-5	Gp0432373	SRR11828800	53,164,672	47,120,515
**Total**								**230,812,682**
1	MA	*S. pumilus*	2015-05	0-5	Gp0432378	SRR11828889	44,550,785	40,039,222
2	MA	*S. pumilus*	2015-08	0-5	Gp0432390	SRR11829102	41,065,476	36,950,534
3	MA	*S. pumilus*	2015-08	0-5	Gp0432396	SRR1182926	44,913,560	40,622,394
4	MA	*S. pumilus*	2015-10	0-5	Gp0432366	SRR12659820	67,768,852	58,336,509
5	MA	*S. pumilus*	2015-10	0-5	Gp0432372	SRR11828593	35,457,645	31,923,829
**Total**								**207,872,488**
**Sum**								**1,530,969,175**

Table S2. Summary statistics for each of the metagenomic co-assemblies.

Assembly	ALJR	ALSA	MASA	MASP
# contigs (>= 3000 bp)	251,034	249,343	70,380	100,526
# contigs (>= 5000 bp)	103,473	106,678	27,050	41,948
# contigs (>= 10000 bp)	30,574	34,103	7,566	13,088
# contigs (>= 25000 bp)	4,984	6,331	1,301	2,412
# contigs (>= 50000 bp)	937	1,286	281	487
Total length (>= 3000 bp)	1,621,879,533	1,700,038,878	440,187,433	672,932,647
Total length (>= 5000 bp)	1,065,541,187	1,161,729,582	277,515,921	452,063,659
Total length (>= 10000 bp)	573,987,950	669,974,094	146,797,394	256,972,975
Total length (>= 25000 bp)	202,235,813	263,003,851	56,123,242	101,158,766
Total length (>= 50000 bp)	67,990,329	95,150,165	22,126,090	36,675,331
# contigs	251,034	249,343	70,380	100,526
Largest contig	294,481	311,488	311,637	276,711
Total length	1,621,879,533	1,700,038,878	440,187,433	672,932,647
GC (%)	59	59	58	61
N50	6,860	7,514	6,470	7,248
N75	4,265	4,417	4,117	43,432
L50	59,575	55,022	16,873	22,506
L75	136,219	130,825	38,696	53,266
# N's per 100 kbp	0	0	0	0

Table S3. Genome size, GC content, and taxonomic placement of the MAGs identified in this study.

MAG	Accession	Size Mb	GC (%)	Cp %	Ct %	QC	GTDB Taxonomy
**ALJR11**	GCA_022340925.1	3.3	65.5	95	4	73	Pseudomonadota; Gammaproteobacteria; Chromatiales; Chromatiaceae; Thiocapsa sp.
**ALJR12**	GCA_022340905.1	4.1	49.5	97	0	97	Desulfobacterota; Syntrophobacteria; BM002; BM002; BM002 sp.
**ALJR14**	GCA_022340865.1	4.4	69.4	98	1	93	Gemmatimonadota; Gemmatimonadetes; Longimicrobiales; UBA6960; SZUA-318 sp.
**ALJR15**	GCA_022340885.1	3.6	47.3	91	3	74	Desulfobacterota; Desulfobacteria; Desulfobacterales; UBA11574; UBA11574 sp.
**ALJR17**	GCA_022340825.1	2.2	54.4	91	3	78	Pseudomonadota; Gammaproteobacteria; Acidiferrobacterales; JAJDNE01; JAJDNE01 sp.
**ALJR18**	GCA_022340805.1	3.5	64.2	90	0	90	Pseudomonadota; Gammaproteobacteria; Chromatiales; Sedimenticolaceae; 41T-STBD-0c-01a sp.
**ALJR2**	GCA_022340845.1	3.8	50.0	90	2	81	Pseudomonadota; Gammaproteobacteria; SZUA-152; SZUA-152; JAJDNG01 sp.
**ALJR24**	GCA_022340775.1	3.8	66.6	96	0	96	Acidobacteriota; Thermoanaerobaculia; Thermoanaerobaculales; Sulfomarinibacteraceae; JAJDNH01 sp.
**ALJR25**	GCA_022340755.1	4.6	67.7	93	1	87	Gemmatimonadota; Gemmatimonadetes; Longimicrobiales; UBA6960; SZUA-318 sp.
**ALJR28**	GCA_022340705.1	3.6	67.5	97	0	97	Pseudomonadota; Gammaproteobacteria; Burkholderiales; SG8-39; SG8-39 sp.
**ALJR30**	GCA_022340715.1	3.8	35.2	98	3	85	Bacteroidota; Ignavibacteria; Ignavibacteriales; Ignavibacteriaceae; BMS3ABIN03 sp.
**ALJR32**	GCA_022340745.1	3.9	50.4	97	1	92	Desulfobacterota; Syntrophobacteria; BM002; BM002; BM002 sp.
**ALJR35**	GCA_022340665.1	3.9	61.7	100	2	91	Acidobacteriota; Thermoanaerobaculia; Thermoanaerobaculales; Sulfomarinibacteraceae; Sulfomarinibacter sp.
**ALJR36**	GCA_022340685.1	2.2	54.8	90	2	81	Pseudomonadota; Gammaproteobacteria; UBA6429; UBA6429; JAJDNN01 sp.
**ALJR4**	GCA_022340615.1	2.3	57.4	98	0	98	Pseudomonadota; Gammaproteobacteria; Burkholderiales; Gallionellaceae; Gallionella sp.
**ALSA113**	GCA_022340645.1	3.4	47.1	97	2	88	Pseudomonadota; Gammaproteobacteria; SZUA-229; SZUA-229; GCA-2746365 sp.
**ALSA124**	GCA_022340605.1	4.8	68.2	90	0	90	Pseudomonadota; Gammaproteobacteria; Chromatiales; Chromatiaceae; Thiohalocapsa sp.
**ALSA142**	GCA_022340565.1	3.3	57.3	93	0	93	Desulfobacterota; Desulfobulbia; Desulfobulbales; Desulfocapsaceae; JABDQA01 sp.
**ALSA143**	GCA_022340585.1	6.3	70.2	100	2	91	Acidobacteriota; Vicinamibacteria; Fen-336; Fen-336; JAJDNS01 sp.
**ALSA159**	GCA_022340515.1	3.8	49.1	90	0	89	Desulfobacterota; Syntrophobacteria; BM002; BM002; BM002 sp.
**ALSA174**	GCA_022340505.1	4.2	35.2	95	2	86	Bacteroidota; Ignavibacteria; Ignavibacteriales; Ignavibacteriaceae; BMS3ABIN03 sp.
**ALSA176**	GCA_022340545.1	5.0	69.5	96	2	88	Acidobacteriota; Vicinamibacteria; Fen-336; Fen-336; JAJDNS01 sp.
**ALSA183**	GCA_022340465.1	4.4	55.2	90	2	81	Desulfobacterota; Desulfobacteria; Desulfobacterales; B30-G6; JAJDNW01 sp.
**ALSA191**	GCA_022340485.1	2.8	56.0	95	0	95	Pseudomonadota; Gammaproteobacteria; UBA9214; UBA9214; UBA9214 sp.
**ALSA234**	GCA_022340425.1	4.0	63.3	91	0	91	Desulfobacterota; Desulfobacteria; Desulfobacterales; JAABRJ01; JAABRJ01 sp.
**ALSA27**	GCA_022340445.1	3.2	65.6	98	0	98	Pseudomonadota; Gammaproteobacteria; Chromatiales; Chromatiaceae; Thiocapsa sp.
**ALSA42**	GCA_022340405.1	4.0	59.8	90	0	89	Desulfobacterota; Desulfobacteria; Desulfobacterales; JAJDOA01; JAJDOA01 sp.
**MASA1**	GCA_022340385.1	3.9	63.5	90	1	86	Pseudomonadota; Alphaproteobacteria; Rhodobacterales; Rhodobacteraceae; Rhodobacter_B sp.
**MASA10**	GCA_022340355.1	2.3	55.0	90	2	81	Pseudomonadota; Gammaproteobacteria; UBA6429; UBA6429; JAJDNN01 sp.
**MASA3**	GCA_022340315.1	3.3	63.3	90	2	81	Pseudomonadota; Alphaproteobacteria; Rhodobacterales; Rhodobacteraceae; JAJDOD01 sp.
**MASA4**	GCA_022340345.1	2.8	64.3	95	1	92	Pseudomonadota; Gammaproteobacteria; Arenicellales; HyVt-429; JAJDOE01 sp.
**MASA5**	GCA_022340305.1	4.3	56.1	90	4	68	Pseudomonadota; Gammaproteobacteria; GCA-001735895; GCA-001735895; GCA-001735895 sp.
**MASA6**	GCA_022340285.1	4.4	65.9	90	3	77	Pseudomonadota; Gammaproteobacteria; Burkholderiales; SG8-39; JAABRB01 sp.
**MASP1**	GCA_022340265.1	5.0	50.6	93	2	85	Desulfobacterota; DSM-4660; Desulfatiglandales; Desulfatiglandaceae; NaphS2 sp.
**MASP12**	GCA_022340245.1	3.4	61.4	90	1	87	Pseudomonadota; Gammaproteobacteria; DSM-19610; DSM-19610; Thiogranum sp.
**MASP15**	GCA_022340215.1	3.9	61.5	100	0	100	Pseudomonadota; Gammaproteobacteria; JAJDOJ01; JAJDOJ01; JAJDOJ01 sp.
**MASP16**	GCA_022340185.1	3.4	61.8	90	0	90	Pseudomonadota; Alphaproteobacteria; Rhizobiales; Rhizobiaceae; BOKV01 sp.
**MASP6**	GCA_022340195.1	4.4	61.2	100	0	100	Pseudomonadota; Gammaproteobacteria; Chromatiales; Chromatiaceae; Thiosymbion sp.

Cp% and Ct % correspond to the percentage of completeness and contamination calculated with CheckM.
QC is defined as (completeness %) -(5* contamination)

Table S4. Metabolic potential prediction of proteins DsrAB (dissimilatory sulfite reductase).

MAG	protein	enzyme_type
**ALJR12**	*DsrA_SRO*	reductive
**ALJR12**	*DsrB_SDM*	reductive_dismutation
**ALJR14**	*DsrB_SRO*	reductive
**ALJR14**	*DsrA_SRO*	reductive
**ALJR15**	*DsrB_SDM*	reductive_dismutation
**ALJR15**	*DsrA_SRO*	reductive
**ALJR24**	*DsrB_SRO*	reductive
**ALJR24**	*DsrA_SRO*	reductive
**ALJR25**	*DsrB_SRO*	reductive
**ALJR25**	*DsrA_SRO*	reductive
**ALJR30**	*DsrA_SRO*	reductive
**ALJR30**	*DsrB_SRO*	reductive
**ALJR32**	*DsrB_SRO*	reductive
**ALJR32**	*DsrA_SRO*	reductive
**ALJR35**	*DsrB_SRO*	reductive
**ALJR35**	*DsrA_SRO*	reductive
**ALSA142**	*DsrB_SDM*	reductive_dismutation
**ALSA142**	*DsrA_SDM*	reductive_dismutation
**ALSA143**	*DsrA_SRO*	reductive
**ALSA143**	*DsrB_SRO*	reductive
**ALSA159**	*DsrA_SRO*	reductive
**ALSA159**	*DsrB_SRO*	reductive
**ALSA174**	*DsrB_SRO*	reductive
**ALSA174**	*DsrA_SRO*	reductive
**ALSA176**	*DsrA_SRO*	reductive
**ALSA176**	*DsrB_SRO*	reductive
**ALSA183**	*DsrB_SDM*	reductive_dismutation
**ALSA183**	*DsrA_SRO*	reductive
**ALSA234**	*DsrB_SRO*	reductive
**ALSA234**	*DsrA_SRO*	reductive
**ALSA42**	*DsrB_SDM*	reductive_dismutation
**ALSA42**	*DsrA_SRO*	reductive
**MASP1**	*DsrA_SRO*	reductive
**MASP1**	*DsrB_SRO*	reductive

MAG	protein	enzyme_type
**ALJR11**	*DsrB_SOB*	oxidative
**ALJR11**	*DsrA_SOB*	oxidative
**ALJR17**	*DsrB_SOB*	oxidative
**ALJR17**	*DsrA_SOB*	oxidative
**ALJR17**	*DsrB_SOB*	oxidative
**ALJR17**	*DsrA_SOB*	oxidative
**ALJR18**	*DsrB_SOB*	oxidative
**ALJR18**	*DsrA_SOB*	oxidative
**ALJR2**	*DsrB_SOB*	oxidative
**ALJR2**	*DsrA_SOB*	oxidative
**ALJR28**	*DsrB_SOB*	oxidative
**ALJR28**	*DsrA_SOB*	oxidative
**ALJR36**	*DsrA_SOB*	oxidative
**ALJR36**	*DsrB_SOB*	oxidative
**ALJR4**	*DsrA_SOB*	oxidative
**ALJR4**	*DsrB_SOB*	oxidative
**ALSA113**	*DsrA_SOB*	oxidative
**ALSA113**	*DsrB_SOB*	oxidative
**ALSA124**	*DsrA_SOB*	oxidative
**ALSA124**	*DsrB_SOB*	oxidative
**ALSA191**	*DsrA_SOB*	oxidative
**ALSA191**	*DsrB_SOB*	oxidative
**ALSA27**	*DsrB_SOB*	oxidative
**ALSA27**	*DsrA_SOB*	oxidative
**MASA10**	*DsrA_SOB*	oxidative
**MASA10**	*DsrB_SOB*	oxidative
**MASA4**	*DsrA_SOB*	oxidative
**MASA4**	*DsrB_SOB*	oxidative
**MASA5**	*DsrB_SOB*	oxidative
**MASA5**	*DsrA_SOB*	oxidative
**MASP12**	*DsrB_SOB*	oxidative
**MASP12**	*DsrA_SOB*	oxidative
**MASP15**	*DsrA_SOB*	oxidative
**MASP15**	*DsrB_SOB*	oxidative
**MASP15**	*DsrA_SOB*	oxidative
**MASP6**	*DsrB_SOB*	oxidative
**MASP15**	*DsrB_SOB*	oxidative
**MASP6**	*DsrA_SOB*	oxidative

Table S5. Number of reads recruited per MAG after Bowtie2 metagenomic sample alignment.

Site	Sample	**QC Reads**	ALSA 124	ALSA 174	ALJR 36	ALJR 15	MASA 10	MAS A4	MASP 15	MASP 16
ALJR	SRR11061153	**66,168,546**	91,688	25,241	56,844	205,401	55,677	2,340	14,527	6,510
ALJR	SRR11061154	**40,593,738**	53,120	17,621	37,973	137,232	37,214	1,549	9,459	4,516
ALJR	SRR11567260	**90,031,614**	195,351	107,487	173,642	104,455	169,858	1,959	12,945	8,576
ALJR	SRR11567261	**41,031,419**	88,205	49,593	80,280	47,615	79,093	906	5,938	4,136
ALJR	SRR9045291	**83,691,004**	85,564	69,877	106,631	384,306	104,804	2,516	13,924	20,028
ALJR	SRR9045294	**107,792,129**	560,620	103,103	134,973	151,345	132,545	2,644	27,358	6,436
ALJR	SRR9045295	**59,159,686**	194,273	78,062	123,209	320,041	120,770	2,031	12,806	9,257
ALJR	SRR9045297	**89,533,791**	235,235	125,168	127,352	95,328	124,740	2,099	16,693	10,299
	Average	*72,250,240*	*188,007.0*	*72,019.0*	*105,113.0*	*180,715.4*	*103,087.6*	*2,005.5*	*142,06.3*	*8,719.8*
ALSA	SRR10854653	**46,496,251**	77,199	117,876	70,538	28,116	69,321	937	3,702	3,071
ALSA	SRR11567157	**116,508,305**	628,802	101,396	299,068	58,002	294,029	2,854	17,103	8,821
ALSA	SRR9045292	**142,491,993**	948,105	51,523	69,618	92,285	68,649	4,537	22,458	8,663
ALSA	SRR9045293	**94,441,225**	415,145	96,176	174,055	43,758	171,089	2,054	9,960	4,508
ALSA	SRR9045296	**58,817,185**	212,625	56,378	209,538	44,498	205,944	1,368	8,981	3,630
ALSA	SRR9045298	**55,527,119**	276,908	46,031	107,904	76,680	106,493	1,665	8,923	2,891
	Average	*85,713,679*	*426,464.0*	*78,230.0*	*155,120.2*	*57,223.2*	*152,587.5*	*2,235.8*	*11,854.5*	*5,264.0*
MASA	SRR11828800	**47,120,515**	4,184	2,614	47,096	3,769	50,612	42,444	4,487	5,837
MASA	SRR11828899	**43,938,191**	15,191	1,767	21,895	4,840	23,771	16,869	7,222	3,897
MASA	SRR11829000	**42,331,083**	4,036	2,276	35,056	4,237	38,172	24,673	3,785	3,821
MASA	SRR11829104	**46,675,383**	3,841	1,896	30,439	10,508	33,171	94,327	4,918	3,713
MASA	SRR11829269	**50,747,510**	2,166	1,364	26,735	2,512	29,769	51,265	3,962	4,869
	Average	*46,162,536*	*5,883.6*	*1,983.4*	*32,244.2*	*5,173.2*	*35,099.0*	*45,915.6*	*4,874.8*	*4,427.4*
MASP	SRR11828593	**31,923,829**	819	641	2,503	6,426	2,653	4,752	26,749	10,550
MASP	SRR11828889	**40,039,222**	1,813	221	4,811	5,610	5,043	2,788	39,275	22,421
MASP	SRR11829102	**36,950,534**	1,794	443	12,424	15,484	12,910	2,481	30,723	20,022
MASP	SRR11829268	**40,622,394**	2,283	356	1,507	268,190	1,625	3,028	9,608	6,983
MASP	SRR12659820	**58,336,509**	9,438	3,301	31,743	26,305	33,274	8,328	363,112	127,955
	Average	*41,574,497*	*3,229.4*	*992.4*	*10,597.6*	*64,403.0*	*11,101.0*	*4,275.4*	*93,893.4*	*37,586.2*

Table S6. Estimation of completeness (CheckM) of the primary MAGs and the reference-guided reassembled MAGs.[1]

Site	Sample	ALSA 124	ALSA 174	ALJR 36	ALJR 15	MASA 10	MASA 4	MASP 15	MASP 16
	**Primary MAG**	**95.59**	**92.18**	**90.37**	**92.74**	**91.77**	**96.21**	**96.41**	**94.58**
ALJR	SRR11061153	87.28	79.61	91.59	93.55	88.82	64.21	57.41	57.98
ALJR	SRR11061154	82.39	48.32	86.98	92.88	84.65	58.82	55.1	55.54
ALJR	SRR11567260	86.44	93.3	93.53	90.3	92.79	64.05	66.16	78.37
ALJR	SRR11567261	79.08	88.69	91.79	71.91	90.46	54.13	46.02	66.03
ALJR	SRR9045291	90.46	15.01	85.29	-	92.16	73.74	68.67	83.54
ALJR	SRR9045294	96.53	93.58	87.88	90.61	91.08	73.69	77.24	82.29
ALJR	SRR9045295	93.31	94.97	92.57	57.92	91.3	67.14	76.72	81.71
ALJR	SRR9045297	90.2	89.66	93.01	-	92.75	81.27	65.05	57.98
ALSA	SRR10854653	89.83	92.72	-	48.89	90.57	59.3	50.44	57.87
ALSA	SRR11567157	93.21	94.69	88.04	81.23	93.47	78.63	75.86	57.98
ALSA	SRR9045292	98.12	91.76	87.88	89.61	88.36	77.25	91.38	79.7
ALSA	SRR9045293	94.25	91.2	92.74	0	92.82	71.26	66.67	57.98
ALSA	SRR9045296	90.75	91.9	93.28	-	92.71	54.37	61.57	60.55
ALSA	SRR9045298	88.58	94.67	92.32	-	91.28	70.36	73.98	57.98
MASA	SRR11828800	72.68	4.23	86.44	0	90.53	90.84	42.44	65.44
MASA	SRR11828899	66.38	0	74.25	5.49	83.82	82.29	44.11	65.27
MASA	SRR11829000	41.61	0	83.18	5.26	88.2	85.91	27.87	51.75
MASA	SRR11829104	58.09	2.08	81.7	10.69	87.97	95.29	38.71	61.99
MASA	SRR11829269	60.78	2.82	80.8	10.85	86.82	86.28	41.43	70.95
MASP	SRR11828593	30.17	-	-	3.51	15.67	31.33	86.23	67.26
MASP	SRR11828889	24.66	-	19.12	0	27.87	26.78	92.39	83.92
MASP	SRR11829102	35.59	-	55.95	0	75.24	32.47	84.92	84.19
MASP	SRR11829268	44.73	3.61	8.03	70.74	11.19	33.58	48.5	48.6
MASP	SRR12659820	82.39	2.59	81.81	6.4	89.36	69.73	96.93	89.52

[1] Empty cells (-) indicate reference-guided reassembly efforts that failed to produce any contigs/bins.

Table S7. Results of the functional enrichment analysis comparing the metabolic functionalities of ALJR36 and MASA10 pangenomes at COG20 Category level. Each pangenome group is represented by the primary MAG and the site-specific reassembled MAGs.

COG20 CATEGORY	Adjusted q value	COG ID
**Categories significantly enriched in the ALJR36 pangenome group.**		
Carbohydrate transport and metabolism-Signal transduction mechanisms	3.69E-08	G-T
Cell motility-Carbohydrate transport and metabolism	3.69E-08	N-G
Coenzyme transport and metabolism-Transcription	3.69E-08	H-K
Energy production and conversion-General function prediction only	1.45E-07	C-R
Intracellular trafficking, secretion, and vesicular transport-Intracellular trafficking, secretion, and vesicular transport	1.45E-07	U-U
Amino acid transport and metabolism-General function prediction only-Replication, recombination and repair	1.45E-07	E-R-L
Amino acid transport and metabolism-Posttranslational modification, protein turnover, chaperones	3.21E-06	E-O
Coenzyme transport and metabolism-Inorganic ion transport and metabolism	1.13E-05	H-P
Transcription-General function prediction only	1.13E-05	K-R
Extracellular structures	3.89E-05	W
Energy production and conversion-Amino acid transport and metabolism	0	C-E
**Categories significantly enriched in the MASA10 pangenome group.**		
Cell wall/membrane/envelope biogenesis-Intracellular trafficking, secretion, and vesicular transport	3.69E-08	M-U
Signal transduction mechanisms-Intracellular trafficking, secretion, and vesicular transport-Extracellular structures	3.69E-08	T-U-W
General function prediction only-Inorganic ion transport and metabolism	1.45E-07	R-P
Cell motility-Intracellular trafficking, secretion, and vesicular transport	7.50E-07	N-U
Defense mechanisms-Lipid transport and metabolism	3.21E-06	V-I
Cell wall/membrane/envelope biogenesis-Coenzyme transport and metabolism-General function prediction only	1.13E-05	M-H-R
Coenzyme transport and metabolism-Nucleotide transport and metabolism	1.62E-05	H-F
Inorganic ion transport and metabolism-Coenzyme transport and metabolism	0.05	P-H

Table S8. Results of the functional enrichment analysis comparing the metabolic functionalities of ALJR36 and MASA10 pangenomes at the KEGG Module level. Each pangenome group includes the primary MAG and the site-specific reassembled MAGs.

KEGG Module	Adjusted q value	KEGG ID
**Modules significantly enriched in the ALJR36 pangenome group.**		
Glycolysis (Embden-Meyerhof pathway), glucose => pyruvate-Glycolysis, core module involving three-carbon compounds-Gluconeogenesis, oxaloacetate => fructose-6P-Semi-phosphorylative Entner-Doudoroff pathway, gluconate => glycerate-3P-D-galactonate degradation, De Ley-Doudoroff pathway, D-galactonate => glycerate-3P-Reductive pentose phosphate cycle (Calvin cycle)-Reductive pentose phosphate cycle, ribulose-5P => glyceraldehyde-3P-Oxygenic photosynthesis in plants and cyanobacteria-Anoxygenic photosynthesis in purple bacteria	3.92E-08	M00001-M00002-M00003-M00308-M00552-M00165-M00166-M00611-M00612
Pentose phosphate pathway (Pentose phosphate cycle)-Pentose phosphate pathway, non-oxidative phase, fructose 6P => ribose 5P-Reductive pentose phosphate cycle (Calvin cycle)-Reductive pentose phosphate cycle, glyceraldehyde-3P => ribulose-5P-Oxygenic photosynthesis in plants and cyanobacteria-Anoxygenic photosynthesis in purple bacteria	3.92E-08	M00004-M00007-M00165-M00167-M00611-M00612
Inosine monophosphate biosynthesis, PRPP + glutamine => IMP-Adenine ribonucleotide biosynthesis, IMP => ADP,ATP	1.73E-07	M00048-M00049
Phosphate acetyltransferase-acetate kinase pathway, acetyl-CoA => acetate-Methanogenesis, acetate => methane-Methanogen-Acetogen	8.13E-07	M00579-M00357-M00617-M00618
GABA (gamma-Aminobutyrate) shunt	6.40E-05	M00027
Citrate cycle (TCA cycle, Krebs cycle)-Citrate cycle, second carbon oxidation, 2-oxoglutarate => oxaloacetate-Reductive citrate cycle (Arnon-Buchanan cycle)-Succinate dehydrogenase, prokaryotes-Anoxygenic photosynthesis in green nonsulfur bacteria-Anoxygenic photosynthesis in green sulfur bacteria	9.23E-05	M00009-M00011-M00173-M00149-M00613-M00614
Isoleucine biosynthesis, pyruvate => 2-oxobutanoate-Leucine biosynthesis, 2-oxoisovalerate => 2-oxoisocaproate	0.0015	M00535-M00432
**Modules significantly enriched in the MASA10 pangenome group.**		
Cobalamin biosynthesis, anaerobic, uroporphyrinogen III => sirohydrochlorin => cobyrinate a,c-diamide-Cobalamin biosynthesis, aerobic, uroporphyrinogen III => precorrin 2 => cobyrinate a,c-diamide	3.92E-08	M00924-M00925
Pentose phosphate pathway (Pentose phosphate cycle)-Pentose phosphate pathway, non-oxidative phase, fructose 6P => ribose 5P-Pentose phosphate pathway, archaea, fructose 6P => ribose 5P-Reductive pentose phosphate cycle (Calvin cycle)-Reductive pentose phosphate cycle, glyceraldehyde-3P => ribulose-5P-Oxygenic photosynthesis in plants and cyanobacteria-Anoxygenic photosynthesis in purple bacteria	3.92E-08	M00004-M00007-M00580-M00165-M00167-M00611-M00612
Phosphatidylcholine (PC) biosynthesis, PE => PC	3.92E-08	M00091
Assimilatory sulfate reduction, sulfate => H2S-Dissimilatory sulfate reduction, sulfate => H2S-Sulfate-sulfur assimilation	1.73E-07	M00176-M00596-M00616
Catechol meta-cleavage, catechol => acetyl-CoA / 4-methylcatechol => propanoyl-CoA	1.73E-07	M00569
Isoleucine biosynthesis, threonine => 2-oxobutanoate => isoleucine	8.13E-07	M00570
Acylglycerol degradation	0.030088581742318	M00098

Table S9. NCBI accession numbers of unvegetated creekbed sediment samples (PRJNA814317).

sampleID	SRAid	metadata	Reads
G1_1_3	SRR24130648	Greenwood Creek, Ipswich, MA. Enriched creek (receiving input from treated sewage effluent). Increasing salinity gradient from freshwater terrestrial stream to Plum Island Sound (1-6)	5,598,089
G1_2_4	SRR24130647		7,853,810
G1_3_4_1	SRR24130636		8,772,437
G1_3_4_2	SRR24130625		6,054,309
G2_1_2	SRR24130622		7,261,066
G2_2_1	SRR24130621		8,208,631
G2_3_3	SRR24130620		9,320,183
G3_1_2	SRR24130619		6,326,147
G3_2_1	SRR24130618		5,978,731
G3_3_2	SRR24130617		6,445,823
G4_1_2	SRR24130646		9,757,468
G4_2_3	SRR24130645		9,156,045
G4_3_1	SRR24130644		11,095,313
G5_1_2	SRR24130643		5,624,988
G5_2_4	SRR24130642		7,813,299
G5_3_4	SRR24130641		5,898,050
G6_2_4	SRR24130640		8,759,716
R1_1_4	SRR24130639	Egypt Creek, Ipswich, MA. Reference creek (receiving input from drinking water reservoir). Increasing salinity gradient from freshwater terrestrial stream to Plum Island Sound (1-6)	9,179,906
R1_2_4	SRR24130638		3,536,787
R1_3_4	SRR24130637		5,327,512
R2_1_1	SRR24130635		9,563,259
R2_2_1	SRR24130634		9,559,918
R2_3_4	SRR24130633		10,067,392
R3_1_3	SRR24130632		10,325,098
R3_2_1	SRR24130631		9,799,442
R3_3_3	SRR24130630		10,554,621
R4_1_3	SRR24130629		10,067,285
R4_2_3	SRR24130628		7,681,587
R4_3_3	SRR24130627		9,884,460
R6_1_2	SRR24130626		6,638,196
R6_2_1	SRR24130624		10,642,403
R6_3_1	SRR24130623		6,145,961

Supplemental Data files description

Supplemental Data 1. DRAM raw annotations.

Supplemental Data 2. DRAM metabolism summary.

Supplemental Data 3. Contig-specific coverage values of the Alabama and Massachusetts salt marsh samples.

Supplemental Data 4. Contig-specific coverage values of the unvegetated salt marsh samples.

Supplemental Data 5. Anvio profiling (read recruitment results, SNVs, indels).

Supplemental Data 6. Genes in MASA10 and MASP16 MAGs identified as geographically restricted.

Supplemental Data 7. Reference-guided metagenome reconstruction.

Supplemental Data 8. Enrichment analyses (MASA10, ALJR36).

Supplemental Data 9. Genes selected for phylogenetic tree reconstruction and average gene entropy analysis. Results of average gene entropy analysis.

Figure S1. Maximum likelihood phylogenetic trees of the *dsrA* (top) and *dsrB* (bottom) genes identified in sulfur-cycling MAGs from Alabama and Massachusetts salt marshes. Phyla are shown on the right of each branch.

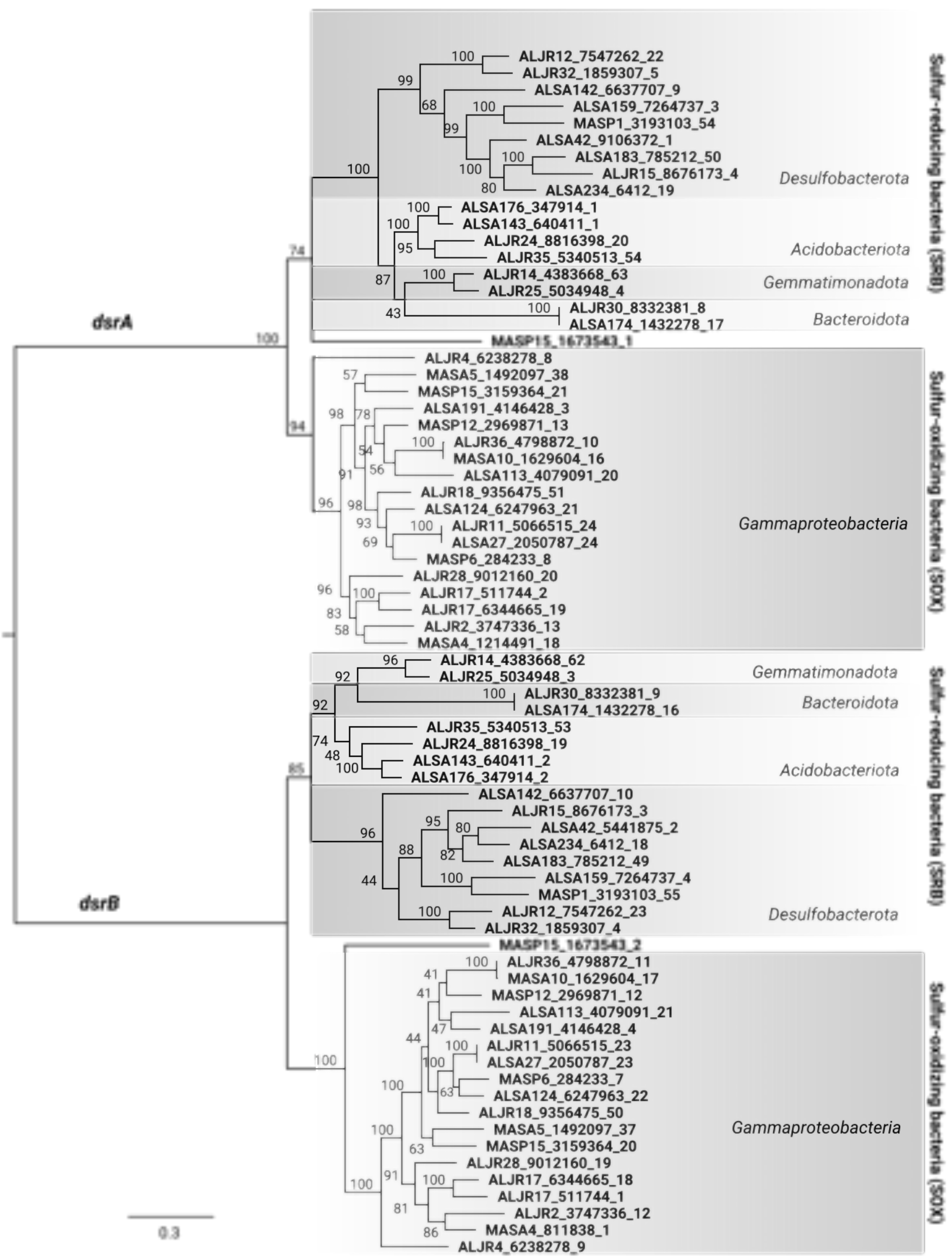

Figure S2. Primary MAGs for which read-mapping was statistically more abundant across locations and vegetation zones by pairwise statistical analysis (STAMP) using average CPM values calculated for each primary MAG. Mean proportion of reads (and pairwise differences) are shown with confidence intervals. A) Comparison by location and vegetation: Massachusetts- *S. pumilus* (MASP), Massachusetts- *S. alterniflorus* (MASA), Alabama- *S. alterniflorus* (ALSA), Alabama- *J. roemerianus* (ALJR). B) Comparison by location (Alabama vs. Massachusetts).

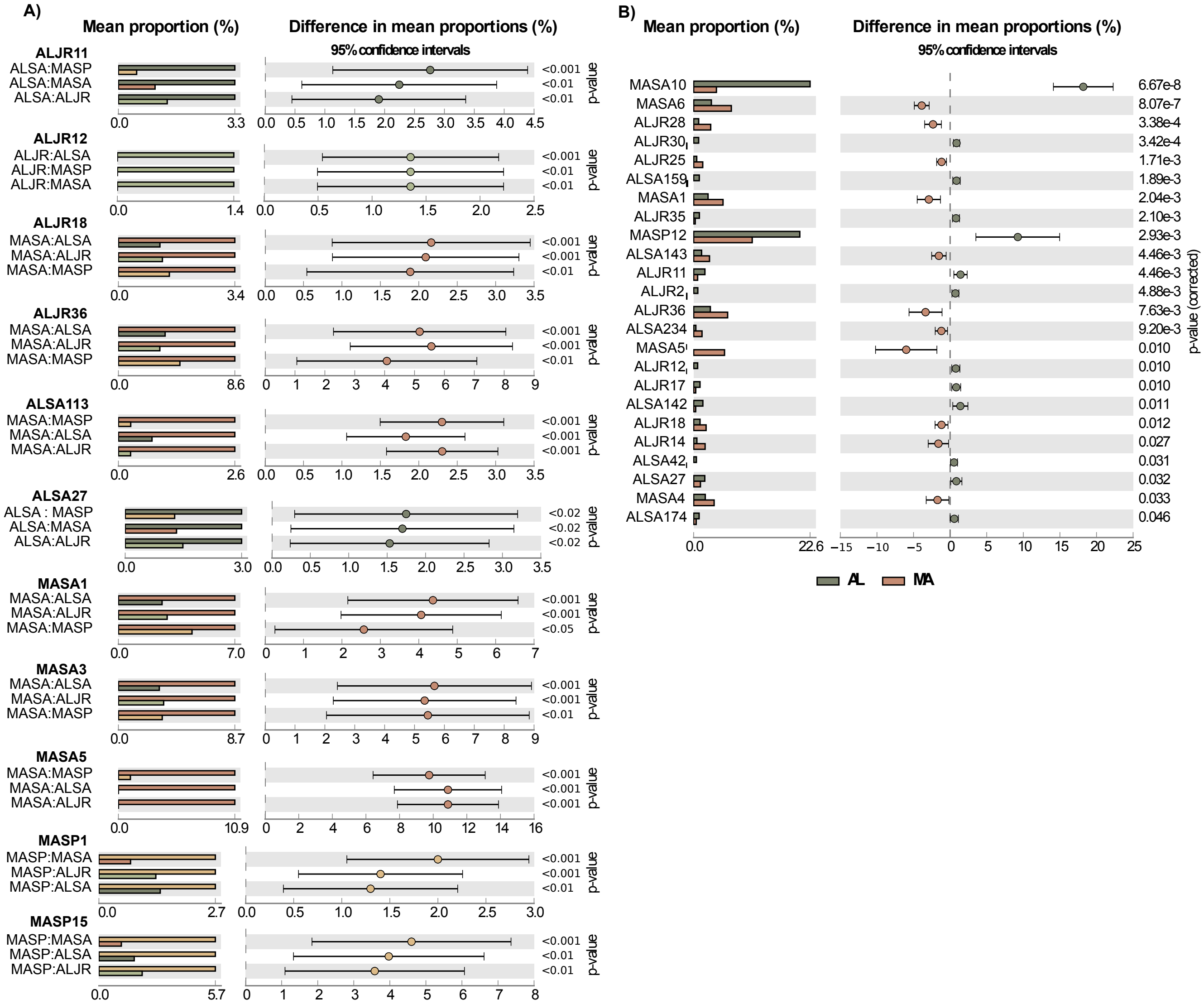

Figure S3. Distribution of sulfur cycling primary MAG sequences across environmental metagenomes.Samples included published metagenomes from subtidal sediments along a salinity gradient. Circle size reflects averaged GCPM across all contigs for each MAG. Contig-specific coverage values can be found in Supplemental Data 4 and sample description in Table S9.

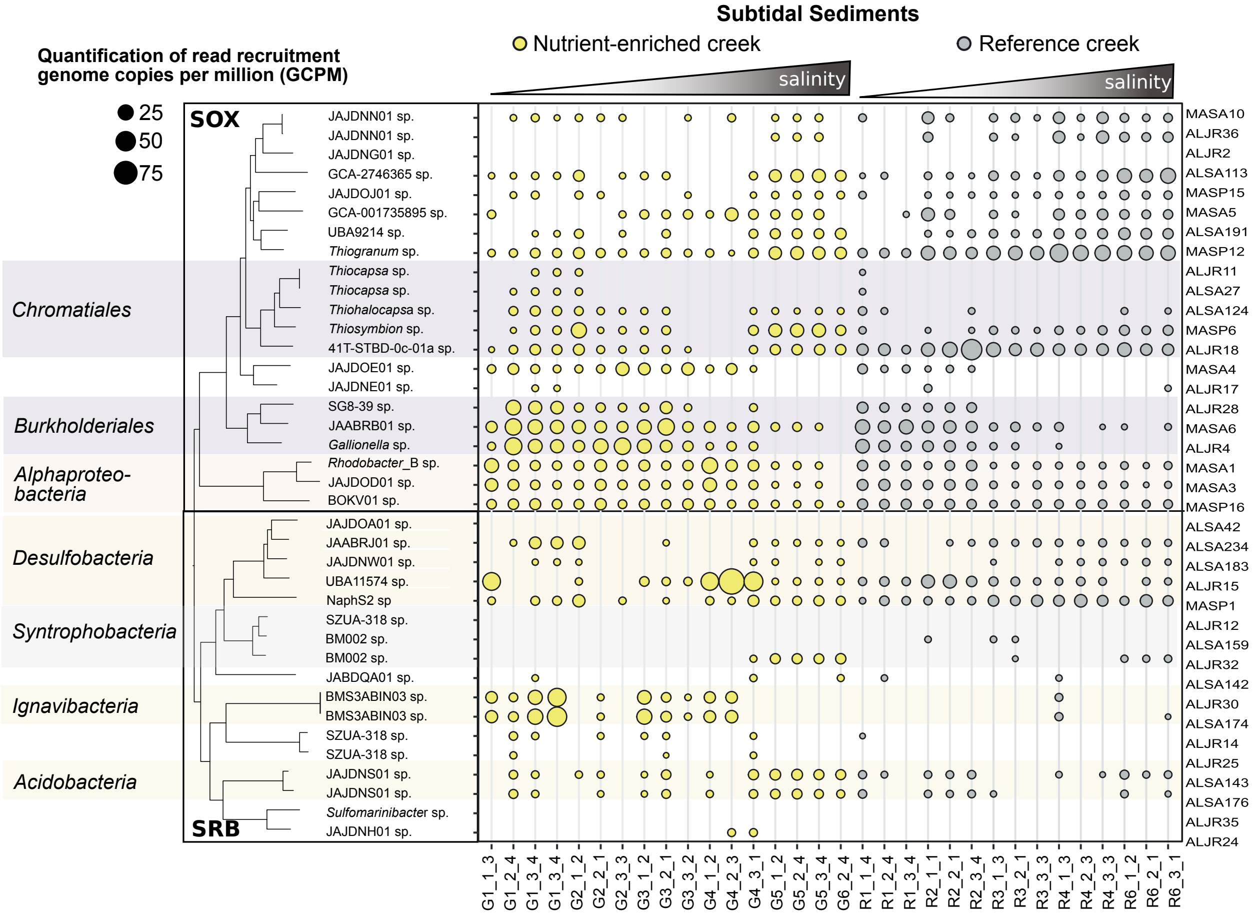

Figure S4. Contig-specific coverage values for each salt marsh sample included in this study of each sulfur cycling bacteria MAG identified in the co-assembly from Alabama in sediments inhabited by the plant *Juncus roemerianus* (ALJR). For each sample, contig coverage (expressed as CMP, y-axis) is sorted from low to high. For comparison purposes, samples collected in the same geographical area and in the proximity of the same plant are colored with the same color. Full data is presented in Supplemental Data 3.

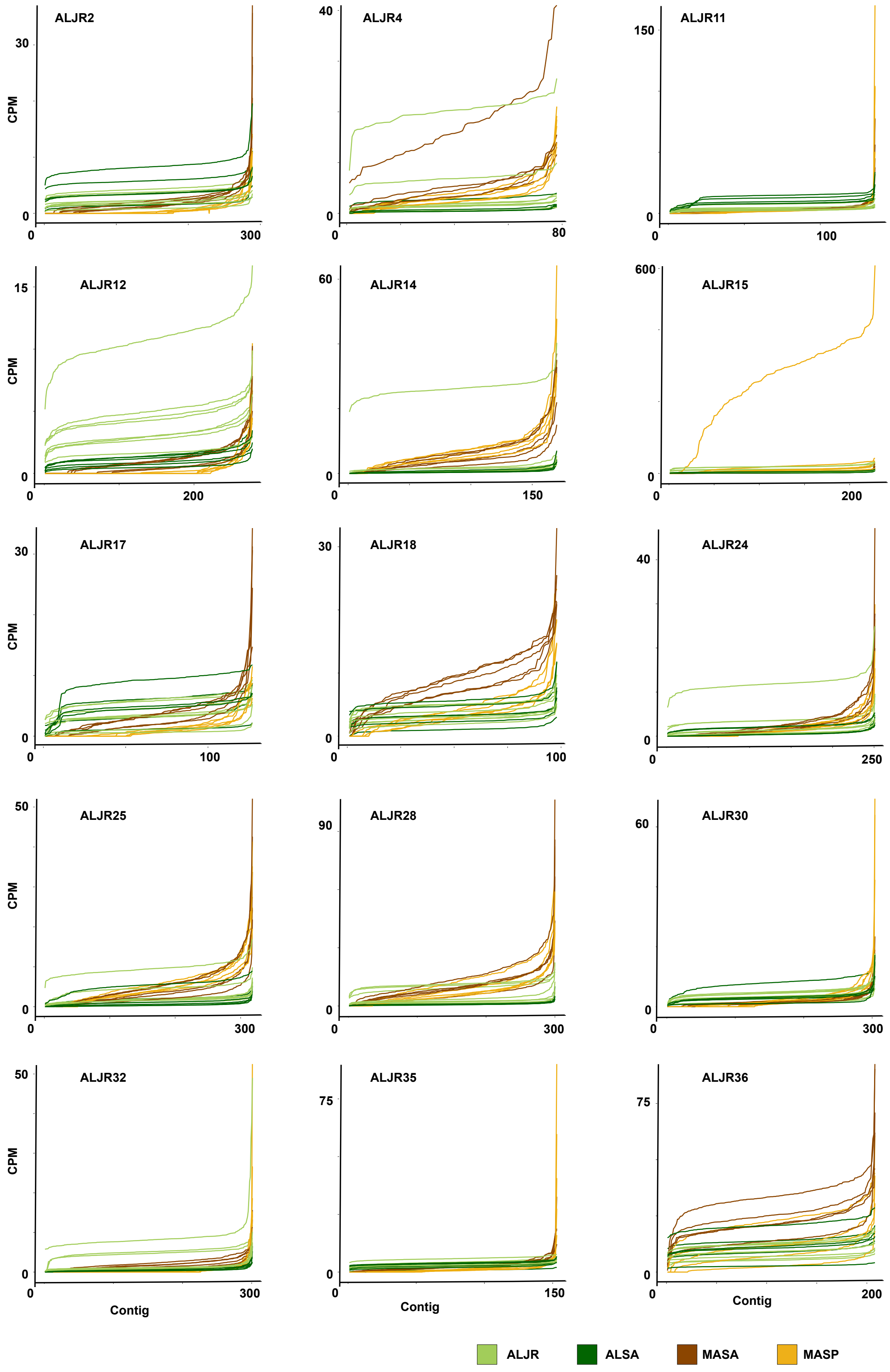

Figure S5. Contig-specific coverage values for each salt marsh sample included in this study of each sulfur cycling bacteria MAG identified in the co-assembly from Alabama in sediments inhabited by the plant *Sporobulus alterniflorus* (ALSA). For each sample, contig coverage (expressed as CMP, y-axis) is sorted from low to high. For comparison purposes, samples collected in the same geographical area and in the proximity of the same plant are colored with the same color. Full data is presented in Supplemental Data 3.

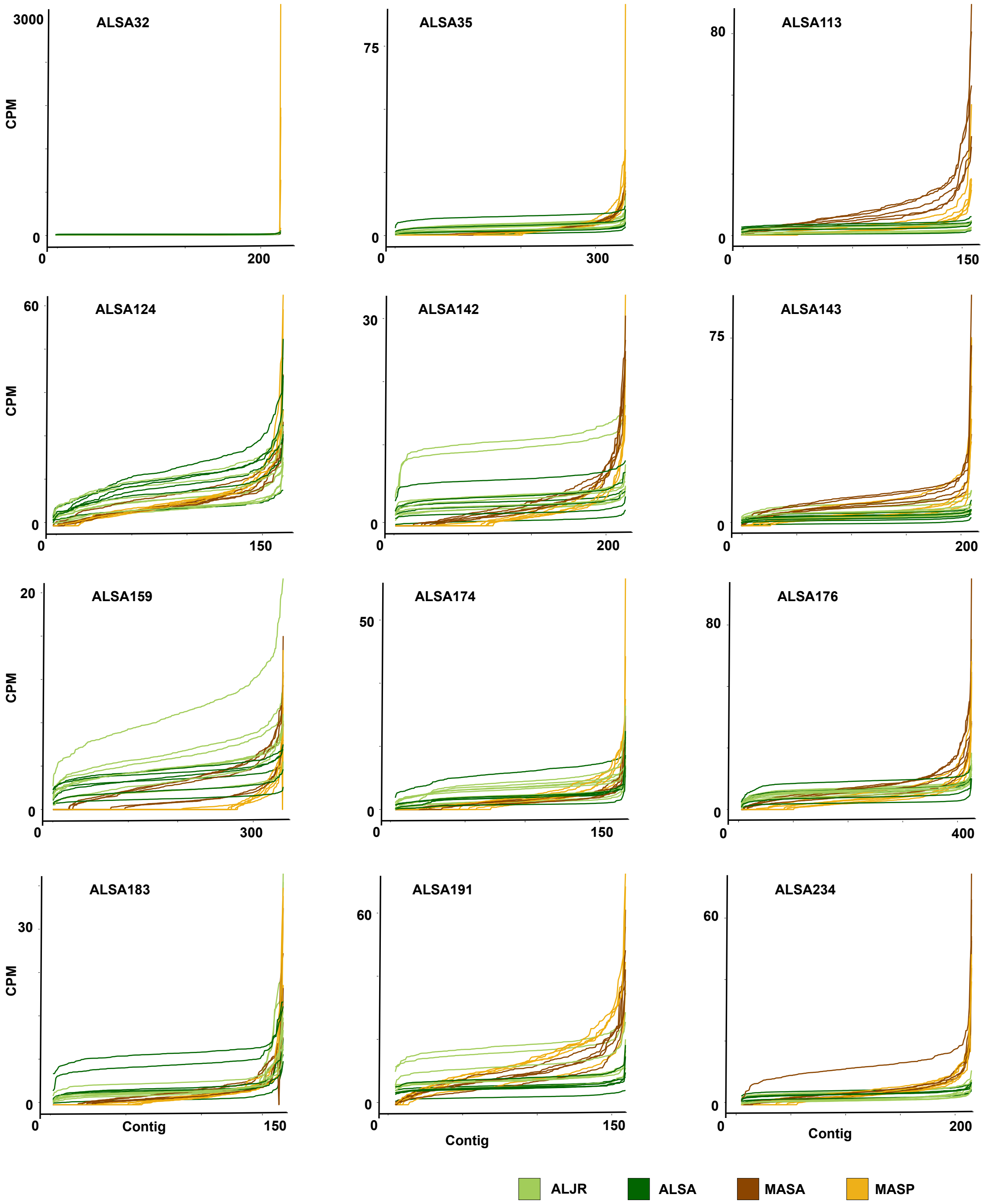

Figure S6. Contig-specific coverage values for each salt marsh sample included in this study of each sulfur cycling bacteria MAG identified in the co-assembly from Massachusetts in sediments inhabited by *Sporobolus alterniflorus* (MASA) or *Sporobolus pumilus* (MASP). For each sample, contig coverage (expressed as CMP, y-axis) is sorted from low to high. For comparison purposes, samples collected in the same geographical area and in the proximity of the same plant are colored with the same color. Full data is presented in Supplemental Data 3.

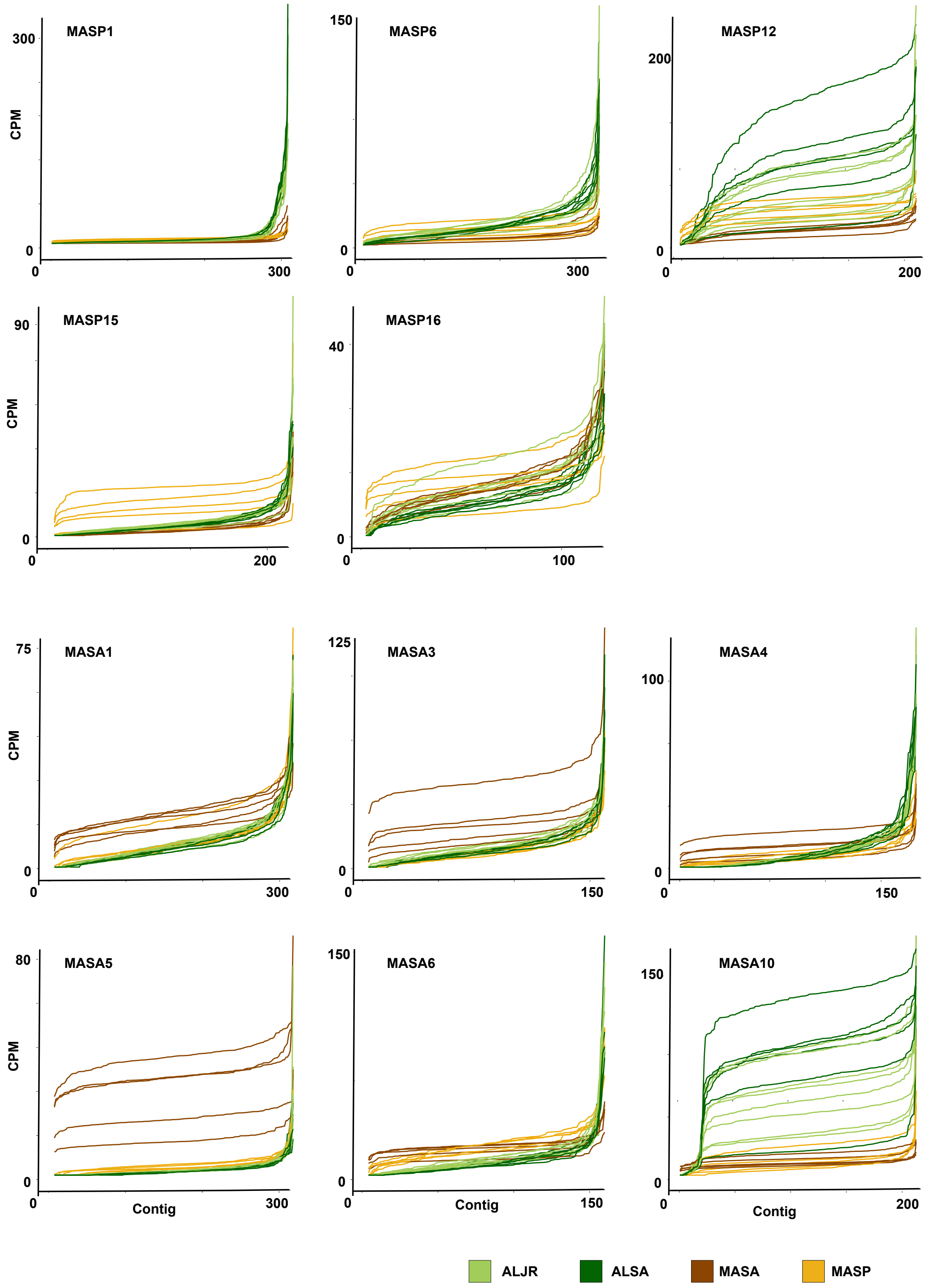

Figure S7-S14. A)Anvi'o coverage profiles of eight selected sulfur-cycling bacteria MAGs. In each anviogram, outer cycles represent the contig coverage (as mean coverage) of each of the 24 salt-marsh metagenomic samples included in this analysis. Samples are color-coded according to geographical site and the dominant plant species in the area where sediment collection occurred. Inner rings represent GC content (dark gray) and contig length (gray). Contigs are clustered (inner tree) based on the sequence composition and differential coverage using Euclidean distance and Ward hierarchical clustering method. The samples' order (rings) was determined using a clustering method based on the mean coverage. Bar plots represent (top to bottom) the total number of reads in each library, the total number of reads mapped to each respective MAG, the percentage of mapped reads, and the total number of Single Nucleotide Variants (SNV), Single Codon Variants (SCV) and indels. B) Anvi'o pangenomic analysis of eight sulfur-cycling bacteria MAGs. In each ring, each vertical line represents a gene. The genes' order was determined using Euclidean distance and Ward clustering method based on the presence or absence of a gene cluster across all MAGs. In the outer rings, we indicate the number of genes in each gene cluster, the number of paralogous, and the results of the combined homogeneity test. Dark gray bars in the next set of rings indicate a known gene function, annotated according to COG 2020. The single-copy core gene cluster, present in all reference-guided reassembled MAGs, is highlighted in purple. The primary MAG used as a reference is shown in dark teal. Each of the remaining circles represents a reference-guided reassembled MAG from each of the salt-mash metagenomic samples included in this analysis. Samples are color-coded according to geographical site and the dominant plant species in the area where sediment collection occurs.

Figure S7

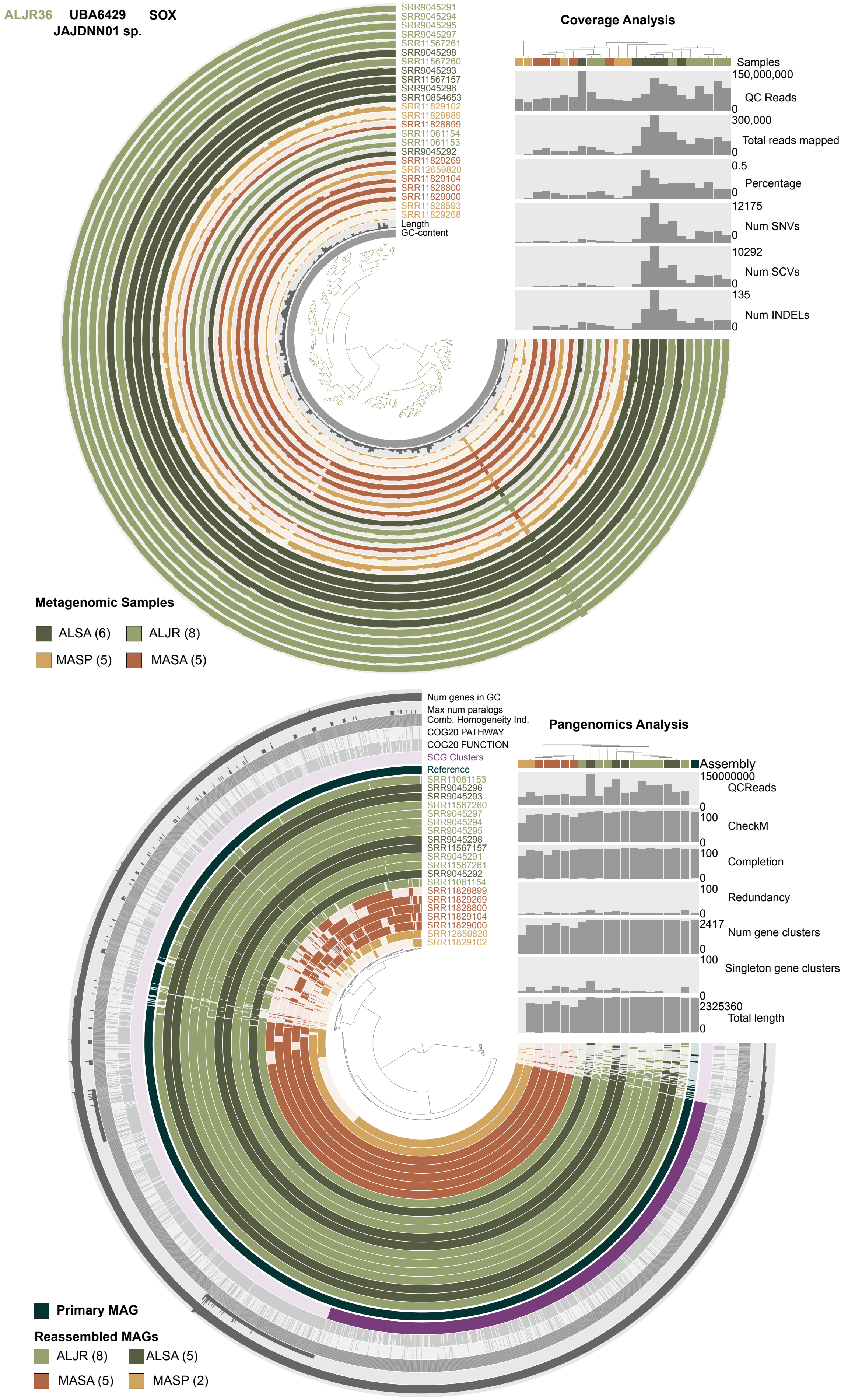

ALJR36
UBA6429
SOX
JAJDNN01 sp.
Coverage Analysis
SRR9045291
SRR9045294
SRR9045295
SRR9045297
SRR11567261
SRR9045298
SRR11567260
SRR9045293
SRR11567157
SRR9045296
SRR10854653
SRR11829102
SRR11828889
SRR11828899
SRR11061154
SRR11061153
SRR9045292
SRR11829269
SRR12659820
SRR11829104
SRR11828800
SRR11829000
SRR11828593
SRR11829268
Length
GC-content
Samples
150,000,000
QC Reads
0
300,000
Total reads mapped
0
0.5
Percentage
0
12175
Num SNVs
0
10292
Num SCVs
0
135
Num INDELs
0
Metagenomic Samples
ALSA (6)
ALJR (8)
MASP (5)
MASA (5)
Num genes in GC
Max num paralogs
Comb. Homogeneity Ind.
COG20 PATHWAY
COG20 FUNCTION
SCG Clusters
Reference
SRR11061153
SRR9045296
SRR9045293
SRR11567260
SRR9045297
SRR9045294
SRR9045295
SRR9045298
SRR11567157
SRR9045291
SRR11567261
SRR9045292
SRR11061154
SRR11828899
SRR11829269
SRR11828800
SRR11829104
SRR11829000
SRR12659820
SRR11829102
Pangenomics Analysis
Assembly
150000000
QCReads
0
100
CheckM
0
100
Completion
0
100
Redundancy
0
2417
Num gene clusters
0
100
Singleton gene clusters
0
2325360
Total length
0
Primary MAG
Reassembled MAGs
ALJR (8)
ALSA (5)
MASA (5)
MASP (2)

Figure S8

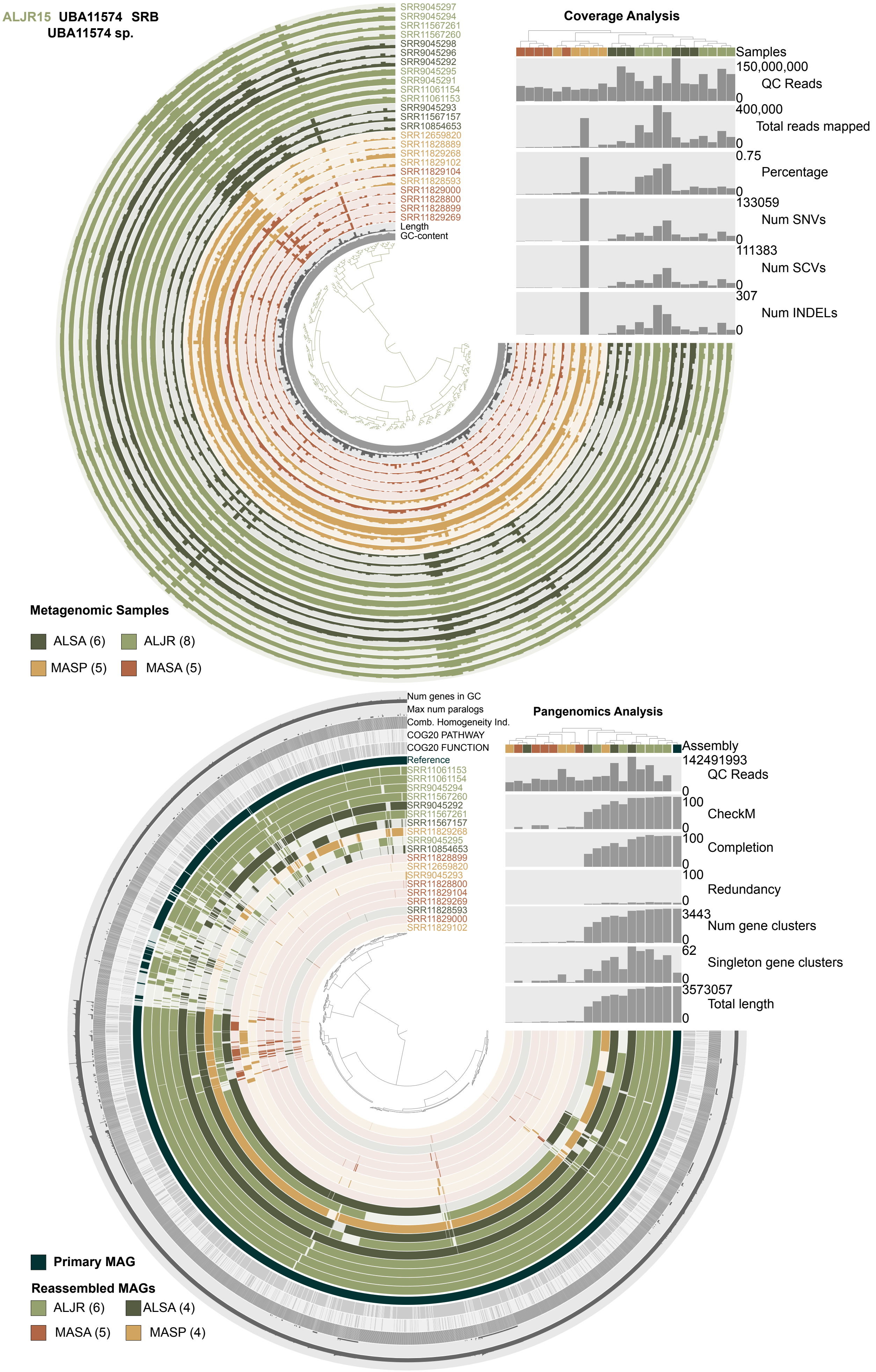

ALJR15 UBA11574 SRB
UBA11574 sp.
Coverage Analysis
SRR9045297
SRR9045294
SRR11567261
SRR11567260
SRR9045298
SRR9045296
SRR9045292
SRR9045295
SRR9045291
SRR11061154
SRR11061153
SRR9045293
SRR11567157
SRR10854653
SRR12659820
SRR11828889
SRR11829268
SRR11829102
SRR11829104
SRR11828593
SRR11829000
SRR11828800
SRR11828899
SRR11829269
Length
GC-content
Samples
150,000,000
QC Reads
0
400,000
Total reads mapped
0
0.75
Percentage
0
133059
Num SNVs
0
111383
Num SCVs
0
307
Num INDELs
0
Metagenomic Samples
ALSA (6)
ALJR (8)
MASP (5)
MASA (5)
Num genes in GC
Max num paralogs
Comb. Homogeneity Ind.
COG20 PATHWAY
COG20 FUNCTION
Reference
SRR11061153
SRR11061154
SRR9045294
SRR11567260
SRR9045292
SRR11567261
SRR11567157
SRR11829268
SRR9045295
SRR10854653
SRR11828899
SRR12659820
SRR9045293
SRR11828800
SRR11829104
SRR11829269
SRR11828593
SRR11829000
SRR11829102
Pangenomics Analysis
Assembly
142491993
QC Reads
0
100
CheckM
0
100
Completion
0
100
Redundancy
0
3443
Num gene clusters
0
62
Singleton gene clusters
0
3573057
Total length
0
Primary MAG
Reassembled MAGs
ALJR (6)
ALSA (4)
MASA (5)
MASP (4)

Figure S9

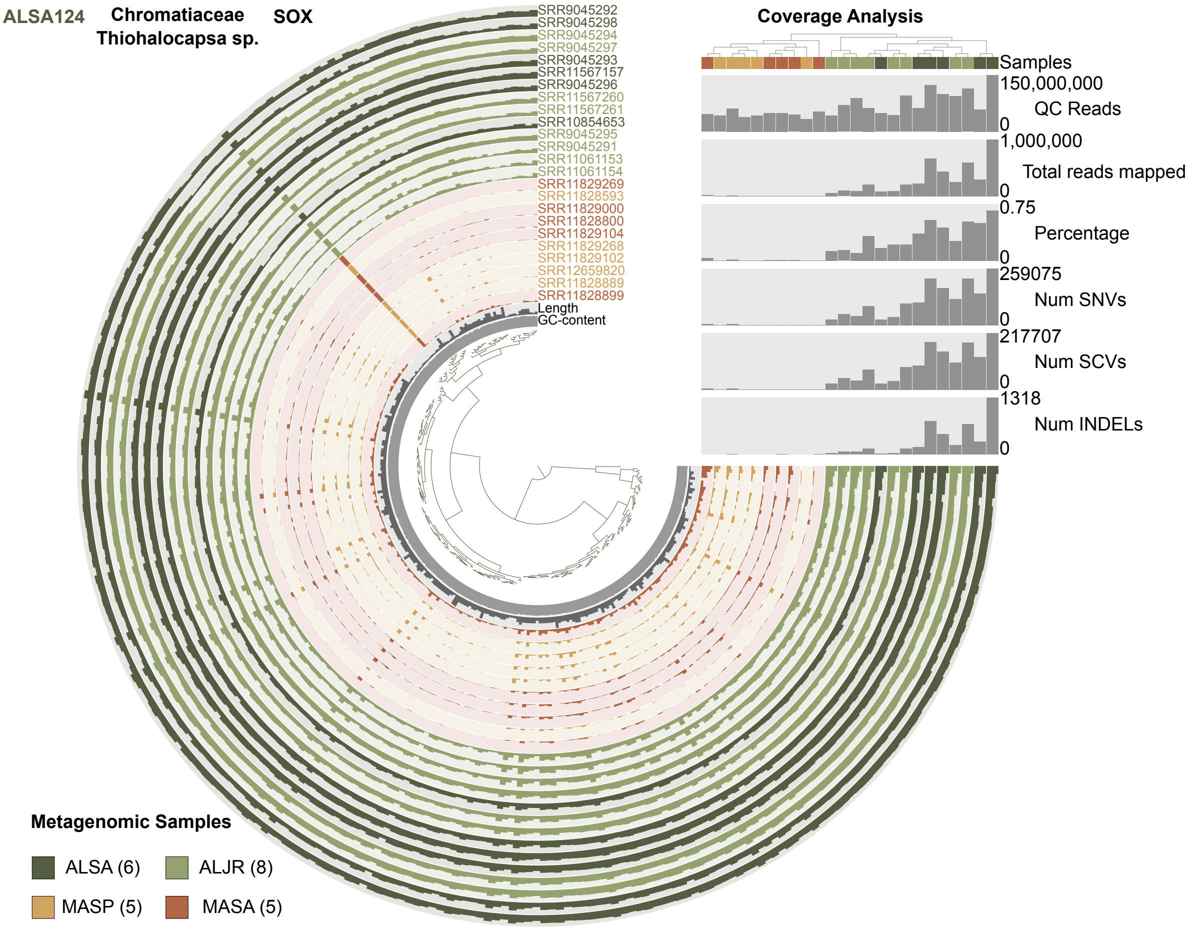

ALSA124
Chromatiaceae
Thiohalocapsa sp.
SOX
SRR9045292
SRR9045298
SRR9045294
SRR9045297
SRR9045293
SRR11567157
SRR9045296
SRR11567260
SRR11567261
SRR10854653
SRR9045295
SRR9045291
SRR11061153
SRR11061154
SRR11829269
SRR11828593
SRR11829000
SRR11828800
SRR11829104
SRR11829268
SRR11829102
SRR12659820
SRR11828889
SRR11828899
Length
GC-content
Coverage Analysis
Samples
150,000,000
QC Reads
0
1,000,000
Total reads mapped
0
0.75
Percentage
0
259075
Num SNVs
0
217707
Num SCVs
0
1318
Num INDELs
0
Metagenomic Samples
ALSA (6)
ALJR (8)
MASP (5)
MASA (5)

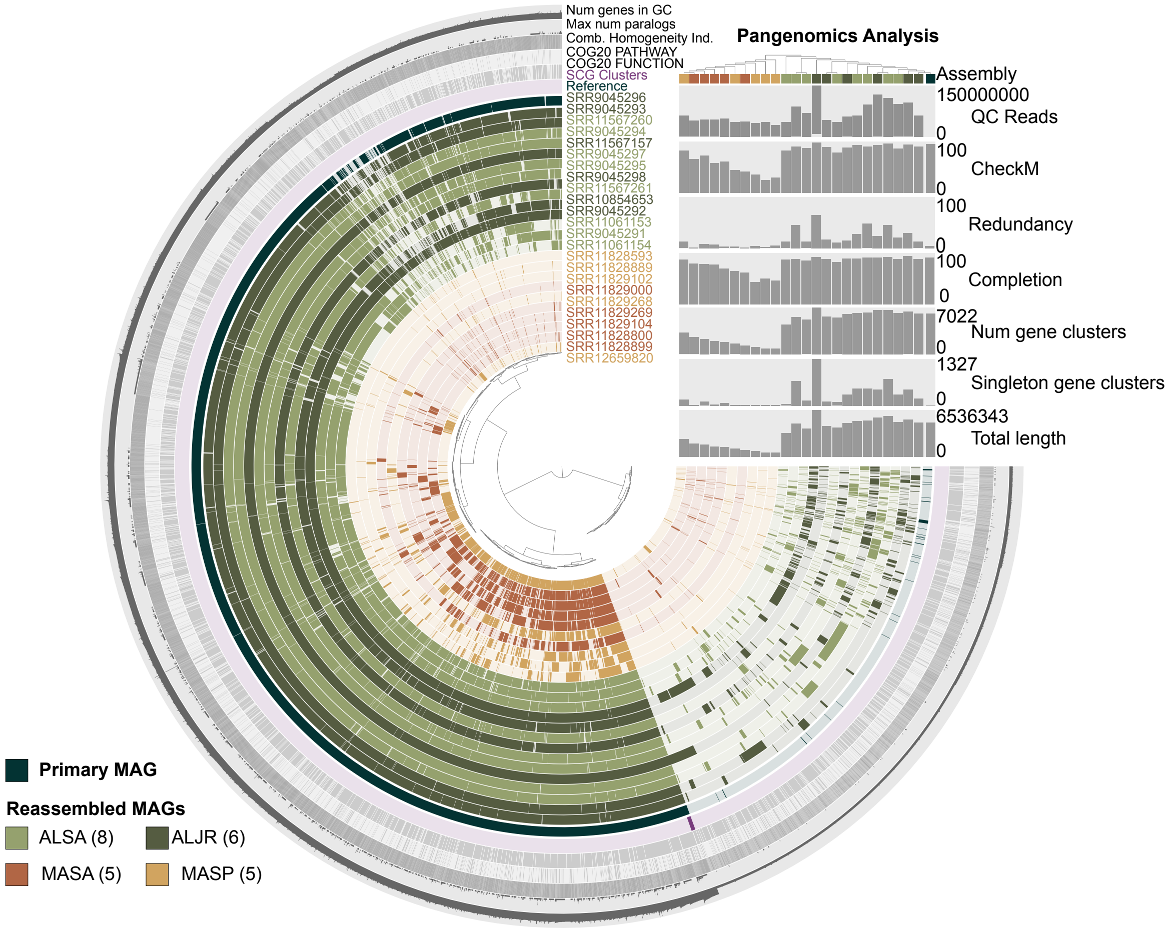

Num genes in GC
Max num paralogs
Comb. Homogeneity Ind.
COG20 PATHWAY
COG20 FUNCTION
SCG Clusters
Reference
SRR9045296
SRR9045293
SRR11567260
SRR9045294
SRR11567157
SRR9045297
SRR9045295
SRR9045298
SRR11567261
SRR10854653
SRR9045292
SRR11061153
SRR9045291
SRR11061154
SRR11828593
SRR11828889
SRR11829102
SRR11829000
SRR11829268
SRR11829269
SRR11829104
SRR11828800
SRR11828899
SRR12659820
Pangenomics Analysis
Assembly
150000000
QC Reads
0
100
CheckM
0
100
Redundancy
0
100
Completion
0
7022
Num gene clusters
0
1327
Singleton gene clusters
0
6536343
Total length
0
Primary MAG
Reassembled MAGs
ALSA (8)
ALJR (6)
MASA (5)
MASP (5)

Figure S10

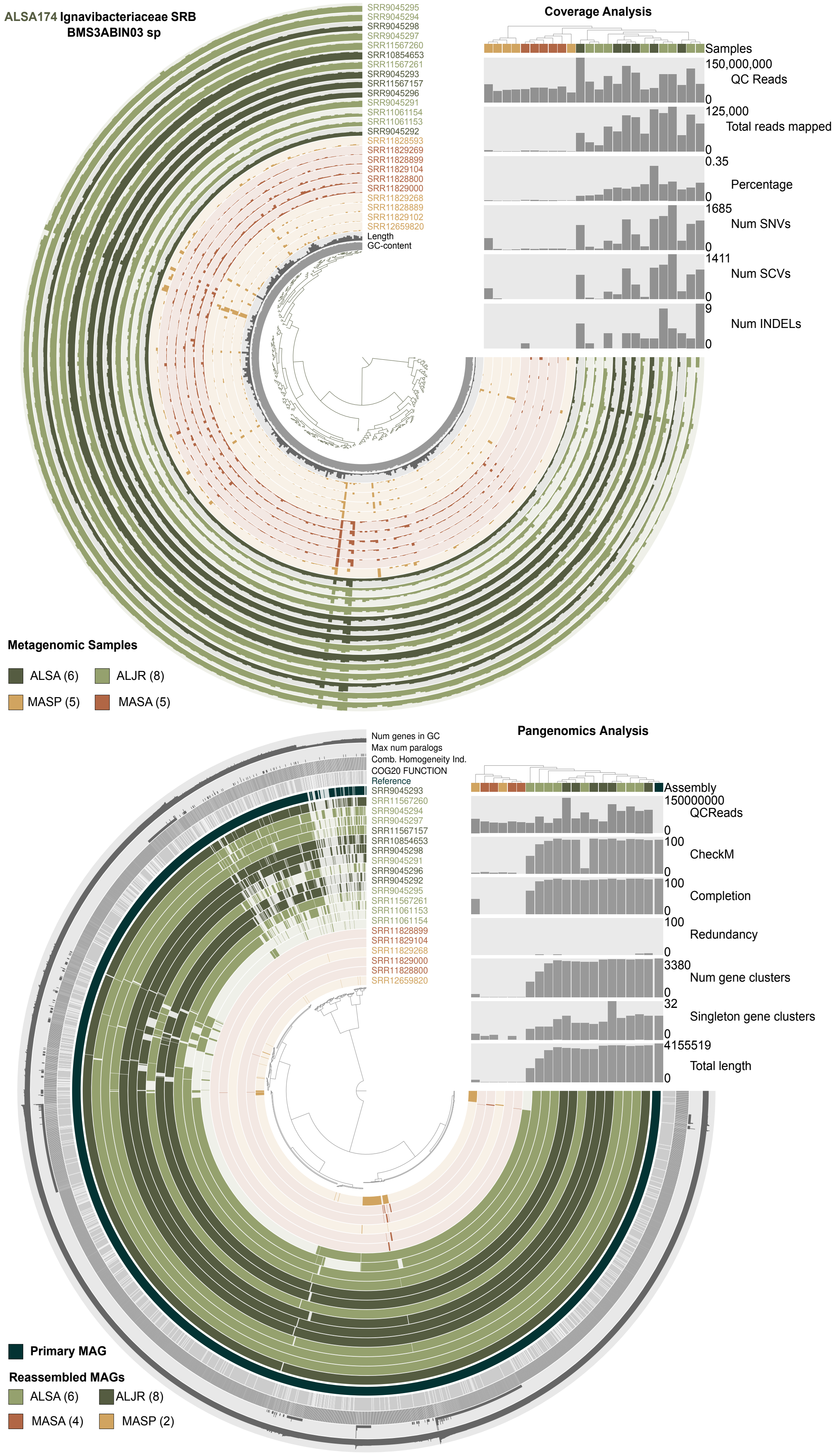

ALSA174 Ignavibacteriaceae SRB BMS3ABIN03 sp
Coverage Analysis
SRR9045295
SRR9045294
SRR9045298
SRR9045297
SRR11567260
SRR10854653
SRR11567261
SRR9045293
SRR11567157
SRR9045296
SRR9045291
SRR11061154
SRR11061153
SRR9045292
SRR11828593
SRR11829269
SRR11828899
SRR11829104
SRR11828800
SRR11829000
SRR11829268
SRR11828889
SRR11829102
SRR12659820
Length
GC-content
Samples
150,000,000
QC Reads
0
125,000
Total reads mapped
0
0.35
Percentage
0
1685
Num SNVs
0
1411
Num SCVs
0
9
Num INDELs
0
Metagenomic Samples
ALSA (6)
ALJR (8)
MASP (5)
MASA (5)
Pangenomics Analysis
Num genes in GC
Max num paralogs
Comb. Homogeneity Ind.
COG20 FUNCTION
Reference
SRR9045293
SRR11567260
SRR9045294
SRR9045297
SRR11567157
SRR10854653
SRR9045298
SRR9045291
SRR9045296
SRR9045292
SRR9045295
SRR11567261
SRR11061153
SRR11061154
SRR11828899
SRR11829104
SRR11829268
SRR11829000
SRR11828800
SRR12659820
Assembly
150000000
QCReads
0
100
CheckM
0
100
Completion
0
100
Redundancy
0
3380
Num gene clusters
0
32
Singleton gene clusters
0
4155519
Total length
0
Primary MAG
Reassembled MAGs
ALSA (6)
ALJR (8)
MASA (4)
MASP (2)

Figure S11

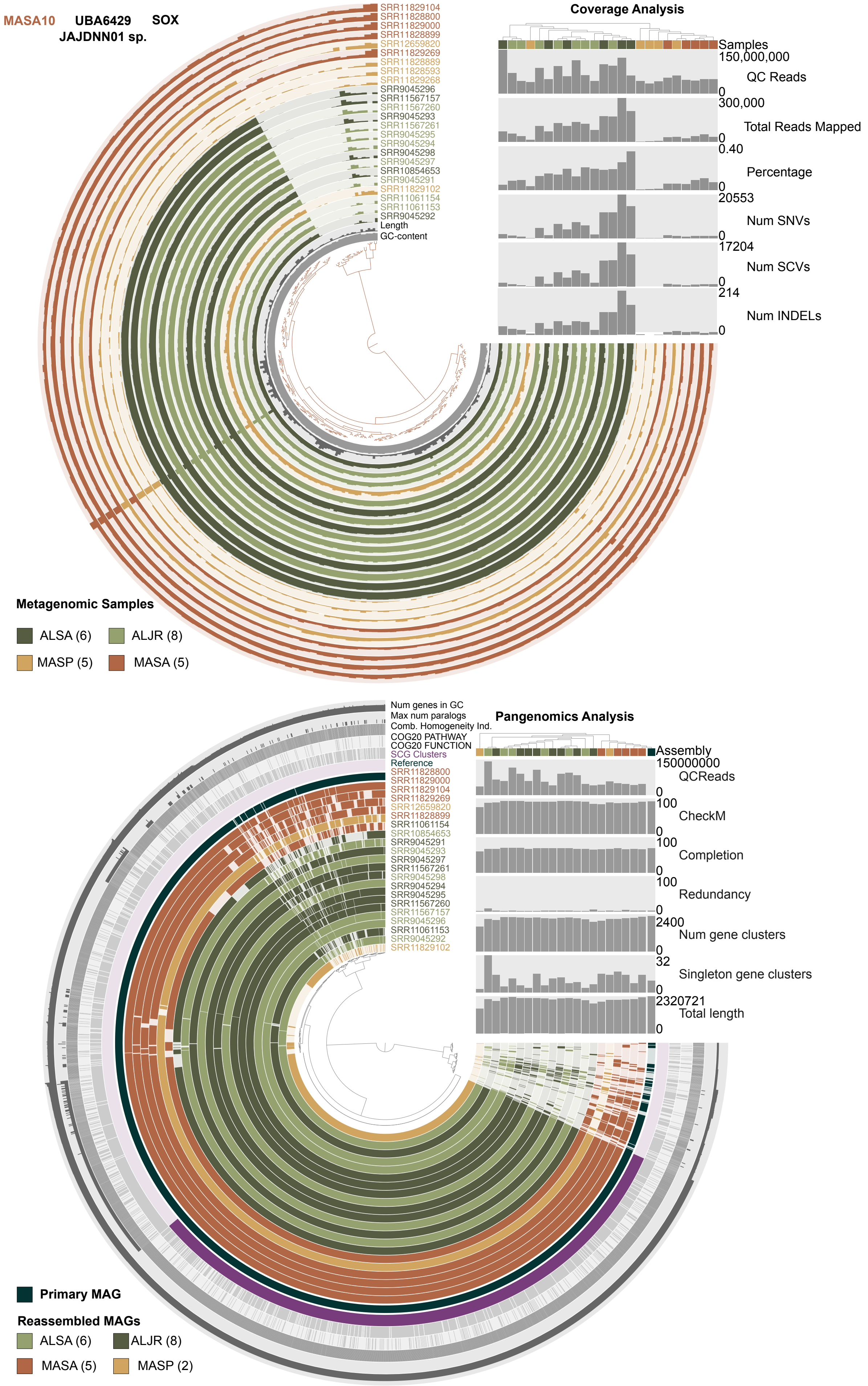

MASA10
UBA6429
SOX
JAJDNN01 sp.
Coverage Analysis
SRR11829104
SRR11828800
SRR11829000
SRR11828899
SRR12659820
SRR11829269
SRR11828889
SRR11828593
SRR11829268
SRR9045296
SRR11567157
SRR11567260
SRR9045293
SRR11567261
SRR9045295
SRR9045294
SRR9045298
SRR9045297
SRR10854653
SRR9045291
SRR11829102
SRR11061154
SRR11061153
SRR9045292
Length
GC-content
Samples
150,000,000
QC Reads
0
300,000
Total Reads Mapped
0
0.40
Percentage
0
20553
Num SNVs
0
17204
Num SCVs
0
214
Num INDELs
0
Metagenomic Samples
ALSA (6)
ALJR (8)
MASP (5)
MASA (5)
Num genes in GC
Max num paralogs
Comb. Homogeneity Ind.
COG20 PATHWAY
COG20 FUNCTION
SCG Clusters
Reference
SRR11828800
SRR11829000
SRR11829104
SRR11829269
SRR12659820
SRR11828899
SRR11061154
SRR10854653
SRR9045291
SRR9045293
SRR9045297
SRR11567261
SRR9045298
SRR9045294
SRR9045295
SRR11567260
SRR11567157
SRR9045296
SRR11061153
SRR9045292
SRR11829102
Pangenomics Analysis
Assembly
150000000
QCReads
0
100
CheckM
0
100
Completion
0
100
Redundancy
0
2400
Num gene clusters
0
32
Singleton gene clusters
0
2320721
Total length
0
Primary MAG
Reassembled MAGs
ALSA (6)
ALJR (8)
MASA (5)
MASP (2)

Figure S12

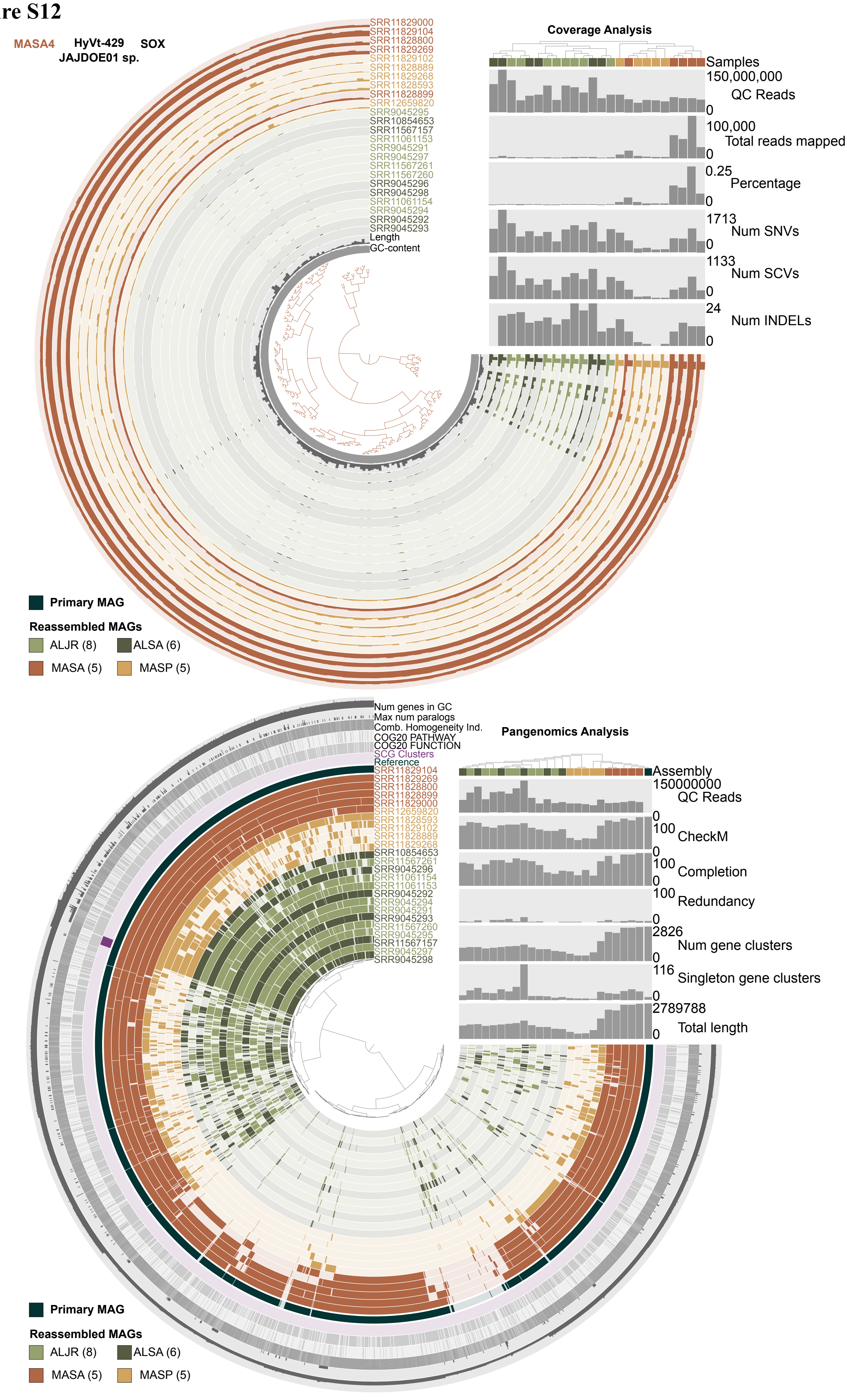
MASA4
HyVt-429
SOX
JAJDOE01 sp.
Coverage Analysis
SRR11829000
SRR11829104
SRR11828800
SRR11829269
SRR11829102
SRR11828889
SRR11829268
SRR11828593
SRR11828899
SRR12659820
SRR9045295
SRR10854653
SRR11567157
SRR11061153
SRR9045291
SRR9045297
SRR11567261
SRR11567260
SRR9045296
SRR9045298
SRR11061154
SRR9045294
SRR9045292
SRR9045293
Length
GC-content
Samples
150,000,000
QC Reads
0
100,000
Total reads mapped
0
0.25
Percentage
0
1713
Num SNVs
0
1133
Num SCVs
0
24
Num INDELs
0
Primary MAG
Reassembled MAGs
ALJR (8)
ALSA (6)
MASA (5)
MASP (5)
Num genes in GC
Max num paralogs
Comb. Homogeneity Ind.
COG20 PATHWAY
COG20 FUNCTION
SCG Clusters
Reference
Pangenomics Analysis
SRR11829104
SRR11829269
SRR11828800
SRR11828899
SRR11829000
SRR12659820
SRR11828593
SRR11829102
SRR11828889
SRR11829268
SRR10854653
SRR11567261
SRR9045296
SRR11061154
SRR11061153
SRR9045292
SRR9045294
SRR9045291
SRR9045293
SRR11567260
SRR9045295
SRR11567157
SRR9045297
SRR9045298
Assembly
150000000
QC Reads
0
100
CheckM
0
100
Completion
0
100
Redundancy
0
2826
Num gene clusters
0
116
Singleton gene clusters
0
2789788
Total length
0
Primary MAG
Reassembled MAGs
ALJR (8)
ALSA (6)
MASA (5)
MASP (5)

Figure S13

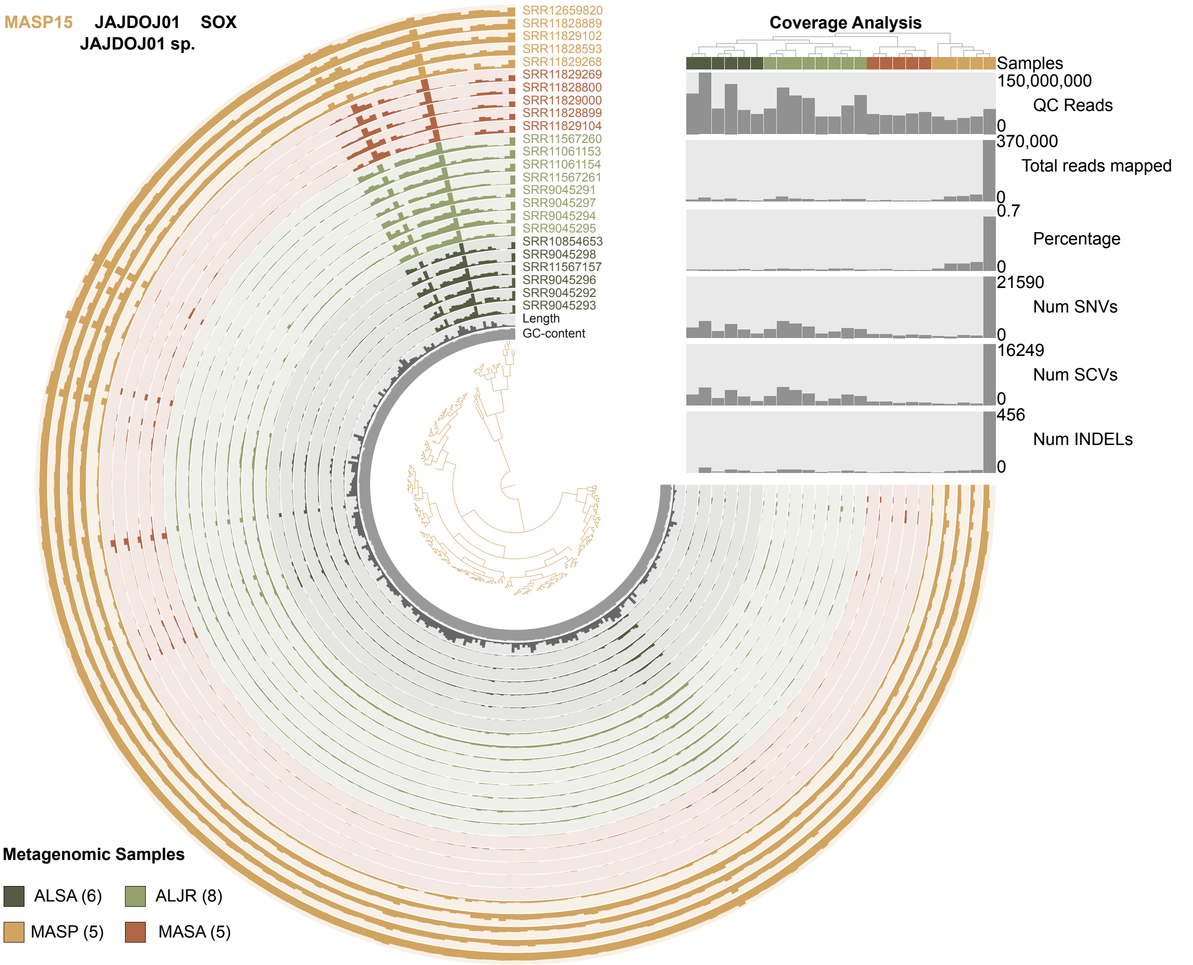

MASP15 JAJDOJ01 SOX
JAJDOJ01 sp.
SRR12659820
SRR11828889
SRR11829102
SRR11828593
SRR11829268
SRR11829269
SRR11828800
SRR11829000
SRR11828899
SRR11829104
SRR11567260
SRR11061153
SRR11061154
SRR11567261
SRR9045291
SRR9045297
SRR9045294
SRR9045295
SRR10854653
SRR9045298
SRR11567157
SRR9045296
SRR9045292
SRR9045293
Length
GC-content
Coverage Analysis
Samples
150,000,000
QC Reads
0
370,000
Total reads mapped
0
0.7
Percentage
0
21590
Num SNVs
0
16249
Num SCVs
0
456
Num INDELs
0
Metagenomic Samples
ALSA (6)
ALJR (8)
MASP (5)
MASA (5)

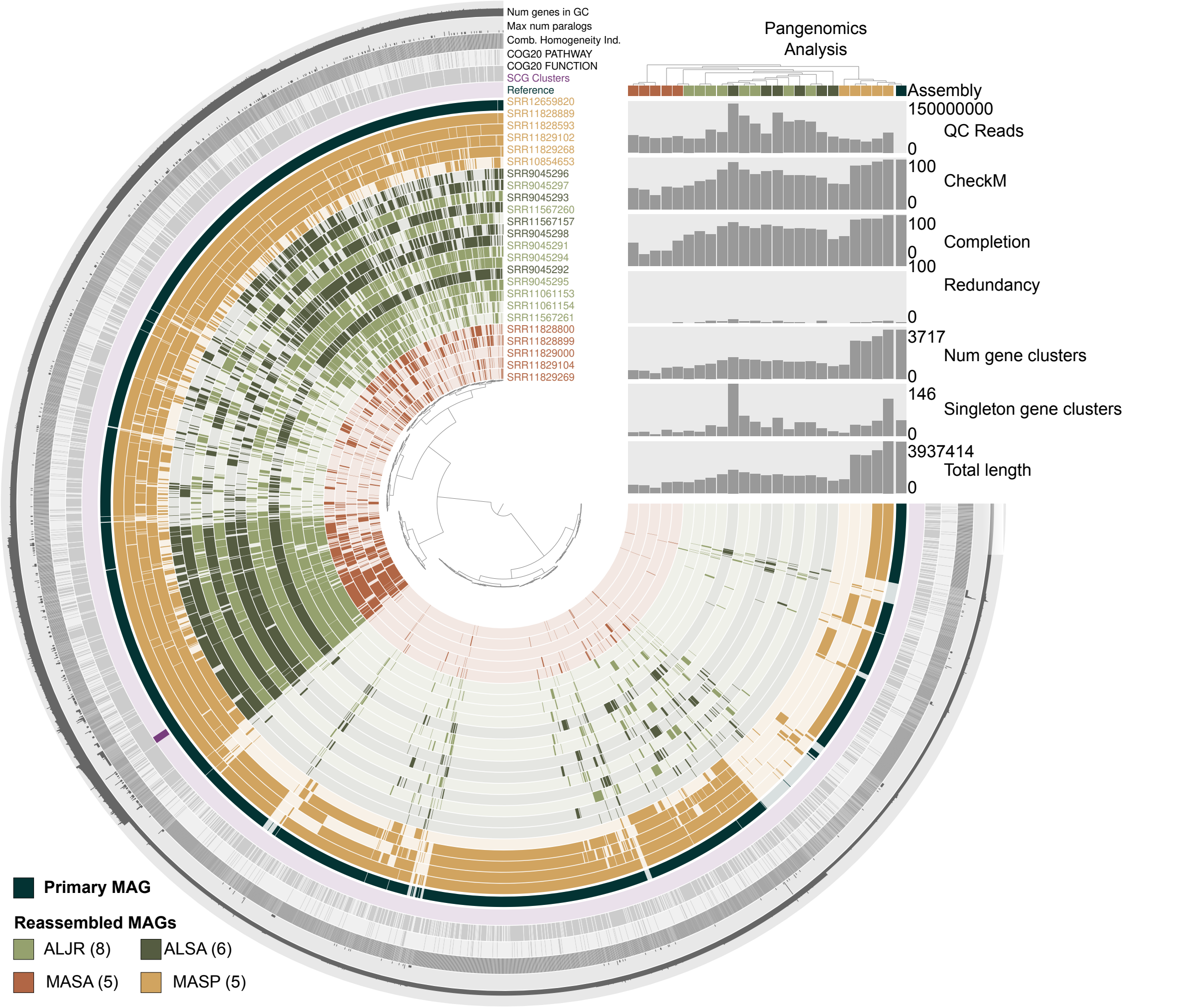

Num genes in GC
Max num paralogs
Comb. Homogeneity Ind.
COG20 PATHWAY
COG20 FUNCTION
SCG Clusters
Reference
SRR12659820
SRR11828889
SRR11828593
SRR11829102
SRR11829268
SRR10854653
SRR9045296
SRR9045297
SRR9045293
SRR11567260
SRR11567157
SRR9045298
SRR9045291
SRR9045294
SRR9045292
SRR9045295
SRR11061153
SRR11061154
SRR11567261
SRR11828800
SRR11828899
SRR11829000
SRR11829104
SRR11829269
Pangenomics Analysis
Assembly
150000000
QC Reads
0
100
CheckM
0
100
Completion
0
100
Redundancy
0
3717
Num gene clusters
0
146
Singleton gene clusters
0
3937414
Total length
0
Primary MAG
Reassembled MAGs
ALJR (8)
ALSA (6)
MASA (5)
MASP (5)

Figure S14

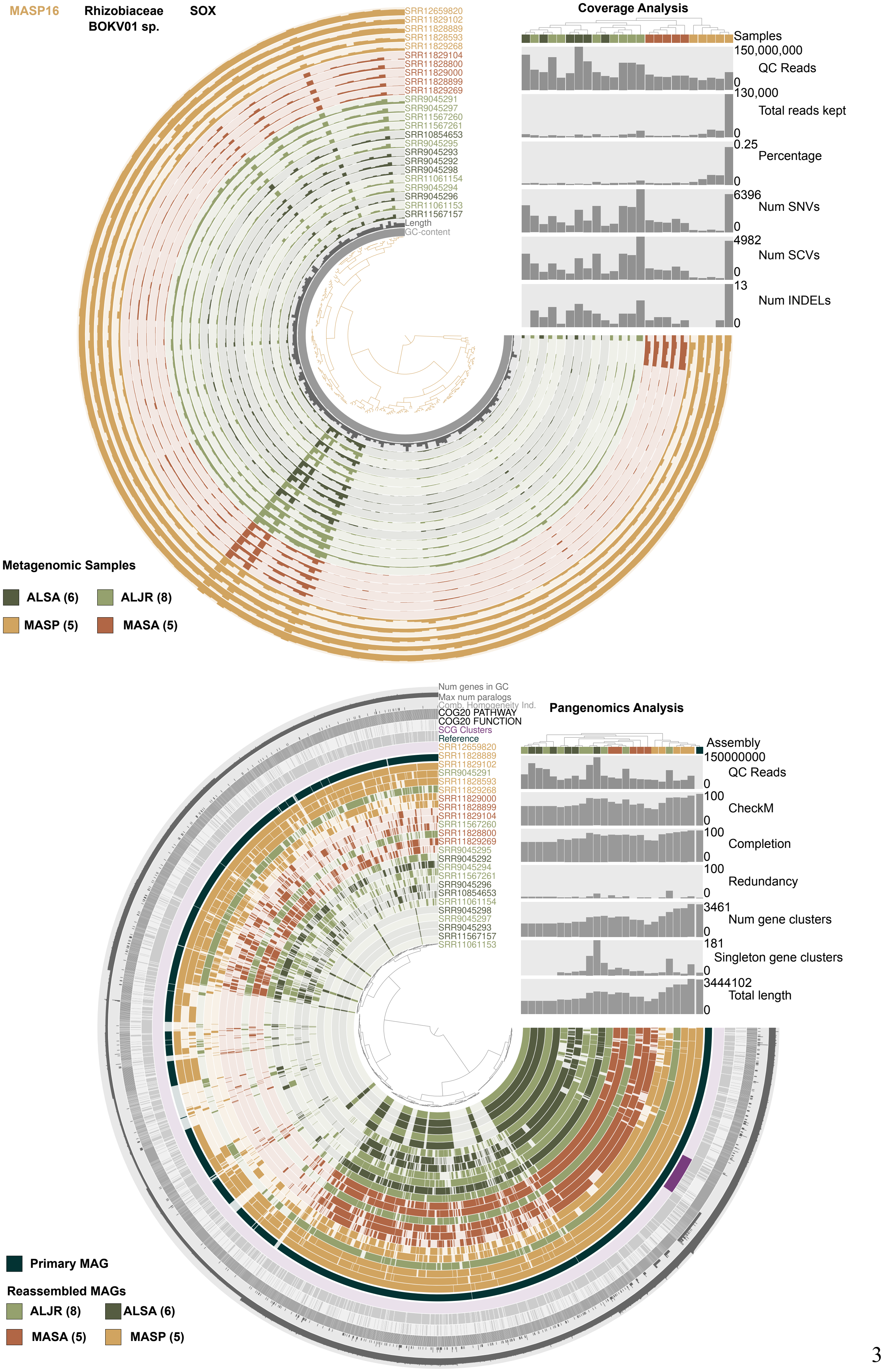

MASP16
Rhizobiaceae
BOKV01 sp.
SOX
Coverage Analysis
SRR12659820
SRR11829102
SRR11828889
SRR11828593
SRR11829268
SRR11829104
SRR11828800
SRR11829000
SRR11828899
SRR11829269
SRR9045291
SRR9045297
SRR11567260
SRR11567261
SRR10854653
SRR9045295
SRR9045293
SRR9045292
SRR9045298
SRR11061154
SRR9045294
SRR9045296
SRR11061153
SRR11567157
Length
GC-content
Samples
150,000,000
QC Reads
0
130,000
Total reads kept
0
0.25
Percentage
0
6396
Num SNVs
0
4982
Num SCVs
0
13
Num INDELs
0
Metagenomic Samples
ALSA (6)
ALJR (8)
MASP (5)
MASA (5)
Num genes in GC
Max num paralogs
Comb. Homogeneity Ind.
COG20 PATHWAY
COG20 FUNCTION
SCG Clusters
Reference
SRR12659820
SRR11828889
SRR11829102
SRR9045291
SRR11828593
SRR11829268
SRR11829000
SRR11828899
SRR11829104
SRR11567260
SRR11828800
SRR11829269
SRR9045295
SRR9045292
SRR9045294
SRR11567261
SRR9045296
SRR10854653
SRR11061154
SRR9045298
SRR9045297
SRR9045293
SRR11567157
SRR11061153
Pangenomics Analysis
Assembly
150000000
QC Reads
0
100
CheckM
0
100
Completion
0
100
Redundancy
0
3461
Num gene clusters
0
181
Singleton gene clusters
0
3444102
Total length
0
Primary MAG
Reassembled MAGs
ALJR (8)
ALSA (6)
MASA (5)
MASP (5)

Figure S15. Distribution, across the 24 samples, of eight sulfur cycling primary MAG sequences selected for further analyses. A) Detail of Fig. 4a highlighting the MAGs used for the Anvi'o-based analysis. Quantification was by read-mapping AL and MA samples to the contigs of the primary MAGs, with coverage standardized by library size and contig length and expressed as genome copies per million (GCPM). B) Results of Bowtie read recruitment expressed as million reads mapped (left) and the percentage of reads of a given metagenomic sample recruited to each of the MAGs (right). C) Example relationships between sampling depth (as the number of reads recruited after mapping) and the number of detected SNVs for two of the eight MAGs. Legend as for panel A) (Additional graphs are presented in Supplemental Data 5).

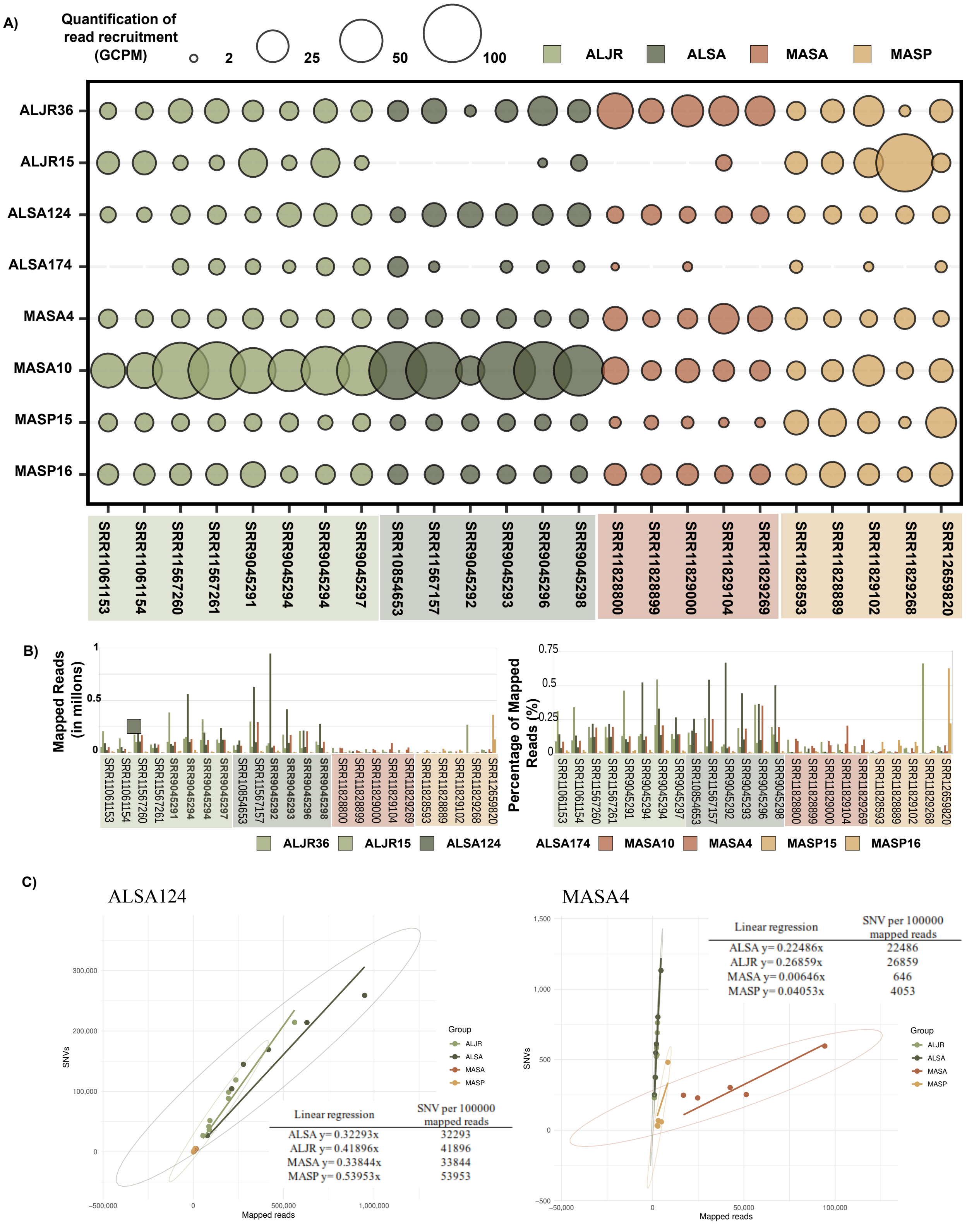

Figure S16. Squeme of the Reverse Citrate Cycle (Arnon-Buchanan) and Phosphate acetyltransferase-acetate kinase pathways. The enzymes detected in the reference-guided reassembled metagenomes corresponding to the group ALJR36 are indicated in dark grey and those in the MASA10 reassembled group are in light grey. Adapted from Kegg map (00720) representing the Carbon Fixation Pathways in Prokaryotes.

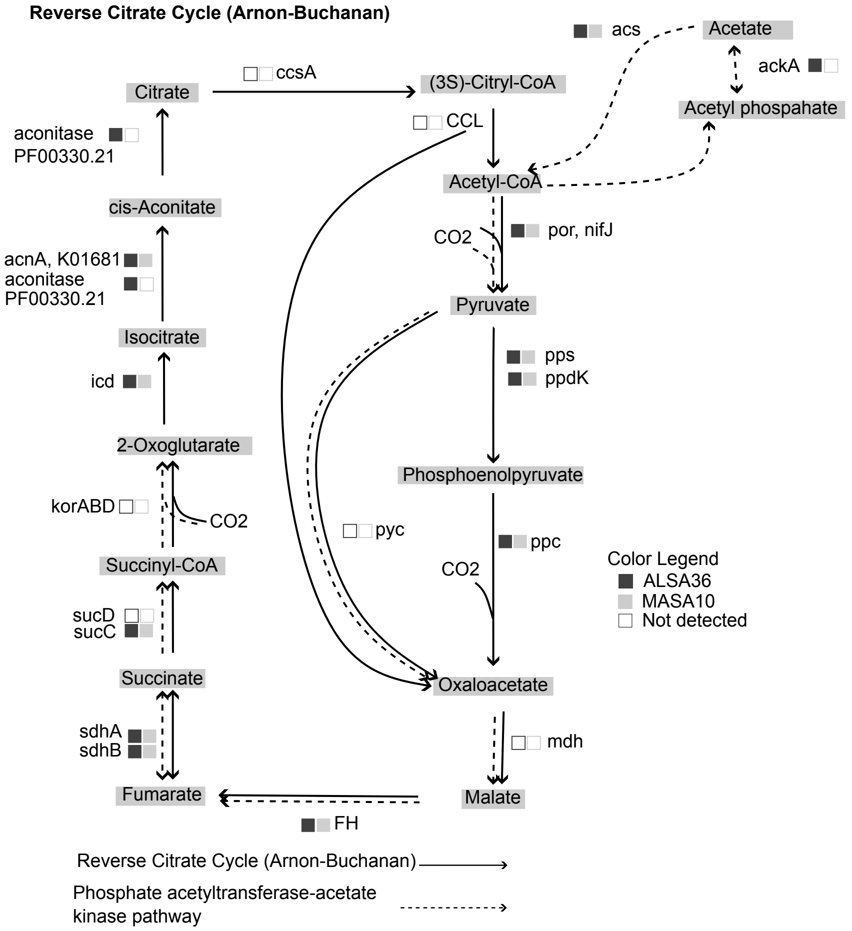

Figure S17. Phylogeny reconstructed for MA/AL DGRs. Reverse transcriptase sequences were aligned with representative RTs from previously identified DGRs in Paul et al. (2017). The extracted subtree shows closely related DGR-RTs from this study (dashed turquoise box) and other bacterial clades. Scale bar indicates substitutions per site.

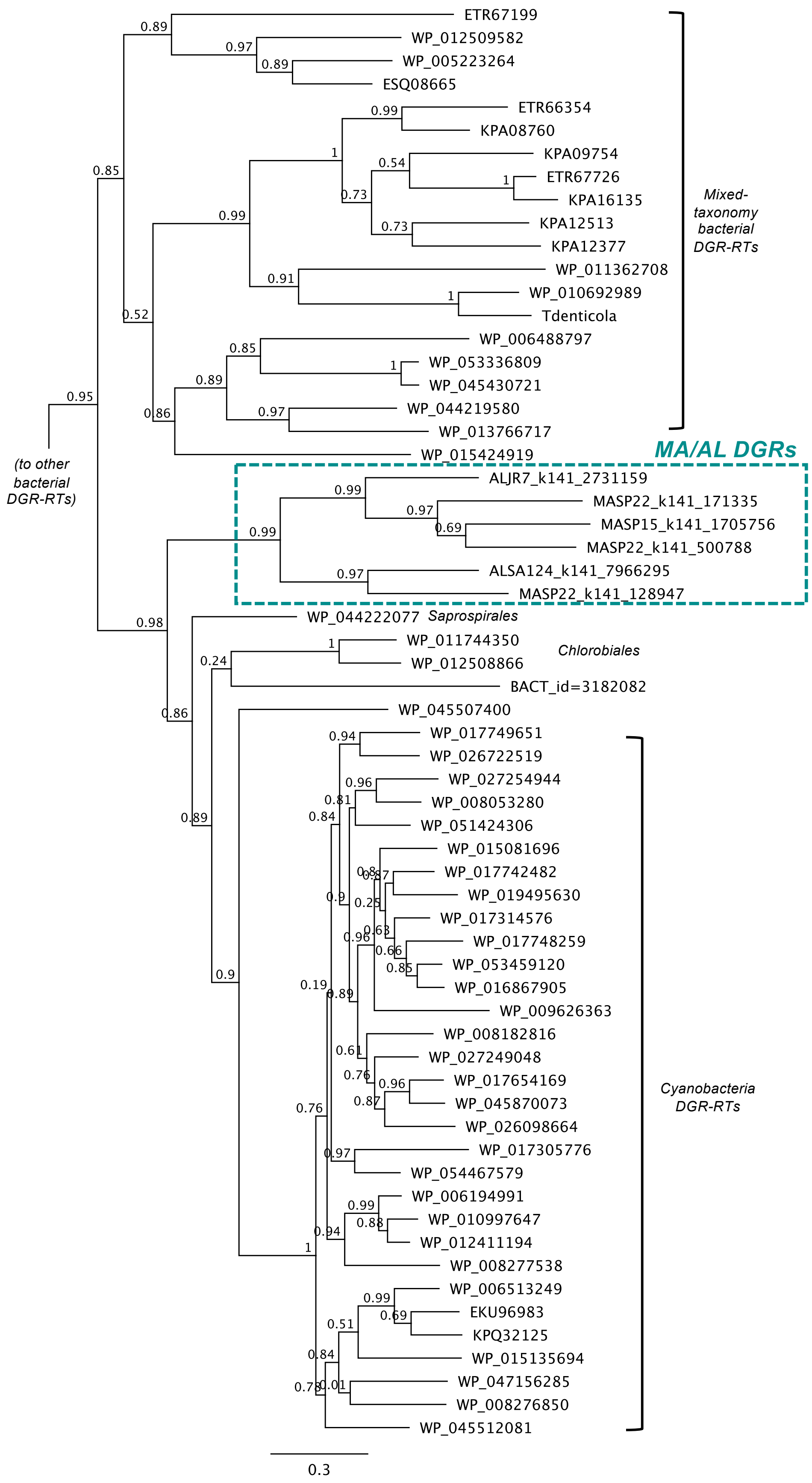

Figure S18. A) Coverage (left) and gene variability (right) of selected genes. In the heatmaps, each row represents a gen (see Supplementary Data 9 for the full list of genes), and each column is a metagenomic sample. Genes with average coverage under 5 were excluded from the analysis and are indicated in gray. Gene variability was calculated as average gene entropy from single codon variants (SCVs) and results are summarized in Supplemental Data 9. B) Comparison of coverage and entropy values for the two closely-related MAGs ALJR36 and MASA10.

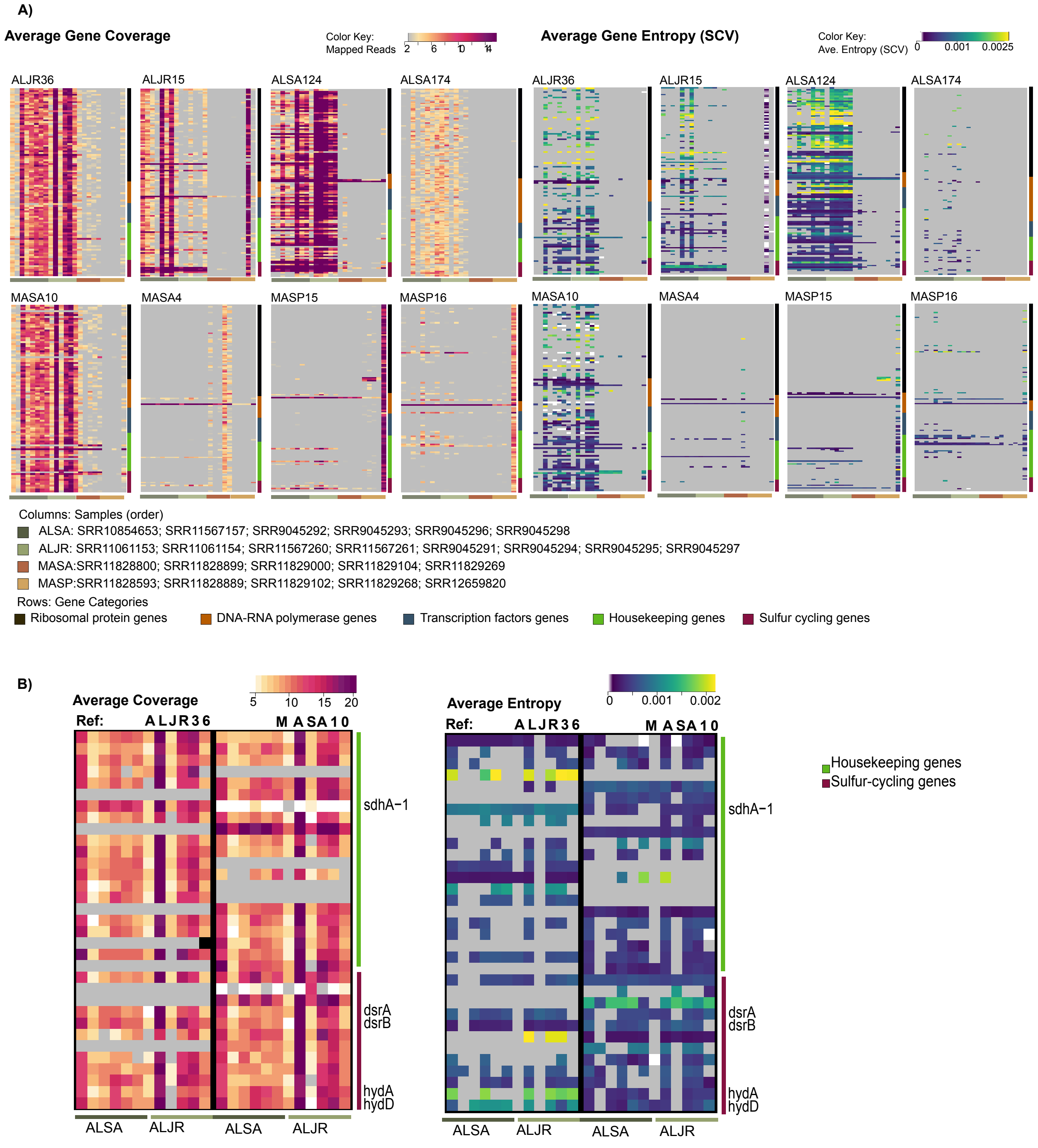

Supplement: Supplemental Material — Supplemental methods and results, Tables S1 to S9, supplemental data file descriptions, and Fig. S1 to S18. [file aem.00988-23-s0010.pdf]
